# Computational Identification of Diverse Mechanisms Underlying Transcription Factor-DNA Occupancy

**DOI:** 10.1371/journal.pgen.1003571

**Published:** 2013-08-01

**Authors:** Qiong Cheng, Majid Kazemian, Hannah Pham, Charles Blatti, Susan E. Celniker, Scot A. Wolfe, Michael H. Brodsky, Saurabh Sinha

**Affiliations:** 1 Department of Computer Science, University of Illinois at Urbana-Champaign, Urbana, Illinois, United States of America; 2 Program in Gene Function and Expression, University of Massachusetts Medical School, Worcester, Massachusetts, United States of America; 3 Department of Genome Dynamics, Berkeley Drosophila Genome Project, Lawrence Berkeley National Laboratory, Berkeley, California, United States of America; 4 Department of Biochemistry and Molecular Pharmacology, University of Massachusetts Medical School, Worcester, Massachusetts, United States of America; 5 Department of Molecular Medicine, University of Massachusetts Medical School, Worcester, Massachusetts, United States of America; 6 Institute of Genomic Biology, University of Illinois at Urbana-Champaign, Urbana, Illinois, United States of America; Columbia University, United States of America

## Abstract

ChIP-based genome-wide assays of transcription factor (TF) occupancy have emerged as a powerful, high-throughput method to understand transcriptional regulation, especially on a global scale. This has led to great interest in the underlying biochemical mechanisms that direct TF-DNA binding, with the ultimate goal of computationally predicting a TF's occupancy profile in any cellular condition. In this study, we examined the influence of various potential determinants of TF-DNA binding on a much larger scale than previously undertaken. We used a thermodynamics-based model of TF-DNA binding, called “STAP,” to analyze 45 TF-ChIP data sets from *Drosophila* embryonic development. We built a cross-validation framework that compares a baseline model, based on the ChIP'ed (“primary”) TF's motif, to more complex models where binding by secondary TFs is hypothesized to influence the primary TF's occupancy. Candidates interacting TFs were chosen based on RNA-SEQ expression data from the time point of the ChIP experiment. We found widespread evidence of both cooperative and antagonistic effects by secondary TFs, and explicitly quantified these effects. We were able to identify multiple classes of interactions, including (1) long-range interactions between primary and secondary motifs (separated by ≤150 bp), suggestive of indirect effects such as chromatin remodeling, (2) short-range interactions with specific inter-site spacing biases, suggestive of direct physical interactions, and (3) overlapping binding sites suggesting competitive binding. Furthermore, by factoring out the previously reported strong correlation between TF occupancy and DNA accessibility, we were able to categorize the effects into those that are likely to be mediated by the secondary TF's effect on local accessibility and those that utilize accessibility-independent mechanisms. Finally, we conducted *in vitro* pull-down assays to test model-based predictions of short-range cooperative interactions, and found that seven of the eight TF pairs tested physically interact and that some of these interactions mediate cooperative binding to DNA.

## Author Summary

Chromatin Immunoprecipitation (ChIP)-based genome-wide assays of transcription factor (TF) occupancy have emerged as a powerful, high throughput method to understand transcriptional regulation, especially on a global scale. Here, we utilize 45 ChIP-chip and ChIP-SEQ data sets from *Drosophila* to explore the underlying mechanisms of TF-DNA binding. For this, we employ a biophysically motivated computational model, in conjunction with over 300 TF motifs (binding specificities) as well as gene expression and DNA accessibility data from different developmental stages in *Drosophila* embryos. Our findings provide robust statistical evidence of the role played by TF-TF interactions in shaping genome-wide TF-DNA binding profiles, and thus in directing gene regulation. Our method allows us to go beyond simply recognizing the existence of such interactions, to quantifying their effects on TF occupancy. We are able to categorize the probable mechanisms of these effects as involving direct physical interactions versus accessibility-mediated indirect interactions, long-range versus short-range interactions, and cooperative versus antagonistic interactions. Our analysis reveals widespread evidence of combinatorial regulation present in recently generated ChIP data sets, and sets the stage for rich integrative models of the future that will predict cell type-specific TF occupancy values from sequence and expression data.

## Introduction

A major challenge in the analysis of genomic sequences is the annotation of *cis*-regulatory elements. Significant progress has been made towards this goal through high throughput methods such as ChIP-chip and ChIP-SEQ that describe the locations where specific transcription factors (TFs) bind to the genome *in vivo* [1]–[3]. ChIP-based characterization of TF binding profiles can help elucidate specific regulatory interactions between TFs and genes [4]. A number of genome-wide ChIP data sets, corresponding to diverse TFs and cellular conditions, have been generated through the efforts of various laboratories and consortia [1], [3]. Such data sets also offer the opportunity to apply computational and statistical methods to understand the determinants of TF-DNA binding at a quantitative level [5]–[7]. Given the central role of the TF-DNA binding process in the regulatory activity of a TF, such an understanding can provide a holistic view of transcriptional regulation and also set the stage for future computational methods for predicting cell type-specific TF-binding profiles.

The most extensively studied determinant of TF occupancy is the DNA binding specificity of the TF. Various experimental approaches [8]–[11] have been successful in obtaining motifs representing the diversity and relative affinities of DNA sequences bound by an individual TF. An initial expectation is that a TF's motif will allow prediction of its binding levels genome-wide. On the other hand, it is clear that interactions with other TFs can significantly influence binding to regulatory sequences. For example, interaction of Hox proteins with a cofactor results in greater DNA binding specificity [12] and the tramtrack (TTK) protein can regulate transcription independent of its own DNA binding domain via its interaction with the Trithorax-like (TRL, also known as GAGA binding factor) [13]. Furthermore, TF occupancy at a genomic location in a given cell type also depends on the concentration of that TF, as well as the motifs and concentrations of other TFs that might facilitate or inhibit DNA-binding at the location [14].

A number of recent studies have used genome-wide datasets to characterize parameters that correlate with TF occupancy. In several studies, genome-wide measurements of *in vivo* DNA accessibility were tested for the ability to help describe TF ChIP data. These studies clearly demonstrate that TF occupancy has a close relationship with *in vivo* DNA accessibility [6], [7], with both factors likely influencing each other [6], [15]–[19]. While these studies reveal that experimental analysis of accessibility can improve modeling of ChIP data, they do not reveal the underlying genomic sequence features that contribute to accessibility. In another study [5], sequence motifs experimentally and computationally identified in *Drosophila* were shown to contribute to context-specific TF occupancy. Application of discriminative motif analysis to a TF assayed across multiple conditions can successfully identify predictive motifs associated with context-specific binding. However, whether TFs bound to these discriminative motifs contribute to occupancy by direct interaction with the primary TF, accessibility or other mechanisms is not assessed.

In this work, we test the influence of various potential sequence determinants of *in vivo* TF-DNA binding – the TF's binding motif, as well as the positive or negative influence of other TFs binding in the vicinity – on each of 45 TF-ChIP data sets in *Drosophila.* For this analysis, we took advantage of over 300 distinct DNA binding specificity motifs determined for individual TFs [20], which encompasses approximately 40% of all predicted *Drosophila* TFs, and relied upon stage-specific whole-genome RNA-SEQ data [21] to determine which secondary TFs are expressed at the time of the ChIP experiment. We follow the general framework proposed by Kaplan et al. [6], which involves: (1) building computational models that predict TF binding at a location, and (2) assessing how well a baseline model that only uses the “primary motif” (i.e., binding motif of the “ChIP'ed” TF) fits the ChIP data, as compared with more complex models that incorporate additional determinants such as motifs for additional secondary TFs (i.e., TFs other than the ChIP'ed TF). We use the biophysical model STAP [22] to perform these tests. Improvements in the goodness-of-fit measure are evaluated statistically, and a cross-validation framework is adopted to account for differing model complexity in the comparisons. We evaluate each potential determinant separately in order to limit the number of free parameters in the models. For each identified secondary TF, we performed statistical tests to categorize the mechanistic basis of its contribution. In particular, we asked if a secondary motif's influence is likely to be (a) through long-range (≤150 bp) versus short-range (≤30 bp) interactions with the primary motif, (b) through synergistic or antagonistic interactions, and (c) through modulation of local DNA accessibility or direct interactions between TFs.

We find widespread evidence of the effect of secondary TFs on the primary (ChIP'ed) TF's binding levels, including both enhanced occupancy (“cooperativity”) and reduced occupancy (“antagonism”). Cooperative and antagonistic influences of secondary motifs can act through: 1) long-range interactions between primary and secondary motifs, suggesting indirect effects such as chromatin remodeling, 2) short-range interactions with specific inter-site spacing biases, suggesting a direct association, or 3) through overlapping binding sites, suggesting competition for site occupancy.

Two types of experimental evidence support our computational assignments of secondary TFs that influence occupancy via local chromatin architecture or cooperative DNA binding. Extending previous observations [6], [7], we find that DNA accessibility is the primary genomic feature correlated with TF occupancy across the majority of the 45 data sets examined here. We then use accessibility data to re-examine secondary TF motifs that improved prediction of ChIP data in our accessibility-agnostic analysis. We identify several secondary motifs whose contribution is reduced or lost when accessibility information is part of the model, suggesting that the secondary TF influences binding mainly by modulating accessibility patterns. The TFs vielfaltig (VFL, also known as Zelda) and TRL (also known as GAGA factor) appear to synergistically influence the binding of several primary TFs in early and mid-stage embryonic development respectively. Interestingly, the influence of VFL is sometimes imposed through accessibility, while in other cases it is independent of accessibility. In contrast, the influence of TRL is imposed exclusively through accessibility. The TF motifs for extradenticle (EXD), retained (RETN), jing interacting gene regulatory 1 (JIGR1) and homothorax (HTH) commonly antagonize TF occupancy through accessibility-mediated and accessibility-independent mechanisms. We find many cases where the secondary motif's influence remains significant upon accounting for accessibility, thus suggesting alternative mechanisms such as cooperative or antagonistic DNA-binding by the primary and secondary TFs. We identify eight examples where the arrangement of primary and secondary motifs implies cooperative binding via physical interaction, and demonstrate that for all but one of these cases the TFs do, in fact, directly interact *in vitro* and that several bind cooperatively *in vitro* to sequences that are occupied *in vivo*. Overall, our analysis demonstrates that a biophysical model for the combinatorial action of primary and secondary TFs used with an extensive collection of binding motifs for known TFs can describe the mechanistic basis for *in vivo* patterns of TF occupancy.

## Results

### Model evaluation and baseline results using the ChIP'ed transcription factor's motif

**Figure 1:**
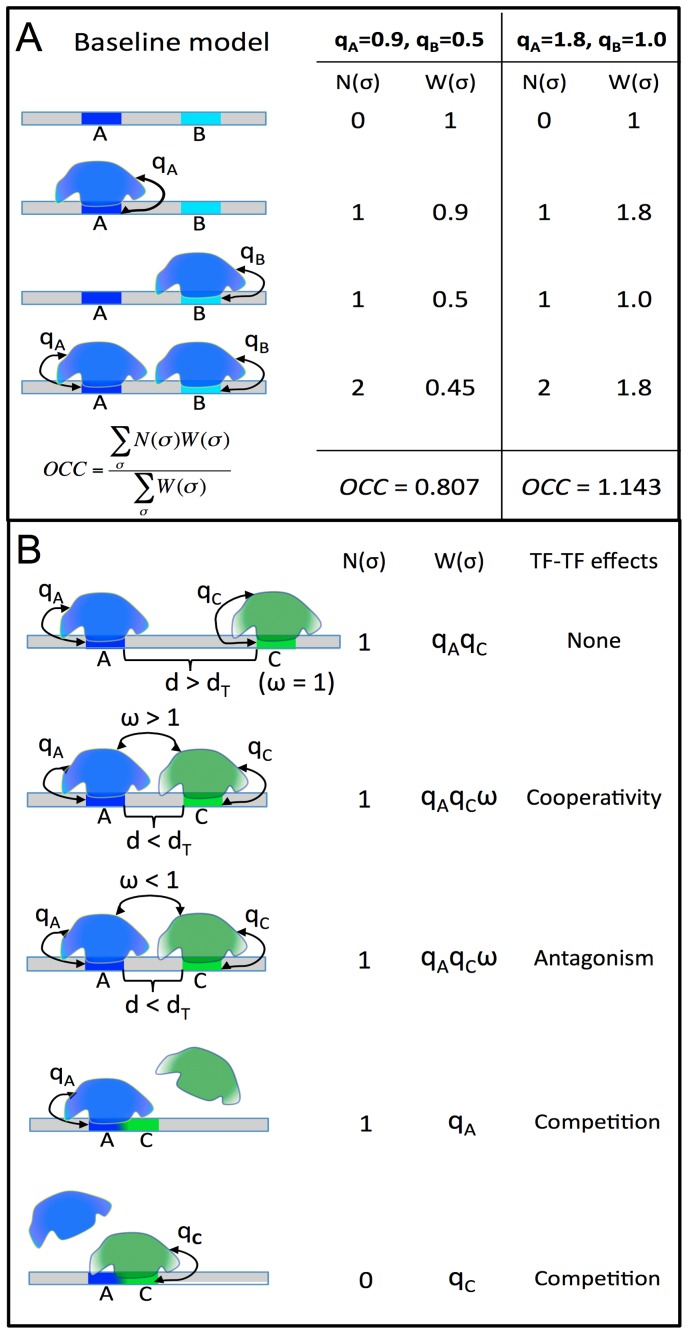
STAP model of TF-DNA binding. **A.** Baseline model: only the ChIP'ed (“primary”) TF is considered, and its putative sites in the given sequence are identified. Here, A is a strong site and B is a medium strength site of the TF. Four possible configurations (σ) of A and/or B bound by the TF are enumerated, and for each σ the relative weight W(σ) is calculated as the product of terms (q_A_, q_B_) specific to sites occupied in that configuration. The occupancy is then estimated as a weighted average of N(σ), the number of occupied sites in σ. The site-specific terms q_A_, q_B_ are proportional to TF concentration, so a doubling of concentration will change (q_A_ = 0.9, q_B_ = 0.5) to (q_A_ = 1.8, q_B_ = 1.0), and this will impact the predicted occupancy (OCC = 0.807 to OCC = 1.143), but does not double the prediction, due to saturation effects. **B.** Interaction between the primary TF (blue) and a secondary TF (green) is modeled by re-defining the relative weight of a configuration where both sites are bound. If the two sites are separated by more than some distance threshold d_T_, nothing changes, and there is no interaction. If the separation is less than d_T_, the relative weight of σ is multiplied by an interaction term ω, which can be >1 (for cooperative influence) or <1 (for antagonistic influence). This increases or decreases (respectively) the probability of the joint configuration, and therefore the overall occupancy of the primary TF at site A. Competitive binding at overlapping sites A, C is modeled automatically, since both sites may not be occupied simultaneously in any configuration.

We began our analysis with fifty-five TF-ChIP data sets obtained from diverse sources (see Methods). Each TF-ChIP data set was represented by 1000 peaks and 1000 random non-coding sequence windows, all of length 500 bp. This representation was selected with the goal of identifying TF motifs that improve the ability to properly rank the occupancy within the peak group and/or improve the ability to discriminate between peaks and random sequences. The average ChIP score of each window was treated as the TF occupancy level in that window (Methods), and is henceforth called the “ChIP score”. For each data set a Position Weight Matrix (PWM or motif) representing the DNA binding specificity of the ChIP'ed TF was identified (Methods) and designated as the “primary motif”. We used the STAP program [22] (Figure 1A) to predict a binding level, henceforth called “STAP score”, for each sequence window in a data set, using the primary motif from that data set. We then computed the Pearson Correlation Coefficient (CC) between ChIP scores and STAP scores across the 2000 windows in each data set, and call this the “baseline CC” for the data set. This value captures the ability of the primary TF's binding motif to determine that TF's relative occupancy levels both within the most highly bound regions and in peak versus non-peak regions. Since STAP has one free parameter for which it requires training data (sequences and their binding levels), we performed 4-fold cross-validation to obtain STAP scores for all 2000 windows, with 500 test windows in each fold. Out of the 55 data sets, seven did not show a sufficiently high correlation (CC ≥0.15 and p-value<1E-11) here or in any other test that we report in the following sections, and three data sets presented technical problems in the training phase, e.g., inconsistent parameter values learned over different folds of cross-validation. These 10 data sets (Supplementary Table S1) are excluded from the rest of our report. In all of these examples, the TF motif is broadly confirmed by similarity to motifs for the same TF obtained by other methods or to motifs for homologous TFs. Thus, the low correlation may reflect a high degree of recruitment to DNA by other proteins, technical problems with this group of ChIP datasets, or with the model as applied to these datasets.

**Figure 2:**
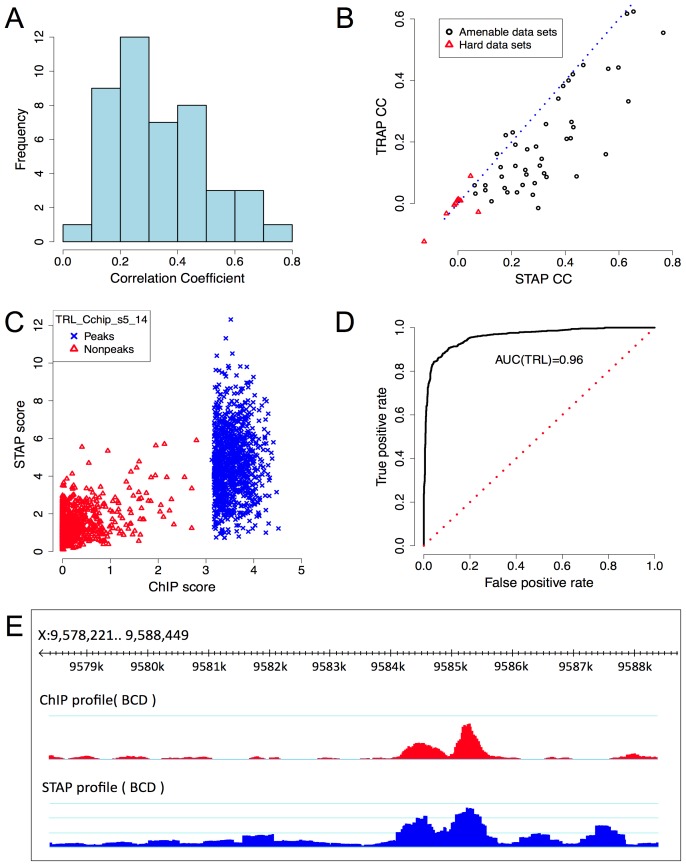
Baseline model performance. **A.** Histogram of CC values from Table 1. **B.** Comparison of CC from STAP-based motif scores to those from TRAP-based motif scores, on all 55 data sets that were examined. Red triangular symbols represent the ten data sets that showed poor CC in all of our models and were thus excluded from reported results. **C.** ChIP scores and STAP scores for all 2000 segments in the data set that had the best CC overall: “TRL_Cchip_s5_14”. Blue and red points represent the 1000 top ChIP peaks and 1000 randomly selected non-coding segments respectively. **D.** Receiver Operating Characteristic (ROC) curve for a classifier that uses a threshold on the STAP score to discriminate TF-bound segments from non-bound segments, defined by the top 50% and bottom 50% ChIP scores in the data set “TRL_Cchip_s5_14”. The area under this curve (AUC) is 0.960. **E.** ChIP (red) and STAP (blue) predicted ChIP score profiles for the transcription factor BCD on a ∼10 Kbp region near gene BTD, at the developmental stage 5. The Pearson's CC at this locus is 0.80.

The results of this first exercise are shown in Table 1 and Figure 2A. We noted the baseline CC in this test to be ≥0.15 (p-value<1E-11) for 39 data sets, with the highest CC reported for the data set “TRL_Cchip_s5_14”, i.e., ChIP-chip data for the TF TRL in stage 5–14 embryo, obtained from the (C)avalli laboratory (see Table 1 legend for data set nomenclature scheme). We repeated this exercise, for all 55 data sets, using a second program, TRAP, also based on a biophysical model of DNA binding [23] with default parameter settings, and noted that CC values from STAP were generally better (Figure 2B), although there were several data sets where the two methods gave almost identical CC values. We also observed from Figure 2B that the ten data sets that we exclude from most of the analyses in this work (red symbols) received poor CC values from both STAP and TRAP. The purpose of this exercise was not to identify a superior method for occupancy prediction; such an attempt would have been biased since we have more experience with STAP than TRAP, and our TRAP analysis was run without training of free parameters. Our goal was to provide evidence that STAP-based predictions provide a reasonable baseline for more advanced models that will be examined below.

**Table 1:** Evaluation of single motif STAP model on 45 TF-ChIP data sets.

Data set	CC(M1)	SPCC(M1)	Data set	CC(M1)	SPCC(M1)	Data set	CC(M1)	SPCC(M1)
TRL_Cchip_s5_14	0.765	*0.593*	TIN_Fchip_s10_11	0.392	**0.445**	DISCO_Mseq_s5_11	0.220	**0.269**
BIN_Fchip_s14	0.654	0.669	HB_Bseq_s5	0.374	0.379	PRD_Bchip_s5	0.214	0.187
MAD_Bchip_s5	0.635	**0.677**	SNA_Bchip_s5	0.330	0.295	SLP1_Bchip_s5	0.214	*0.171*
BIN_Fchip_s10_11	0.630	0.647	D_Mseq_s5_11	0.328	**0.455**	HB_Bchip_s9	0.204	0.177
KR_Mchip_s5_11	0.598	**0.639**	EN_Mchip_s5_14	0.321	0.359	MED_Bchip_s10	0.184	***0.034***
BCD_Bseq_s5	0.560	**0.614**	H_Bchip_s5	0.312	**0.365**	CAD_Bseq_s5	0.178	**0.283**
HKB_Mseq_s14	0.551	0.551	HKB_Bchip_s5	0.305	0.298	EVE_Mseq_s14	0.174	0.209
Z_Bchip_s5	0.467	0.432	BAB_Mchip_s5_14	0.299	0.285	SENS_Mchip_s9_11	0.163	0.135
UBX_Mchip_s5_14	0.442	0.419	D_Bchip_s5	0.291	***0.238***	TLL_Bchip_s5	0.159	0.187
KR_Bseq_s5	0.430	0.458	DA_Bchip_s5	0.286	0.317	DLL_Mchip_s5_14	0.145	0.171
TIN_Fchip_s9	0.428	**0.507**	UBX_Mchip_s9_11	0.279	0.262	SHN_Bchip_s5	0.125	0.122
VFL_Rchip_s5	0.423	*0.244*	RUN_Bchip_s5	0.258	0.279	KNI_Bchip_s5	0.102	0.136
TWI_Fchip_s9	0.420	*0.367*	MED_Bchip_s14	0.255	*0.102*	MED_Bchip_s5	0.102	*−0.046*
TIN_Fchip_s5	0.412	**0.467**	TWI_Bchip_s5	0.251	0.214	D_Mchip_s5_14	0.065	0.046
TWI_Fchip_s10_11	0.405	0.378	GT_Bseq_s5	0.241	0.260	DL_Bchip_s5	0.062	0.063

Column CC(M1): Pearson's correlation coefficient between ChIP scores and STAP scores on 45 data sets. A correlation coefficient of 0.15 has p-value<1E-11. Note that for 39 of 45 data sets the CC is significant at this level. The TF-specific parameter γ was constrained to be in the range [1, 10^4^] (see text). Results shown are from 4-fold cross-validation. Column SPCC(M1): correlation coefficient after “partialing out” the effect of accessibility scores. Bold and italic fonts indicate cases where SPCC is better or worse respectively than CC.

Data set nomenclature: “TF_SRC_STAGE” represents ChIP data for transcription factor “TF”. “SRC_STAGE” is of the form “[B/M/F/C/R][chip/seq]_sX” or “[B/M/F][chip/seq]_sX_Y”, where “B” represents BDTNP data, “M” represents modENCODE data, “F” represents data from the Furlong laboratory [24], “R” represents data from the Rushlow laboratory [68], “C” represents data from the Cavalli laboratory [62], “chip” represents ChIP-chip, “seq” represents ChIP-seq, while “X” and optionally “Y” indicate developmental stage numbers for the *Drosophila* embryo.

Figure 2C provides a scatter-plot visualization of the STAP results on the data set “TRL_Cchip_s5_14”, which has the best baseline CC value (CC = 0.765). Figure 2D provides an alternative visualization of the same results, as an ROC curve showing how the false positive rate of calling a ChIP peak based on STAP scores varies as we vary the STAP score threshold. We see that 89.7% of the 1000 ChIP peaks can be detected using STAP scores while making 10.3% false positive predictions; the Area Under Curve (AUC) is 0.96. Next to TRL, the TF with the highest CC is biniou (BIN), with the data set “BIN_Fchip_s14” exhibiting a CC of 0.654 and an AUC of 0.895 (Supplementary Figure S1). We note that this data set had been previously observed, in [24], to have a high enrichment of the TF motif in ChIP peaks. The ROC for a data set with a more typical value of CC is shown in Supplementary Figure S2 (CC = 0.305, AUC = 0.679). Figure 2E provides a different visualization of the accuracy of STAP predictions, as genome browser tracks of ChIP and STAP scores for the TF BCD on a single gene locus.

The CC values reported above can arise from differences in STAP scores of peaks and non-peaks in a data set, and from correctly modeling the ChIP scores within peaks and/or non-peaks. To examine the contribution of these two types of agreement between data and model, we separately calculated CC values among the peaks and non-peaks (Supplementary Table S10, Supplementary Figures S5A,B). We found several data sets where a significant overall CC was accompanied by a lower but significant CC within peaks, e.g., BCD_Bseq_s5, where the overall CC is 0.560, and the CC within peaks is 0.466 (Supplementary Figure S5A,C). These are examples where the goodness-of-fit arises from discrimination of peaks and non-peaks as well as from quantitative modeling of binding levels. In a few data sets, the signal appears to arise mainly from separation of peaks and non-peaks, e.g., BIN_Fchip_s14, where the overall CC is 0.654 but the CC within peaks and non-peaks is 0.233 and 0.185 respectively. By and large, the CC values within peaks were higher than those within non-peaks, as expected (Supplementary Figure S5B). Interestingly, for a few data sets the correlation within non-peaks was much greater than within peaks. These include UBX_Mchip_s5_14, EN_Mchip_s5_14, DISCO_Mseq_s5_11, EVE_Mseq_s14, and MAD_Bchip_s5, with peaks of the latter two exhibiting significant negative correlation between STAP predictions and ChIP scores (Supplementary Figure S5D). For these TFs, the motif score may be uniformly high in the top peaks, but help discriminate between very low and intermediate occupancy levels in the randomly selected regions, leading to stronger correlation within non-peaks.

We also repeated the evaluation of the above “baseline” model with a modified definition of data sets: now, the 1000 non-peaks of each data set was replaced with 1000 non-peaks randomly chosen from the ChIP peaks of other TFs. CC values analogous to those of Table 1 (Column “CC(M1)”) were computed and compared to those from Table 1 (Supplementary Table S15 and Figure S9). We observed that for a few data sets the new CC value is lower, suggesting that the primary TF motif in these cases may represent common features of TF bound regions. (We visit these cases in a later section.) We also noted several cases where the CC values were significantly higher when using other TFs' peaks as the non-peaks of a data set (e.g., Supplementary Figure S10). We believe such examples better reveal the role of the primary TF motif in determining the TF-DNA binding strength within accessible regions, since all segments considered in the newly defined data sets are ChIP peaks of some TF. Overall, our analysis of primary TF motif scores in different sets of genomic regions supports the idea that the 2000 regions selected for further study can provide insight into diverse types of mechanisms contributing to *in vivo* TF binding.

### ChIP data supports concentration-dependence of TF-DNA binding

**Figure 3:**
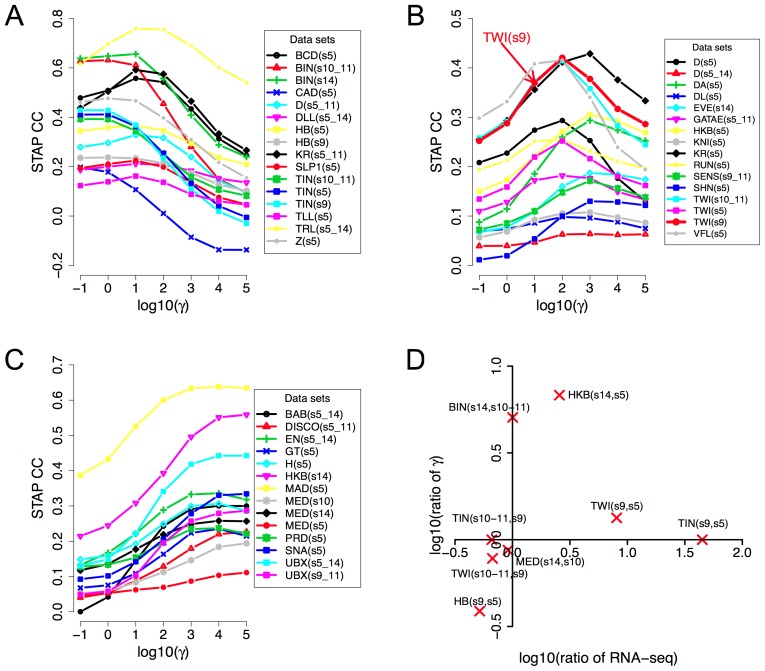
Influence of TF concentration to TF-DNA occupancy. **A–C:** Dependence of correlation coefficient (CC) between ChIP scores and STAP scores (y-axis) on the TF-specific parameter γ that was varied in the range 10^−1^ and 10^5^ (x-axis). All 45 data sets are shown, split into three panels corresponding to cases where the optimal γ was in the range 10^−1^ to 10^1^ (A), 10^2^ to 10^3^ (B), or 10^4^ to 10^5^ (C). The parameter γ in the STAP model reflects the product of the equilibrium constant of the consensus site and the TF's concentration. **D.** Changes in the trained value of the TF-specific parameter γ from one stage to another are consistent with changes in RNA-SEQ-based expression level of the TF. Given a TF for which we have ChIP data from two different developmental stages, the ratio of the trained γ values reflects the ratio of TF concentration in those two stages, as per the model. This ratio is plotted against the ratio of RNA-SEQ levels of the TF's gene from those two developmental stages. All points are in the first or third quadrants suggesting that the trained γ values are consistent with expression data. Each point is labeled by the profiled TF's name and the two corresponding developmental stages.

STAP uses a simple thermodynamic model to define TF-DNA occupancy for a genomic region based on binding site affinities, the equilibrium constant of the TF for its optimal site, and the TF's concentration level [22]. While the binding site affinity relative to that of the optimal site can be quantified using the PWM [25], the latter two quantities are formally unknown. The formula used by STAP (see Methods) features these two quantities as a mutual product, which is treated as a free parameter in the model. This TF-specific free parameter, henceforth denoted by **γ**, may lead to less of a difference in the contributions of high and moderate affinity sites. That is, at higher **γ** values, as would result from a high effective TF concentration, both high and moderate affinity sites may be fully occupied (saturated occupancy) whereas a stronger bias for high affinity sites will be observed at lower values (Figure 1A). As noted above, we use a cross-validation setting where the parameter is trained on three-quarters of the data and used to predict STAP scores in the left-out quarter, and the process is repeated four times. We examined the role of this parameter in the accuracy of the STAP model by varying it in the broad range 10^−1^ to 10^5^ and recording the CC at each value of **γ**. As shown in Figures 3A–C, the optimal parameter value varies across data sets, between 10^0^ to 10^4^, with a roughly equal split into low, medium and high regions of the allowed range. All experiments reported in the rest of this paper were constrained to use **γ** in the range 10^0^–10^4^. We note that a value of **γ** = 10^0^ indicates that the optimal site has a fractional occupancy of 0.5 at cellular levels of TF concentration, while a value of **γ** = 10^4^ indicates a fractional occupancy of ∼1.

Figures 3A–C also reveal that for any given data set there is a substantial variation in the accuracy of STAP scores as we vary the TF-specific **γ** parameter. For instance, the CC value in the data set “TWI_Fchip_s9” (TWI at stage 9, source: Furlong lab ChIP-chip data) is about 0.25–0.30 at the two extreme values of **γ** (10^−1^ and 10^5^ respectively), but reaches a much higher value of 0.42 at **γ** = 10^2^. This dependence on the **γ** parameter, along with the variability of optimal **γ** across data sets underscores the importance of this parameter in the model. The parameter is analogous to the motif transition probability parameter in HMM-based models used in motif scanning, and our observation highlights the need for data set-specific training of this parameter in order to achieve the most accurate predictions. More generally, we conclude that simply adding the strengths of motif matches in a window is not necessarily the best way to predict TF occupancy in that window.

For a given TF, the **γ** parameter is proportional to the TF's concentration level in the experimental conditions. Therefore, if we have ChIP data on the same TF from two different stages, the optimal **γ** values ought to reflect the relative concentration levels in those stages. The examined collection of data sets included eight such pairs of data sets comprising ChIP data for the same TF from two different developmental stages. We therefore plotted the ratio of the trained **γ** values in the two stages versus the ratio of the TF's expression levels in those stages. We noted (Figure 3D) that the ratios of **γ** values were roughly consistent with ratios of expression levels, in that if one ratio is >1, the other ratio is also greater than or close to 1, and not <1. Expression levels were obtained from RNA-SEQ data from whole-embryos and may therefore be only a crude approximation of cell type-specific protein concentrations. This, and the fact that all ChIP experiments were performed on whole-embryo extracts, are expected to affect the sensitivity of this analysis, and may be the reason why we did not see a more quantitative agreement between the two ratios (i.e., points always close to the diagonal).

### VFL and TRL binding sites frequently influence occupancy through long-range interactions

Our tests so far examined how different aspects of the primary TF, such as its binding specificity and concentration, affect its DNA-binding profile. In the next set of tests, we sought to evaluate the role of TFs other than the primary TF in determining the latter's occupancy. To this end, we used STAP with two motifs – the primary motif and one secondary motif at a time – and allowed cooperative interaction between TF molecules bound at sites within a certain distance, called the “distance threshold”, of each other (Figure 1B). There are now three free parameters: the two **γ** parameters corresponding to the primary and secondary motif, and a parameter representing the interaction energy between bound molecules of the primary and secondary TF. Evaluations performed under a cross-validation scheme ensured that CC values here are comparable to those in the baseline results from Table 1.

In the first set of tests of cooperative effects, we set the distance threshold to be 150 bp, therefore allowing long-range interaction that is similar to the length of DNA in one nucleosome. (We use “long-range” here to contrast with “short-range” interactions inferred from site pairs with ≤30 bp spacing in the next subsection, but note that “long-range” has different connotations in other contexts, e.g., to refer to interactions beyond enhancer boundaries [26], [27].) For each data set, we tested a secondary motif for every TF among the most highly expressed genes in the appropriate developmental stage, based on RNA-SEQ data [21]. We compared the CC of a (primary motif, secondary motif) pair to that from the primary motif (Table 1), and examined all cases where the improvement in CC was ≥0.04 (see Methods). The improvement, henceforth called ΔCC, was subjected to two different assessments of statistical significance. First, we recomputed the ΔCC with one hundred random variants of the secondary motif (see Methods), and asked what fraction of these random ΔCC values were better than the original ΔCC, thus obtaining a “ΔCC p-value”. Second, we utilized the ΔCC values from every tested secondary motif to compute a Z-score (see Methods). This mimics standard outlier detection procedures and designates a ΔCC value as significant if it appears to be an outlier compared to other observed ΔCC values for this data set. This is analogous to a multiple hypothesis correction, since we test over 50 candidate secondary motifs per data set. Additionally, we required that the cooperative interaction model has a greater CC than a model where the secondary motif alone is used by STAP. Thereby, we identified data sets where the combination of the primary and secondary motifs, through cooperative interactions, can describe the primary TF's occupancy better than either motif in isolation.

**Figure 4:**
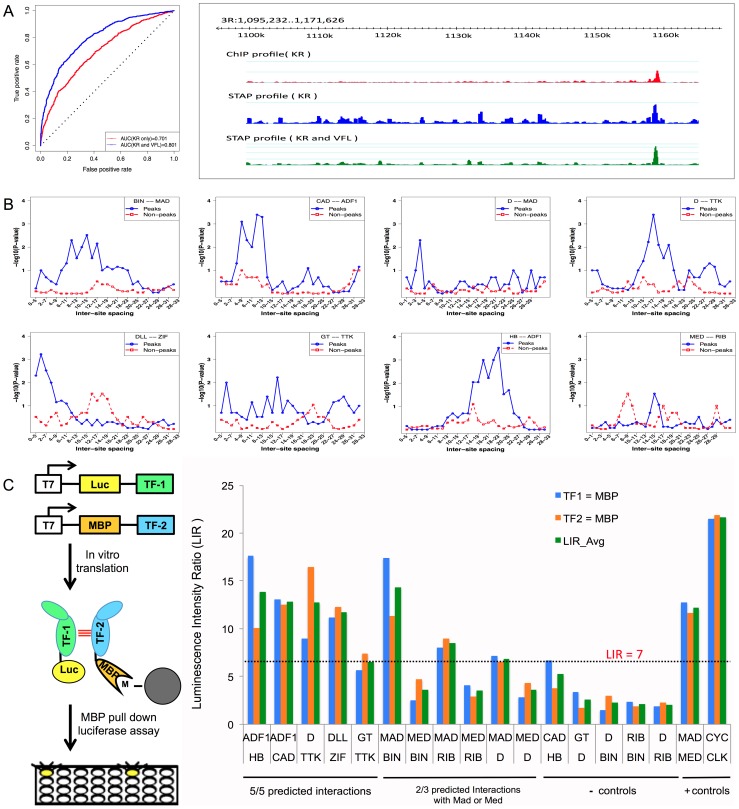
Influence of TF-TF cooperative interactions on TF-DNA occupancy. **A.** ROC curve and Genome Surveyor tracks of the single motif model and best TF-TF interaction model for the data set “KR_Bseq_s5”, where CC(KR) = 0.487, CC(KR+VFL) = 0.774, AUC(KR) = 0.701, and AUC(KR+VFL) = 0.801. **B.** Inter-site spacing bias for a selection of putative TF-TF cooperative interactions. The statistical significance of inter-site spacing bias in the top 250 ChIP peaks (blue) or 250 non-peaks (red) of a data set is measured by the Fisher's Exact Test, for different spacing ranges (x-axis). TF pairs are named in legend (inset) with primary TF appearing first. **C.** Experimental validation of predicted direct TF-TF interactions. (Left) Candidate interacting TF pairs were produced by *in vitro* transcription/translation of renilla luciferase (Luc) and maltose binding protein (MBP) tagged proteins; luciferase activity co-isolated with the MBP-tagged protein was determined. (Right) For each heteromeric pair, interaction was tested in two configurations, with either TF1 or TF2 tagged with MBP. Results for each configuration alone and the average of both experiments are shown. A Luminescence Intensity Ratio (LIR) cutoff of 7 was used for positive interactions. Error bars indicate the Standard Deviation.

This analysis revealed 25 cases of significant improvements (ΔCC ≥0.04, p-value≤0.05 and Z-score ≥3), spread over 18 data sets (Supplementary Table S2). Table 2 tabulates the secondary motif with the most pronounced effect for each of these data sets. We noted that these effects arise mainly from an improved ability to discriminate peaks from non-peaks, and in only 4 (respectively 2) of these 18 cases the cooperativity model improves the CC even within peaks (respectively non-peaks) (Supplementary Table S11, Supplementary Figure S6). Remarkably, for 15 of these 18 data sets, the most influential secondary motif was either VFL (8 cases) or TRL (7 cases). Figure 4A shows an example where the use of VFL as a secondary motif significantly improves the ability to discriminate ChIP peaks from non-peaks. Overall, the VFL motif significantly improves primary TF occupancy predictions for 10 data sets (Supplementary Table S3, Figure S4A), of which 9 were from an early developmental stage (stage 5), and the tenth was from a broader span of developmental stages including stage 5. We noted that VFL is highly expressed in later stages as well and its motif was tested as a secondary motif in the corresponding data sets, but significant influences were not detected in those data sets. VFL has been proposed to play a “pioneer factor” role [28] in early development [29], [30], and its motif has been found to be highly over-represented in so-called “HOT” regions that represent the most accessible regions of the genome [31], [32]. Yanez-Cuna et al. [5] recently showed the VFL motif to be required for DNA-binding by the TF TWI, as well as for regulatory activity of TWI-bound enhancers, and to be enriched in early binding sites of other TFs such as MEF2. Our findings support these strong lines of evidence for an important facilitative role of VFL in determining TF-binding, and explicitly quantify this role for 10 different TF-ChIP data sets.

**Table 2:** Effect of cooperative interactions between pairs of TFs on the accuracy of modeling ChIP data.

Data set	M2	CC(M1)	CC(M2)	CC(M1+M2)	ImprOverM1	ImprOverM2	P-value	Z-score
**BCD_Bseq_s5**	VFL	0.560	0.392	0.603	0.043	0.211	0.00	9.03
**BIN_Fchip_s10_11**	TRL	0.630	0.206	0.682	0.052	0.475	0.02	3.91
**BIN_Fchip_s14**	TRL	0.654	0.216	0.697	0.043	0.481	0.01	7.64
**CAD_Bseq_s5**	VFL	0.178	0.472	0.519	0.341	0.048	0.00	9.99
**D_Bchip_s5**	VFL	0.291	0.310	0.393	0.102	0.083	0.00	19.97
**D_Mseq_s5_11**	HLHM5	0.328	0.373	0.416	0.088	0.043	0.04	4.00
**DA_Bchip_s5**	VFL	0.286	0.266	0.334	0.048	0.068	0.00	11.84
**EN_Mchip_s5_14**	TTK	0.321	−0.159	0.391	0.070	0.232	0.01	23.81
**HB_Bseq_s5**	VFL	0.374	0.326	0.459	0.085	0.133	0.00	6.09
**KR_Bseq_s5**	VFL	0.430	0.388	0.531	0.101	0.143	0.00	21.21
**MED_Bchip_s14**	ZIF	0.255	0.105	0.337	0.082	0.232	0.01	3.33
**RUN_Bchip_s5**	TRL	0.258	0.147	0.328	0.070	0.181	0.00	12.72
**SLP1_Bchip_s5**	VFL	0.214	0.154	0.281	0.067	0.128	0.00	3.47
**TIN_Fchip_s10_11**	TRL	0.392	0.269	0.472	0.080	0.203	0.00	29.57
**TIN_Fchip_s9**	TRL	0.428	0.257	0.480	0.052	0.223	0.02	15.11
**TWI_Bchip_s5**	VFL	0.251	0.280	0.345	0.094	0.065	0.00	15.84
**TWI_Fchip_s10_11**	TRL	0.405	0.305	0.465	0.060	0.160	0.02	10.41
**TWI_Fchip_s9**	TRL	0.420	0.197	0.469	0.049	0.272	0.00	10.55

STAP was used with two motifs – the primary motif (representing the ChIP'ed TF) and a secondary motif (“M2”), and with cooperative DNA-binding included in the model. Cooperative interaction between two TF sites was included in the model only if the two sites are within a pre-defined “Distance Threshold” set to 150 bp in this set of experiments. The correlation coefficient between STAP scores from this model (CC(M1+M2)) was compared to the CC when using only the primary motif (CC(M1)) or when using only the secondary motif (CC(M2)) in STAP. The respective improvements are noted as “ImprOverM1” and “ImprOverM2” respectively. The column “P-value” shows an empirically calculated p-value for the improvement, comparing the observed improvements to that expected from 100 shuffled versions of the secondary motif. The column “ImprOverM2” is the difference of CC(M1+M2) and the absolute value of CC(M2). The last column (“Z-score”) compares the observed improvement (ImprOverM1) to that obtained using other real motifs, corresponding to TFs expressed highly in that developmental stage, as the secondary motif. Shown here is only the single strongest secondary motif influence on each data set, if its P-value is ≤0.05 and Z-score is ≥3. The complete list of significant effects is in Supplementary Materials (Table S2). Note that all results are from cross-validation and thus account for the additional parameters in the two motifs model compared to the one motif model.

We found the TRL motif to influence the binding levels of primary TFs in eight data sets overall, of which six are from the later developmental stages 9–14 (Supplementary Table S4). As discussed in [33]–[35], TRL plays an important role in regulating the chromatin structure and packaging large segments of the chromosome into active (euchromatic) or inactive (heterochromatic) domains. The TRL motif was also prominent among sequence signatures of context-specific TF-DNA binding reported in [5], although this previous study did not explicitly quantify its facilitative effect on various primary TFs. It is interesting to note that TRL is the TF with the highest baseline CC (Table 1), reflecting the possibility that TRL-DNA binding is largely dependent on the TF itself and does not require facilitative effects of secondary TFs. This is consistent with speculation that TRL is a “pioneer factor” [28], [36].

In these initial tests, STAP was configured to allow interaction between primary and secondary motif as long as their bound sites were within 150 bps. We next asked if the promiscuous effects of VFL and TRL could be observed when reducing this distance threshold to 30 bps, which would suggest that short-range mechanisms of interaction might be involved. We found that in most cases the effects of these two motifs were not significant at the shorter distance range (Supplementary Tables S17, S18), and in the four cases where significant effects were detected at this range, the magnitude of the effect was lower than that at 150 bp range. A possible interpretation of this finding, especially in light of available knowledge about these two proteins, is that they act as chromatin remodelers over relatively long scales (150 bp or greater) and facilitate TF binding by making binding sites of the primary TF more accessible. (We revisit this point in a later section, by directly examining accessibility data.) Notably, the data sets for VFL and TRL themselves did not reveal any secondary motifs with significant effects, once again supporting a possible pioneer factor role for these two TFs.

### Short-range interactions with secondary TF sites influence primary TF occupancy and can predict physical interactions between TFs

While VFL and TRL clearly show the most frequent effects on TF binding, a number of other influential secondary TF motifs were also revealed by our analysis; these are shown in Table 3. For each of these cases we report the ΔCC values at both distance thresholds (30 bp and 150 bp). Of particular interest were the (primary motif, secondary motif) pairs where the ΔCC was significant only at the 30 bp threshold, since this may reflect direct interactions. (These significant short-range interactions were reflected in a better ability to discriminate peaks from non-peaks rather than an improved ranking of the peaks; see Supplementary Table S12 and Supplementary Figure S7.) A case in point is the data set HB_Bchip_s9, for the TF hunchback (HB), where the secondary motif Adh transcription factor 1 (ADF1) improves the baseline CC of 0.204 to 0.303 when modeling heterotypic cooperativity at distance threshold 30 bp. The ΔCC of 0.099 is highly significant (empirical P-value = 0, i.e., no shuffled motif yielded better ΔCC), while that at the 150 bp threshold does not meet our significance criteria. A similar effect was observed for the ADF1 motif on HB ChIP data in stage 5 embryos. We hypothesized that this is evidence for direct physical interaction between HB and ADF1 resulting in modulation of HB binding levels. We searched for sequence signatures of such a hypothesized interaction in the relative spacing of HB and ADF1 binding sites. Examination of the 250 highest ChIP peaks in the data set showed a statistically significant bias (P-value 3E-4, see Methods) for spacing in the range 18–23 bps (Figure 4B). A similar test on 250 non-peaks from the data set showed no bias for this range or any other. This analysis suggests that proximally located binding sites of HB and ADF1 result in increased HB occupancy in ChIP peaks. We examined other data sets where the ΔCC was significant, and found similar evidence of biased inter-site spacing in ChIP peaks (Figure 4B), supporting the hypothesis that direct cooperative interactions may be a key factor in determining TF binding profiles in these cases. In some cases, e.g., the pair (D, TTK) and (GT, TTK), we noticed more than one preferred spacing range, separated by 11 bp, as might be expected due to proper phasing requirements between physically interacting TFs [37]. We also tested for biased inter-site spacing between the TFs Distal-less (DLL) and Zif Zinc-finger protein (ZIF, also called CG10267) (Figure 4B), because the ΔCC was found to be significant for this pair (empirical p-value 0), although the z-score of this ΔCC was 2.435, slightly below our chosen threshold of 3.0.

**Table 3:** Effects of cooperative DNA binding by the primary TF and a secondary TF (“M2”), that were significant at distance threshold either 30 bp or 150 bp.

Data set	M2	CC(M1)	CC(M1+M2); 30 bp	ImprOverM1; 30 bp	CC(M1+M2); 150 bp	ImprOverM1; 150 bp	Tag
**BIN_Fchip_s10_11**	ADF1	0.630	-	-	0.676	0.047	*
**BIN_Fchip_s10_11**	BRK	0.630	-	-	0.682	0.052	
**BIN_Fchip_s10_11**	MAD	0.630	-	-	0.686	0.056	
**CAD_Bseq_s5**	ADF1	0.178	-	-	0.391	0.212	
**CAD_Bseq_s5**	CG7928	0.178	0.254	**0.076**	0.257	0.079	
**CAD_Bseq_s5**	DL	0.178	0.268	**0.089**	0.268	0.090	
**CAD_Bseq_s5**	TTK	0.178	0.286	0.107	0.328	0.150	
**D_Mseq_s5_11**	ADF1	0.328	0.380	**0.052**	-	-	*
**D_Mseq_s5_11**	HLHM5	0.328	-	-	0.416	0.088	*
**D_Mseq_s5_11**	MAD	0.328	0.380	**0.052**	-	-	*
**D_Mseq_s5_11**	TTK	0.328	-	-	0.400	0.072	*
**D_Mseq_s5_11**	ESPL	0.328	0.373	0.045	0.415	0.087	
**D_Mseq_s5_11**	HLHMBETA	0.328	0.378	0.050	0.394	0.066	
**EN_Mchip_s5_14**	TTK	0.321	0.388	**0.067**	0.391	0.070	*
**EVE_Mseq_s14**	ARA	0.174	0.291	**0.116**	0.268	0.094	
**EVE_Mseq_s14**	CAUP	0.174	0.291	**0.116**	0.275	0.100	
**GT_Bseq_s5**	TTK	0.241	-	-	0.288	0.047	
**HB_Bchip_s9**	ADF1	0.204	0.303	**0.099**	-	-	*
**HB_Bchip_s9**	WOR	0.204	0.312	**0.107**	0.308	0.103	
**HB_Bseq_s5**	ADF1	0.374	0.427	**0.053**	-	-	
**MED_Bchip_s10**	PNR	0.184	-	-	0.226	0.042	
**MED_Bchip_s10**	SIX4	0.184	0.224	**0.040**	0.227	0.043	
**MED_Bchip_s14**	ZIF	0.255	-	-	0.337	0.082	*
**MED_Bchip_s14**	RIB	0.255	0.341	**0.086**	-	-	*
**MED_Bchip_s14**	TTK	0.255	-	-	0.331	0.075	*
**MED_Bchip_s5**	DEAF1	0.102	0.153	**0.051**	-	-	*
**MED_Bchip_s5**	HB	0.102	0.165	**0.063**	-	-	*
**RUN_Bchip_s5**	TTK	0.258	-	-	0.304	0.046	
**SLP1_Bchip_s5**	ADF1	0.214	0.263	0.049	0.290	0.076	
**SLP1_Bchip_s5**	TTK-PF	0.214	-	-	0.275	0.061	
**TIN_Fchip_s10_11**	Z	0.392	-	-	0.438	0.046	
**TLL_Bchip_s5**	DL	0.159	-	-	0.206	0.047	
**TWI_Bchip_s5**	DL	0.251	-	-	0.319	0.068	
**TWI_Fchip_s10_11**	Z	0.405	-	-	0.445	0.040	
**TWI_Fchip_s9**	TIN	0.420	-	-	0.465	0.045	

Only cases involving secondary motifs other than “VFL” and “TRL”, and where P-value≤0.05 and Z-score ≥3 are shown here. Cases where the improvement at distance threshold 30 bp was similar to or better than that at 150 bp are highlighted in bold. Dashes indicate that the secondary motif was not observed to show significant improvement at the specific distance threshold. This table presents results from testing all secondary motifs whose TFs were in the top 25% most highly expressed TF genes (out of the ∼300 TF genes with motifs) in the appropriate developmental stage. This is contrast to other tables (2, 3, 4, 6), where the tested secondary motifs correspond to TFs that are in the top 10–15% most highly expressed TF genes for the stage. In this table, the rows marked with asterisks correspond to secondary TFs in the top 10–15% most highly expressed TF genes.

For each of the predicted heterotypic interactions shown in Figure 4B, we assayed for direct physical interactions between the TFs using a modification of the LUMIER method [38], [39]. In these experiments, one partner is expressed as a fusion to Maltose Binding Protein (MBP) and the other partner as a fusion to luciferase (luc). To avoid possible bridging interaction by other eukaryotic proteins, the proteins were expressed using a purified prokaryotic *in vitro* expression system and then combined for analysis. MBP-tagged proteins were isolated using amylose beads and the luciferase activity retained on the beads (via primary TF-secondary TF interaction), relative to a negative control with unfused luc, was used to calculate a luminescence intensity ratio (LIR, see Methods). A value of seven or greater was selected as a cutoff for positive interactions. This threshold is based on a set of positive and negative control interactions among bHLH protein dimers examined using this assay (HNP and MHB, unpublished) as well as additional negative controls using luc fused to the TF CLK or MBP without a fusion partner (Supplementary Figure S12). This threshold is more than twice as stringent than those used in previous studies examining protein interactions in cell culture [38], [39] and consequently may exclude some weaker interactions, including some that may only be significant in the context of cooperative binding to DNA. Each predicted interaction pair was examined in both configurations (e.g., the primary TF was fused to MBP in one experiment and to luc in the second). In addition, since Mothers against dpp (MAD) and Medea (MED) are known to bind DNA as a heteromeric complex [40]–[43], it is possible that any interaction computationally identified for one of these proteins is the result of an interaction with the other one. In our *in vitro* experiments, only direct physical interactions between two proteins are tested. Therefore, each of the predicted interactions for either MAD or MED was also tested with the other.

For five of the eight tested pairs (i.e., those not involving MAD or MED), a clear *in vitro* interaction was observed in both configurations (Figure 4C, Supplementary Table S9). For the two predicted interactions involving MAD, one of the two configurations gave a signal while the other was just below our selected cutoff. In one additional case, no physical interaction was observed between ribbon (RIB) and MED, but RIB was observed to interact with the MED binding partner, MAD. None of our tested negative controls was near the threshold and the interaction signal for most of the tested pairs was similar to two, well-established positive control interactions for this set of proteins, a MAD-MED heterodimer and a homodimer of giant (GT), which is a member of the bZIP family of TFs that bind DNA as homodimers [44]. Thus, all of the tested predictions are supported by a moderate to strong *in vitro* interaction, demonstrating that at least some of the short range cooperative interactions identified by our computational model reflect actual physical interactions that were previously unrecognized in large scale protein-protein interaction screens.

**Figure 5:**
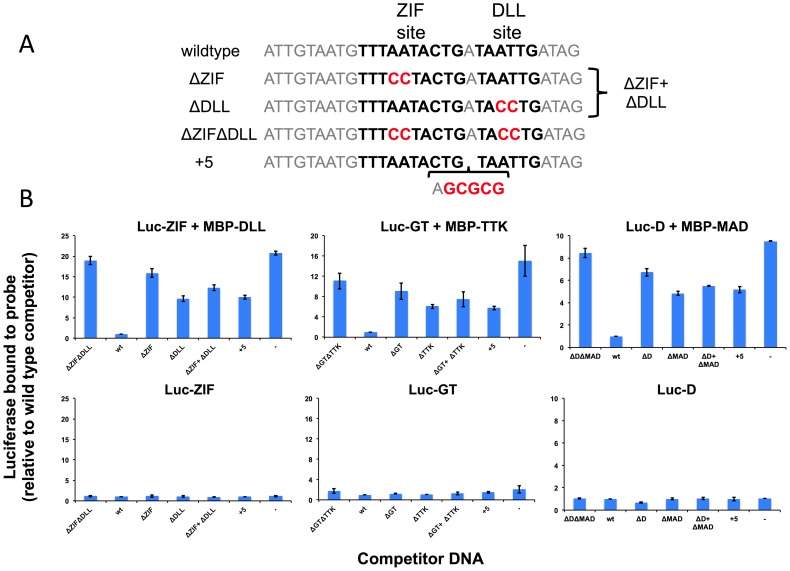
Experimental validation of predicted cooperative DNA binding by three TF pairs, ZIF with DLL, GT with TTK and D with MAD. Relative recovery of luciferase-tagged TF with a biotinylated target DNA sequence is measured in the presence of one or both TFs and various unlabeled competitor sequences. (**A**) Examples of wild type and mutant competitor sequences are shown for the analysis of ZIF with DLL. The sequences for all competitor sequences are shown in Table S16. The wild type sequence has a strong predicted TF binding sites, shown in bold type, for the luc-tagged TF (ZIF, GT, D) and for the hypothesized interacting TF (DLL, TTK, MAD respectively). As controls, competitor sequences are used where either one (ΔZIF or ΔDLL) or both (ΔZIFΔDLL) TF binding sites are disrupted or the spacing between sites has been increased by 5 bp (+5). An additional competition experiment uses two competitor DNAs (ΔZIF + ΔDLL), each of which is at the same concentration as the single competitor DNAs in the other samples. Altered or inserted nucleotides are shown in red. Genomic sequences flanking the binding sites are in grey. (**B**) The luciferase activity recovered bound to the biotinylated wild type probe was measured in the presence of different competitors listed on the X-axis. A dash is used to indicate no added competitor DNA. Luciferase measurements are reported relative to a sample using the wild type sequence as a competitor (Y-axis). In the upper panels, recovery of the luciferase-tagged protein is measured in the presence of the hypothesized interacting TF present as an MBP tagged protein. In the presence of the secondary TF, wild type sequences compete better for binding than sequences in which the sequence of or spacing between predicted binding sites is disrupted. In the lower panels, recovery is shown in the absence of the second protein. For all three primary TFs, little activity is recovered in the absence of the secondary TF, regardless of the competitor DNA.

The physical interaction of TFs suggests that they may use cooperativity to increase binding of the primary TF to DNA sites with properly spaced binding sites for both TFs [45]. We tested this prediction for three of the above TF pairs using a variation of a previously described microwell assay [46] (Figure 5). The primary TF fused to luciferase and a secondary TF fused to MBP are used in an *in vitro* pull down assay with biotinylated dsDNA oligos containing a sequence from the ChIP peaks that contains binding sites for both TFs. The TFs are mixed with the biotinylated target site and an excess of unlabeled wild type or mutant competitor DNA. The competitor sequences used to examine cooperative DNA binding of ZIF and DLL are shown in Figure 5A and all sequences are shown in Supplemental Table S16. Streptavidin-mediated recovery of luc-TF/biotin-DNA complexes in the presence of excess wild type competitor (wt) indicates the background signal. In experiments with both TFs present (Figure 5B), the recovery of the luciferase-tagged primary TF in the presence of a competitor with mutations in both TF binding sites (e.g. ΔZIFΔDLL) increased 8–18 fold over the background in the presence of wt competitor (Figure 5B, upper panels). In contrast, little increase was observed when this experiment was repeated without the secondary TF (Figure 5B, lower panels), indicating that the secondary TF facilitates binding of the primary TF to these sites. The specificity of this interaction was confirmed by testing mutant competitor DNAs that disrupt the individual TF binding sites (e.g., ΔZIF or ΔDLL) or that increase the intersite distance by five base pairs (e.g., “+5”). Each of these alterations in the DNA sequence results in reduced competition by the mutant DNA competitor relative to wild type and increased recovery of the primary TF (Figure 5B). Furthermore, reduced competition is observed even when adding two competitors with mutations in one or the other individual TF binding site and each present at the same concentration as the wild type control; thus, high affinity binding requires the two TF binding sites to be present on the same DNA molecule with the proper spacing. These results indicate that the physical interactions detected for each of these pairs mediate cooperative DNA binding to an endogenous sequence from one of the top ChIP peaks.

In light of the possibility that the influence of short range cooperative interactions may be more pronounced when the interacting TFs are at relatively modest concentration levels, we extended the tests reported in Table 3 to include all candidate secondary TFs with expression in the top 50%. The results, shown in Supplementary Table S14, reveal that for several data sets stronger influences are detectable when allowing lower expression levels of the secondary TF. On the one hand, this means that the list of interactions identified in Table 3 is likely incomplete. On the other hand, the list shown in Table S14 must be interpreted with caution since testing more candidate secondary TFs may lead to spurious interactions being reported due to similarity of motifs between two candidates.

### Competitive binding by secondary TFs frequently influences occupancy

**Figure 6:**
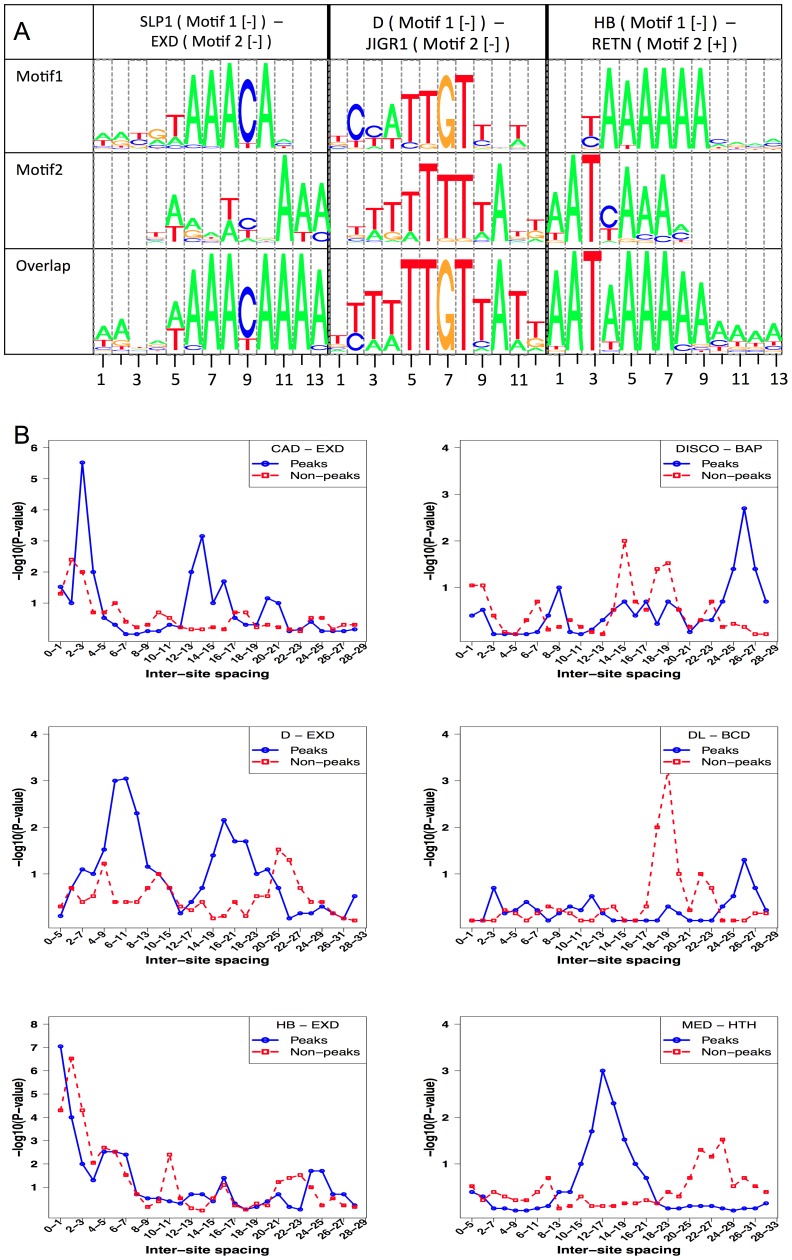
Competitive bindings and antagonistic bindings: another mechanisms deciding TF-DNA occupancy. **A.** Overlapping sites for (primary motif, secondary motif) pairs where modeling competitive binding leads to significantly better prediction of ChIP scores. Shown here is the pattern of overlap between sites of SLP1 and EXD, D and JIGR1 and between HB and RETN. In each case, the sequences examined were those with high STAP score for the primary motif but low ChIP score. In each panel, the top two motif logos correspond to the primary and secondary TF respectively and the bottom logo represents the overlapping sites. **B.** Inter-site spacing bias analysis for six TF pairs that show significant evidence of antagonistic binding. TF pairs are named in legend (inset) with primary TF appearing first.

Cooperative interactions are not the only manner in which one TF's binding may influence another's. Two TFs competing for overlapping binding sites can modulate each other's binding levels at the location [47]. Our next set of tests searched for evidence of this phenomenon in ChIP data sets. We used a two motif STAP model with no interaction terms, and compared the cross-validation CC from this model to the baseline CC of Table 1. The only way in which a secondary TF site can influence the binding prediction for the primary TF in the two-motif model is if their sites overlap (Figure 1B). The results, shown in Table 4, comprise 17 cases of significant ΔCC over the baseline model (ΔCC ≥0.04, P-value≤0.05, Z-score ≥3). In at least 10 of these cases, the secondary motif's presence is strongly anti-correlated with the primary TF's ChIP score, i.e., the competing motif is more frequent in non-peaks or in lower ranking peaks than in strong peaks. This may imply that the strong peaks exhibit selection against sites of the secondary TF competing with the primary motif. Figure 6A shows three examples of the pattern of overlap between sites of a primary TF and a secondary TF, observed in sequences with high STAP scores and low ChIP scores. We noted that in all of these cases, the overlapping sites tended to be suboptimal matches to either motif.

**Table 4:** Effect of competitive interactions between pairs of TFs on the accuracy of modeling ChIP data.

Data set	M2	CC(M1)	CC(M2)	CC(M1+M2)	ImprOverM1	ImprOverM2	P-value	Z-score
**D_Mseq_s5_11**	JIGR1	0.328	−0.277	0.368	0.040	0.091	0.02	25.90
**DL_Bchip_s5**	BCD	0.062	−0.116	0.193	0.131	0.077	0.01	47.80
**EN_Mchip_s5_14**	HTH	0.321	−0.187	0.397	0.076	0.210	0.00	17.09
**EN_Mchip_s5_14**	SNA	0.321	−0.129	0.362	0.041	0.233	0.03	9.23
**EVE_Mseq_s14**	HTH	0.174	−0.195	0.245	0.071	0.050	0.03	10.45
**HB_Bchip_s9**	RETN	0.204	−0.137	0.282	0.078	0.145	0.03	66.15
**HB_Bchip_s9**	EXD	0.204	−0.019	0.276	0.072	0.258	0.03	61.15
**HB_Bseq_s5**	EXD	0.374	−0.007	0.436	0.062	0.429	0.00	47.53
**HB_Bseq_s5**	RETN	0.374	0.023	0.420	0.046	0.398	0.02	35.84
**MED_Bchip_s14**	HTH	0.255	−0.178	0.360	0.105	0.182	0.00	16.81
**MED_Bchip_s14**	TTK-PF	0.255	−0.277	0.329	0.074	0.052	0.05	11.75
**MED_Bchip_s14**	SNA	0.255	−0.067	0.313	0.058	0.246	0.02	9.16
**MED_Bchip_s14**	HR78	0.255	−0.092	0.304	0.049	0.212	0.04	7.79
**MED_Bchip_s14**	HR4	0.255	−0.130	0.300	0.045	0.170	0.00	7.14
**MED_Bchip_s14**	HR46	0.255	−0.143	0.300	0.045	0.157	0.02	7.14
**SLP1_Bchip_s5**	CAD	0.214	−0.036	0.262	0.048	0.226	0.01	27.85
**SLP1_Bchip_s5**	EXD	0.214	0.001	0.254	0.040	0.253	0.02	23.11

STAP was used with two motifs – the primary motif and a secondary motif (“Motif 2”), but with no interaction between binding sites considered. Thus the only influence of Motif 2 on predicted occupancy of the primary TF is due to overlapping sites. Shown are all cases in which both improvements were more than 0.04, P-value≤0.05 and Z-score ≥3. The column “ImprOverM2” is the difference between CC(M1+M2) and the absolute value of CC(M2).

Two different data sets involving HB, one from stage 5 and the other from stage 9, were influenced by overlapping sites of the RETN motif (Table 4). RETN is a well-known repressor that acts through competitive binding when inhibiting activation by the TF engrailed (EN) [48]. Two other secondary motifs that seem to influence multiple data sets are EXD and HTH. Both of these homeodomain proteins play prominent roles during development as cofactors in repressor complexes with both Hox and other homeodomain proteins. Interestingly, in all three cases where EXD influences binding, there is no correlation between EXD sites and the primary TF occupancy, while in all three cases where HTH exerts an influence, there is a strong negative correlation (∼−0.18) between HTH motif presence and primary TF binding (see Discussion).

### Antagonistic influence of a secondary motif can manifest even with non-overlapping sites

The next set of tests was directed at detecting evidence of antagonistic binding at non-overlapping sites. A possible mechanism for such a phenomenon is that of the secondary TF upon binding rendering the local DNA inaccessible, e.g., through recruitment of HDACs [49], as is speculated to be the case with some short-range repressors in *Drosophila* [50]. We used a two-motif STAP model with a TF-TF interaction term that is fit on training data, and compared the resulting CC to that from the primary motif alone (Table 1). This interaction term was constrained to be <1, corresponding to an unfavorable energy of interaction in the underlying thermodynamics model (Figure 1B). Note that this model incorporates both competitive binding and antagonistic influence from non-overlapping sites. Comparing the CC achieved by this model at either the 30 bp or the 150 bp distance threshold to the baseline (Table 5, Supplementary Figure S4B), we found 35 cases of significant improvements (ΔCC ≥0.04, P-value<0.05, Z-score ≥3). These included 6 data sets influenced by the EXD motif, 4 data sets by the HTH and RETN motifs, and 3 data sets by the JIGR1 motif. We noted that these four motifs were also observed to influence binding through competitive binding to overlapping sites (Table 4) above. However, in such cases where a secondary motif had significant effect on binding levels both in the competitive binding mode as well as the antagonistic binding mode, the magnitude of the effect was always stronger in the latter mode. The strongest case of antagonistic influence at the 30 bp distance threshold was estimated for the data set CAD_Bseq_s5, for the TF caudal (CAD), where the RETN motif improves the CC from 0.178 to 0.401. On the other hand, the strongest influence at the 150 bp threshold was by the EXD motif, also on the CAD_Bseq_s5 data set, where the baseline CC of 0.178 improved to 0.412, and this effect was exclusive to the 150 bp range. In fact, a large majority of the antagonistic binding influences were significant exclusively at either the short (30 bp) or the long (150 bp) range (Table 5). This may suggest that the underlying mechanisms of short and long-range antagonistic influences are different, although we did not observe any motif-specific preferences for one range versus the other.

**Table 5:** Effect of antagonistic interactions between pairs of TFs on the accuracy of modeling ChIP data.

Data set	M2	CC(M1)	CC(M1+M2); 30 bp	ImprOverM1; 30 bp	CC(M1+M2); 150 bp	ImprOverM1; 150 bp
**BCD_Bseq_s5**	EXD	0.56	-	-	0.606	0.046
**CAD_Bseq_s5**	EXD	0.178	-	-	0.412	0.234
**CAD_Bseq_s5**	RETN	0.178	0.401	0.223	-	-
**DISCO_Mseq_s5_11**	BAP	0.22	0.273	0.053	-	-
**DISCO_Mseq_s5_11**	HB	0.22	-	-	0.378	0.158
**DISCO_Mseq_s5_11**	MES2	0.22	0.263	0.043	-	-
**DLL_Mchip_s5_14**	HTH	0.145	0.286	0.141	-	-
**DLL_Mchip_s5_14**	TTK-PF	0.145	0.281	0.136	-	-
**DL_Bchip_s5**	BCD	0.062	0.254	0.192	0.234	0.172
**DL_Bchip_s5**	EXD	0.062	0.217	0.155	-	-
**D_Mchip_s5_14**	TTK	0.065	-	-	0.173	0.108
**D_Mseq_s5_11**	CAD	0.328	0.421	0.093	-	-
**D_Mseq_s5_11**	EXD	0.328	-	-	0.438	0.11
**D_Mseq_s5_11**	HB	0.328	-	-	0.423	0.095
**D_Mseq_s5_11**	JIGR1	0.328	0.405	0.077	0.485	0.157
**D_Mseq_s5_11**	RETN	0.328	-	-	0.445	0.117
**EN_Mchip_s5_14**	HTH	0.321	0.38	0.059	0.401	0.08
**EVE_Mseq_s14**	HR78	0.174	0.261	0.087	-	-
**EVE_Mseq_s14**	HTH	0.174	0.275	0.101	0.321	0.147
**EVE_Mseq_s14**	TTK	0.174	0.251	0.077	0.223	0.049
**HB_Bchip_s9**	RETN	0.204	0.315	0.111	-	-
**HB_Bseq_s5**	EXD	0.374	0.463	0.089	0.416	0.042
**HB_Bseq_s5**	RETN	0.374	0.444	0.07	-	-
**HKB_Mseq_s14**	JIGR1	0.551	0.6	0.049	-	-
**KNI_Bchip_s5**	BAP	0.102	-	-	0.162	0.06
**MED_Bchip_s14**	HR4	0.255	0.308	0.053	-	-
**MED_Bchip_s14**	HR46	0.255	-	-	0.342	0.087
**MED_Bchip_s14**	HR78	0.255	-	-	0.348	0.093
**MED_Bchip_s14**	HTH	0.255	0.381	0.126	0.373	0.118
**SENS_Mchip_s9_11**	EXD	0.163	-	-	0.235	0.072
**SENS_Mchip_s9_11**	PDM2	0.163	-	-	0.209	0.046
**SLP1_Bchip_s5**	CAD	0.214	0.271	0.057	-	-
**SLP1_Bchip_s5**	JIGR1	0.214	0.275	0.061	-	-
**UBX_Mchip_s9_11**	KR	0.279	-	-	0.324	0.045
**UBX_Mchip_s9_11**	TTK-PF	0.279	-	-	0.345	0.066

STAP was used with two motifs – the primary motif and a secondary motif (“Motif 2”), and with antagonistic DNA-binding included in the model. Interaction between two TF sites was included in the model only if the two sites are within a pre-defined “Distance Threshold” (set to 150 bp and 30 bp in two sets of experiments). Shown are all cases in which both improvements were more than 0.04, P-value≤0.05 and Z-score ≥3.

We searched for inter-site spacing biases that might provide additional insights into the significant antagonistic influences identified above. It was commonly the case that ChIP peaks had a significant bias towards specific spacing values while non-peaks tended to avoid that range (Figure 6B, e.g., D-EXD). Interestingly, though less commonly, such spacing biases were also observed in non-peaks (Figure 6B, Supplementary Tables S5, S19). Even when examining antagonistic influences of the same secondary TF, e.g., HTH, we found some data sets where the spacing bias was exclusive to ChIP peaks and others where the bias was present in non-peaks.

Separate examination of peaks and non-peaks for effects of antagonistic influence revealed that such effects are manifested in a better discrimination of peaks versus non-peaks as well as a better modeling of ChIP scores with peaks alone or, more commonly, within non-peaks (Supplementary Table S13 and Supplementary Figure S8).

### DNA accessibility data provides clues about mechanisms of secondary TF action

Recent work [6], [7] has shown that DNA accessibility data, which reflects nucleosome positioning and other chromatin-related effects, has a very strong correlation with TF occupancy, and when used in conjunction with the primary TF's motif can lead to highly accurate predictions of occupancy. This has been demonstrated in the context of five TFs in *Drosophila* (data from whole embryo) and six TFs in human (data from two cell lines). These prior results motivated us to examine the same hypothesis for the much larger collection of TF-ChIP data sets studied here. In all of our tests in this section we used DNaseI hypersensitivity data from [19]. In the first tests, we used a high threshold (90^th^ percentile) on developmental stage-specific accessibility to designate “accessible regions”, predicted zero occupancy in inaccessible regions, and used STAP and the primary motif to predict occupancy in accessible regions. Accessibility-filtered STAP scores computed in this manner correlated very highly with ChIP data (Supplementary Table S6), and led to substantial improvements upon the baseline results of Table 1, for 38 of the 45 data sets. This confirms that the observations made by Kaplan et al. and Pique-Regi et al. are manifest over a larger dataset.

The test above showed that motif and accessibility information together provide highly accurate predictions of ChIP scores. A natural question that arises then is: how strong is the influence of the primary TF's motif in determining its occupancy, beyond the influence of accessibility? To answer this question we computed the “semi-partial correlation coefficient” (SPCC) between ChIP and STAP scores, which subtracts or “partials out” the contribution of accessibility information. Technically, this amounts to first predicting ChIP scores using accessibility alone, and then correlating the residual ChIP scores with STAP scores (see Methods). We found that for the majority of data sets the SPCC values (Table 1, column SPCC(m1)) were comparable to the baseline CC values, demonstrating that, as expected, the primary motif plays a major role in shaping TF binding profiles. For ten data sets, SPCC was better than baseline CC, most notably for the data set TIN_Fchip_s9 where the primary motif's correlation improves from 0.428 to 0.507 upon partialing out accessibility. In these cases, factoring out the accessibility effects better reveals the expected relationship between primary motif presence in the sequence and occupancy. In contrast, five data sets showed a dramatically lower SPCC than CC (Table 1); these were related to the TFs VFL, TRL and MED. This is consistent with hypothesis emerging in this work (also see next paragraph) and in recent literature that VFL and TRL have direct influence on accessibility patterns, and partialing out the correlation with accessibility results in much reduced correlation between primary motif and TF occupancy. The third of the trio of TFs identified here, MED, is also believed to direct the co-factor CBP to the genome [51] and thus influence accessibility profiles. The SPCC was lower than CC also for TWI, D and SLP1, though not as dramatically. Sandmann et al. [52] have previously found TWI to bind to a large number of mesodermal enhancers and speculated that its role may be to facilitate chromatin remodeling. D is a SOX domain protein and there has been suggestion that this family of TFs may function as chromatin remodelers [53]. Interestingly, independent evidence in support of the accessibility-mediated effect of VFL, TRL, TWI, SLP1 and MED emerged when we repeated the evaluation of the single motif STAP model (baseline, Table 1) on data sets composed of the top 1000 ChIP peaks and 1000 random non-peaks selected from ChIP peaks of other TFs (Supplementary Table S15 and Supplementary Figure S9). We found the CC on these data sets to be conspicuously below that on the default data sets where the non-peaks were random genomic segments. This implies that the primary motif in these cases is better able to discriminate peaks of the primary TF from random non-peaks than from other accessible regions (peaks of different TFs). This in turn suggests that the motifs of VFL, TRL, TWI, SLP1 and MED may be common features of many ChIP peaks that discriminate them from random non-coding sequences irrespective of the bound TFs.

**Figure 7:**
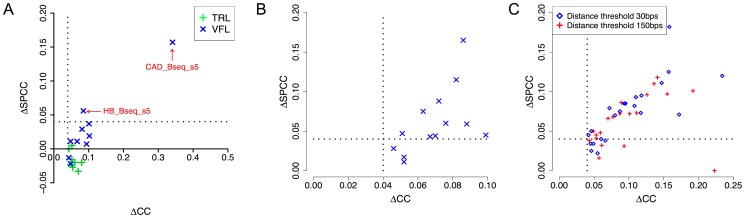
DNA accessibility data provides clues about mechanisms of secondary TF action. **A.** Correlation coefficient between ChIP scores and STAP predictions, before and after “partialing out” the effect of accessibility scores (**Δ**CC and **Δ**SPCC respectively). Shown here are the effects of VFL and TRL motifs, in cooperative binding mode. Only cases where **Δ**CC was significant are shown. Dotted lines mark a ΔCC (or ΔSPCC) value of 0.04. **B.** All cases of significant cooperative influence of secondary motifs other than VFL, TRL, examined before (ΔCC) and after (ΔSPCC) “partialing out” the effect of accessibility scores. Only cases where **Δ**CC was significant and the secondary TF gene was in the top 10–15% of expressed genes are shown. **C.** All cases of antagonistic influence by secondary motifs, examined before and after “partialing out” the effect of accessibility scores.

Our next tests examined the effect of cooperative binding with secondary TFs in the light of accessibility information. Recall that the VFL and TRL motifs had emerged as the most promiscuous influences in our tests above (Table 2), and that their influence was noted as being predominantly long-range (Supplementary Tables S3, S4), leading us to speculate that they may be mediated through modulation of local accessibility. We therefore asked if the improvements in CC due to either of these motifs are observed after removing the effects of accessibility information. We computed SPCC values of the cooperative interaction model after partialing out accessibility (Figure 7A), similar to that described in the previous paragraph. We found that the effects of TRL disappear in all 8 data sets where it had been significant before considering accessibility, adding evidence in favor of our hypothesis that TRL's influence is mediated by accessibility. In contrast, VFL was found to exhibit a more diverse behavior: in 7 of 10 data sets its effects vanished after considering accessibility, while in 2 data sets (CAD_Bseq_s5 and HB_Bseq_s5), a pronounced influence (ΔSPCC ≥0.04) remained even after partialing out accessibility (Supplementary Table S7). These two data sets also showed evidence of an inter-site spacing bias between VFL and the primary motif (Supplementary Figure S3). These findings suggest that VFL's influence on TF binding may involve distinct mechanisms, including not only a general effect on local accessibility, but also more TF-specific mechanisms potentially involving direct interactions with the primary TF.

We repeated the above analysis on data sets where secondary motifs other than VFL and TRL had led to significant improvements in CC through a cooperative binding model (Table 3). The results, shown in Table 6 and Figure 7B, reveal that in most cases the influence of the secondary motif is pronounced even after partialing out accessibility information. This suggests that most of these secondary TFs operate through primary TF-specific interactions rather than by only influencing accessibility. Similar results were obtained when examining the cases of antagonistic influence by secondary motifs (Figure 7C).

**Table 6:** Cases of significant cooperative influence of secondary motifs other than VFL, TRL, re-examined after “partialing out” the effect of accessibility scores.

Data set	M2	Distance Threshold	SPCC(M1)	SPCC(M1+M2)	ΔSPCC
**BIN_Fchip_s10_11**	ADF1	150	0.646	0.675	0.028
**D_Mseq_s5_11**	ADF1	30	0.364	0.375	0.011
**D_Mseq_s5_11**	HLHM5	150	0.364	0.423	**0.059**
**D_Mseq_s5_11**	MAD	30	0.364	0.382	0.017
**D_Mseq_s5_11**	TTK	150	0.364	0.453	**0.088**
**EN_Mchip_s5_14**	TTK	30	0.359	0.401	**0.043**
**EN_Mchip_s5_14**	TTK	150	0.359	0.403	**0.044**
**HB_Bchip_s9**	ADF1	30	0.284	0.328	**0.045**
**MED_Bchip_s14**	CG10267	150	0.102	0.217	**0.115**
**MED_Bchip_s14**	RIB	30	0.102	0.267	**0.165**
**MED_Bchip_s14**	TTK	150	0.102	0.162	**0.060**
**MED_Bchip_s5**	DEAF1	30	−0.046	0.000	**0.047**
**MED_Bchip_s5**	HB	30	−0.046	0.029	**0.075**

This list only shows secondary motifs corresponding to top 10–15% most highly expressed TF genes (marked with asterisks in Table 3). SPCC(M1) and SPCC(M1+M2) denote CC of a single-motif STAP model and a two-motif STAP model with cooperativity, both after partialing out accessibility. Bold font in the last column refers to cases where ΔSPCC is ≥0.04.

## Discussion

We studied mechanistic determinants of TF-DNA binding by computationally modeling genomic occupancy from over 40 ChIP data sets obtained from four different stages of embryonic development, in conjunction with over 300 TF motifs and stage-specific DNA accessibility and RNA-SEQ data. Our ultimate goal is to use the insights revealed here, both general and data set-specific, to develop improved computational tools that can quantify functional TF-DNA interactions genome-wide. Such tools can potentially inform models of TF regulatory networks in the same way that ChIP data is beginning to be used today [1], [4]. We note that characterizing hundreds of TFs by the whole-genome ChIP-SEQ in the vast number of different cellular conditions is not currently feasible. Computational tools therefore offer an attractive alternative, especially if they can be shown to predict cell type-specific occupancy. TF motifs are already being characterized through high throughput technologies such as Bacterial 1-Hybrid [9], SELEX [11], [54], and Protein-Binding Microarrays [55]. Cell type-specific DNA accessibility profiles and TF expression levels only need to be characterized once for a given cell state, and can then be used to predict binding profiles for all TFs. Our work provides initial evidence for the feasibility of this vision. At the same time, we note that the CC values reported here should not be interpreted as correlation coefficients between genome-wide predictions and observed levels of TF binding. The manner in which we chose to evaluate various models, i.e., by examining agreement with ChIP scores on 1000 bound regions and 1000 randomly selected non-peaks, was dictated primarily by the goal of detecting significant influences on primary TF occupancy. We also note that the CC values varied substantially across data sets, from 0.765 for TRL to 0.062 for Dorsal (DL) (Table 1). This variation in model performance may reflect weaknesses of certain data sets or PWMs, or a variable reliance of ChIP scores on the primary TF's binding.

Despite a general appreciation of the potential role of various determinants of TF binding, there have been very few systematic studies of the extent of their influence across a large number of TFs. We review three such studies that set the stage for our own work and explain the main goals and contributions of our work in the backdrop of these important prior studies.

Kaplan et al. [6] studied ChIP-SEQ data on five TFs in early *Drosophila* development, and concluded that the TF motif and DNA accessibility are the most informative correlates of TF-DNA binding, as determined by the agreement between measured and predicted occupancy profiles. They also used TF sequence signatures to examine the role of competitive and cooperative interactions with other TFs with similar developmental roles and concluded that these interactions do not play a significant role overall. Their negative finding regarding secondary motifs may be limited to the small number of data sets examined, or be a limitation of the specific methodology adopted in the study (including the use of a more limited set of motifs that were available then). Here, we perform much more extensive tests of the role of the above-mentioned binding determinants of TF binding, by analyzing 45 TF-ChIP data sets spanning multiple stages of embryonic development in *D. melanogaster*. We primarily consider the influence of a large number of secondary TFs that are highly expressed in that developmental stage. In contrast to the earlier findings, we find many cases where the primary TF's binding levels are significantly influenced by the presence or absence of binding sites for other TFs.

In a related study, Pique-Regi et al. [7] considered the problem of classifying primary motif matches within ChIP peaks versus those outside of ChIP peaks, in the context of six ChIP-SEQ data sets from two human cell lines. They found accessibility and specific histone modifications to be the most useful features in this classification task, but did not consider the influence of secondary TFs. However, there are fundamental differences in the goals of our study from that of Pique-Regi et al. Their objective was to build a computational tool for annotating TF-bound sites genome-wide, and therefore their algorithm integrates several variables that correlate with binding, including evolutionary conservation, transcription start site proximity, DNA accessibility and histone marks. On the other hand, our focus is on the influence of variables that are expected to be mechanistic determinants of binding, and whose influence can be reasonably understood within an intuitive biophysical framework. We therefore focus specifically on testing whether and how binding sites of secondary TFs shape the primary TF's binding profile. In this pursuit, we rely upon motif, sequence and TF expression data, treating these as the “predictor variables” with which to model ChIP data. We do not include other variables such as evolutionary conservation (which is not a mechanistic determinant) or start site proximity (whose influence cannot be easily modeled biophysically) as predictors in this statistical exercise. DNA accessibility data is used in our analysis, not to improve occupancy prediction per se, but to answer a specific mechanistic question about how secondary TFs influence binding. Also, there is a fundamental technical difference between the data types modeled in the two studies: the variable we propose to model is not tied to TF-DNA interaction at an individual binding site as in [7], but to the aggregate effect of all binding events within a 500 bp window. For the simplicity, we ask whether a model can predict the actual ChIP score at a genomic position, rather than ask whether a model can predict whether a putative motif match falls within a significant ChIP peak or not.

A recent study by Yanez-Cuna et al. [5] searched for motif signatures of context specific binding of TFs. In particular, they analyzed ChIP data sets for the same TF from two different cellular conditions and asked if peaks exclusive to either condition could be discriminated on the basis of motif presence. They showed that such motif signatures do exist for the seven TFs examined and that general-purpose machine learning methods such as support vector machines can accurately classify context-specific binding sites using tens of motifs. In the same vein, they showed that bound and non-bound regions of a TF can be discriminated using a combination of tens of motifs, for most of the 21 TF-ChIP data sets examined. Additionally, they performed a closer examination of the binding determinants of one particular TF, twist (TWI), and demonstrated that binding sites for the secondary TFs VFL and TTK significantly affect the correct prediction of many context-specific TWI binding sites. While Yanez-Cuna et al. mostly focused on demonstrating that accessory motif signatures *can distinguish* TF-DNA binding regions in different developmental stages, our primary goal was to precisely *identify* the most influential secondary motifs for each of 45 different TF-ChIP data sets. To this end, we focused largely on quantifying the influence of secondary motifs and assessing their statistical significance rigorously. By performing our analysis over many data sets, we were able to gain more general insights about the widespread or TF-specific roles of particular secondary TFs. In particular, our statistical tests are geared towards explaining the mechanistic basis of such roles: short- versus long-range effects, synergistic versus antagonistic effects, chromatin mediated versus direct interactions, etc.

The review by Biggin [56] uses findings from recent studies to argue that accessibility is more important than the role of secondary TFs in determining primary TF binding levels. However, we do not attempt here to characterize the effect of accessibility as being stronger or weaker than the effect of interacting TFs. Integrating perspectives from Biggin and others [15]–[17], [57], [58], DNA accessibility *in vivo* can be considered the result of multiple factors playing out simultaneously, possibly including innate sequence preferences of nucleosome location, a conglomerate of chromatin remodeling activities and displacement of nucleosomes by competition with TF binding. Under this view, there are practical limitations in the approach of directly comparing the improvement in occupancy prediction due to accessibility information to that due to secondary motif information alone. Moreover, while it may be possible to make broad statements regarding the influence of accessibility or other chromatin-related information on TF binding, secondary TFs , due to the combinatorial nature of gene regulation, will be factor-specific in their effects and thus will only be detectable on a few data sets. Accordingly, our goal is to characterize as many of these determinants of TF occupancy, from each ChIP data set, rather than assign any one number to the overall influence of, say, interactions between the primary and secondary TFs, which will be factor dependent by definition.

A related study that examined the effects of secondary TFs on ChIP data is that of Gordan et al. [59] who reported on TF-ChIP data sets in yeast where a secondary motif alone was a better correlate of peak location than the primary motif. In some cases, this may be due to a problem with the primary motif (H.N.P. and M.H.B. unpublished results). In other cases, such a situation may reflect indirect binding of the primary TF to the peak, via physical interaction with the bound secondary TF. It suggests an alternative model of ChIP data, where binding is predicted to be a sum or linear combination of the occupancy values of the primary TF (direct binding) and a secondary TF (indirect binding). We have not explored this model here, and believe that it is an important goal for future studies.

Our approach to including accessibility data in the analysis was to use partial correlations to examine secondary TF influences before and after factoring out the effect of accessibility on ChIP scores. Alternative approaches may directly include accessibility data in the occupancy models, as was done by Kaplan et al [6], who changed prior probabilities of binding in their probabilistic model based on accessibility, and Pique-Regi et al. [7], who included DHS and histone modification data as features in their classifier. Future modifications of our approach will attempt to include accessibility within the biophysical framework of STAP, and may potentially reveal the role of accessibility even more accurately.

An intriguing observation from our analyses was the influence of competitive binding by the secondary TF EXD despite there being no correlation between EXD sites and the ChIP scores of the primary TF. It is puzzling because it suggests that the frequency of EXD sites does not differ between peaks and non-peaks, yet these sites somehow make a significant difference to binding predictions. However, it is possible that the frequency of EXD sites overlapping with primary TF sites is different between peaks and non-peaks, and the advanced model uses the competition for overlapping sites to predict lower occupancy in certain sequences than that predicted by the baseline model, leading to improved agreement with ChIP scores (Supplementary Figure S11).

Our work opens up several important directions of future research into TF-DNA interaction on a genomic scale. While the models we explored used at most one secondary motif in one interaction mode, a more realistic model will require integration of more than one underlying mechanisms influencing primary TF occupancy. Accessibility information will play a crucial role in the predictive ability of such models. In the longer term, an important goal will be to develop integrative models where sequence, TF gene expression and developmental history is sufficient to predict, at least to a good approximation, both accessibility patterns and TF-DNA binding profiles. With the future availability of large collections of TF motifs, such computational surrogates for cell type-specific ChIP data will enable global studies of gene regulatory networks and provide specific regulatory assignments that can be experimentally confirmed.

## Materials and Methods

### Data

We used 55 TF-ChIP data sets on 37 TFs active in early stages of *Drosophila* embryonic development. These include five ChIP-seq data sets and 20 ChIP-chip data sets from BDTNP [60], seven ChIP-chip data sets from the Furlong lab [24], and 21 normalized ChIP-chip and ChIP-seq data sets from the ModEncode project [1], [61]. ChIP data of VFL and TRL were obtained respectively from [29] and [62]. Stage-specific genome-wide DNaseI hypersensitivity (chromatin accessibility) data, which is mapped to genome release 4 coordinates, was downloaded from the first replicate in the BDTNP web site and converted to release 5 coordinates using the liftOver tool and chain files from the UCSC web site (http://hgdownload.cse.ucsc.edu/downloads.html). We used 614 *Drosophila* transcription factor motifs, corresponding to 322 distinct TFs, from the FlyFactorSurvey database [20]. The motifs were ranked based on expression of the associated TF gene, using RNA-SEQ data [21] for the appropriate developmental stage. In cases where a TF-ChIP data set corresponded to a range of stages, expression values were stage-normalized and averaged before ranking. Motifs corresponding to heterodimeric complexes (such as HLH TFs in complex with DNA) were not considered. Motifs in the top 10% of the expression-based ranked list for the appropriate developmental stage were tested as candidate secondary motifs. The one exception to this are results in Table 3 where the top 25% of the ranked list was considered.

We smoothed each TF-ChIP data set and each DNase I data set by assigning scores to each 500 bps window over the genome, with a 50 bps shift. First, raw “read scores” in a data set were mapped to the nearest genomic position that is a multiple of 50. The score of a 500 bp window was then computed by averaging over all read scores mapped to positions in that window; we refer to this as the “ChIP score” of the window. After this transformation, we selected 1000 non-overlapping, highest scoring windows as “peaks” and randomly extracted 1000 non-exonic, non-overlapping windows without replacement from the remaining genome as “non-peaks”. This set of 2000 windows and their ChIP scores constitutes a TF- and stage-specific data set in our analyses. A “primary” motif was designated for the data set, based on the availability of motifs for the ChIP'ed TF. In cases where there were multiple motifs available for the ChIP'ed TF, the motif with the highest correlation between STAP scores and ChIP scores over all 2000 windows (see below) was selected. “Secondary” motifs tested for potential effects on the primary TF's binding were selected based on expression data, as mentioned above.

### Evaluations of STAP-based TF-DNA occupancy prediction by 4-fold cross-validation

We used the STAP program [22] to predict the ChIP score of a window, using the primary motif and optionally a secondary motif for that TF. STAP has one or more free parameters that require training data – a set of sequences and their ChIP scores. Hence, we used cross-validation to train and test various models of TF-DNA occupancy that are encoded by STAP. We randomly divided the 1000 peaks into 4 equal partitions and also the 1000 non-peaks into 4 equal partitions. In each fold of cross-validation, three partitions from the peaks and non-peaks were used as the training set and one partition (i.e., 250 peaks and 250 non-peaks) was the test set. Predicted ChIP-scores on each of the test sets of windows were collected together, and the resulting set of 2000 real and predicted ChIP score pairs were subjected to evaluations. Evaluations on a data set were considered a failure if the STAP parameter values learned in the four folds were widely different; this happened for one data set.

### The STAP model

This was described in [22]. STAP considers each molecular configuration σ that specifies which sites in the given sequence are bound by their respective TFs. Following standard statistical physics, the “Boltzmann weight” of σ, denoted by W(σ), represents the relative probability of the system being in configuration σ, and is calculated based on TF concentration and the estimated binding affinity of every bound site in σ. The Boltzmann weight is a product of terms contributed by each TF-bound site in the configuration. This corresponds to the assumption that each bound TF interacts independently with the DNA, with energy contributions that add up [63]. See Figure 1A for an example where the sequence has two sites (‘A’ and ‘B’) for TF ‘A’, or Figure 1B where there is one site for each of two TFs ‘A’ and ‘C’. A site's contribution, *q(S)*, depends on the TF concentration and the strength of site *S*, and is given by:
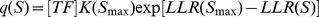
where [TF] is the concentration of the TF (in arbitrary units), *LLR*(⋅) is the log likelihood ratio score of a site, computed based on the known position weight matrix (PWM) of the TF [25], *S_max_* is the strongest binding site of the TF, and *K(S_max_)* is the equilibrium constant of the TF binding to this site. The product *K(S_max_)[TF]* is a TF-specific free parameter denoted by γ_TF_. Let *N_k_*(σ) denote the number of bound sites of TF *k* in configuration σ. The STAP model predicts the occupancy of TF *k* as:
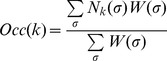
Note that while *N_k_*(σ) counts the number of bound sites for TF *k* only, the Boltzmann weight *W*(σ) depends on bound sites for all TFs.

### Assessment of statistical significance by P-value and Z-score

The accuracy of STAP predictions was assessed by computing the Pearson correlation coefficient (CC) between real and predicted ChIP scores of 2000 windows in a data set. To assess the impact of a secondary motif *M_2_* in modeling a data set whose primary motif is *M_1_*, we tested STAP in a single motif mode (“STAP(M_1_)”) and in two-motif mode (“STAP(M_1_,M_2_)”) and compared the difference in their accuracies: *ΔCC = CC(STAP(M_1_,M_2_)) – CC(STAP(M_1_))*.

A secondary motif *M_2_* was deemed as a significant influence on the data set if the following conditions were met:

1. *ΔCC ≥*0.04. This is used as a basic criterion before performing further assessments. It is a heuristic for reducing the number of tests we had to perform, thus speeding up the analysis.
2. *ΔCC′ = CC(STAP(M_1_,M_2_)) – CC(STAP(M_2_))* ≥0.04. This is to ensure that the model when using both the primary and the secondary motif fits the data better than a model using the secondary motif alone (without the primary motif).
3. *P-value(ΔCC)* ≤0.05. To compute this, we permuted the secondary motif by randomly shuffling rows and columns of the PWM, which keeps the motif information content intact. ΔCC was computed for each permuted version, and by repeating this 100 times, we estimated a p-value for the original ΔCC.

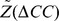
 *≥3*. Here, 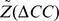 is a “Z-score” that compares the given ΔCC to corresponding values obtained from every candidate secondary motif that was tested, and is analogous to a multiple hypothesis testing correction. It is a measure borrowed from outlier detection theory [64], and reflects if the observed ΔCC is an outlier compared to a given set of ΔCC values. It is defined as 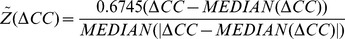

We also evaluated the best secondary motif effect for each data set by computing an “Area Under ROC” (AUC) value for the interaction model (Supplementary Table S8).

### Tests of inter-site spacing bias

For each significant case of cooperative or antagonistic influence by a secondary motif, we searched for biases in the inter-site spacing between the primary and secondary TFs. Let us assume a pair of motifs (M1, M2) represents the binding specificities of the primary and secondary TFs. To test for a specific spacing bias, say ‘*d*’ base pairs, between (M1, M2) in a given set of segments, we grouped all pairs of adjacent heterotypic binding sites (located by FIMO program with threshold of e^−7^ [65]) into those having or not having inter-site distance of *d*. We counted the number of site pairs in each group and compared these counts to the corresponding counts in a “background” data set using one-tailed Fisher's exact test. The “background” data set was constructed by shuffling the locations of predicted sites in each segment, thus preserving the number of binding sites in each segment, and pooling together 10 such randomized data sets (Kazemian et al., manuscript in review). Tests of spacing bias were conducted on a set of top 250 scoring ChIP peaks and separately on a set of bottom 250 non-peaks.

### Semi-partial correlation

Semi-partial correlation is a statistical technique generally employed to assess the association of one random variable X with the other random variable Y after eliminating the effect of a third random variable Z on Y [66]. In our tests, X represents predicted TF-DNA binding, Y the experimental TF-DNA occupancy from ChIP, and Z the accessibility. The semi-partial correlation score 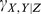 between X and Y, after “partialing out” Z from Y, is computed as 
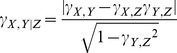
, where 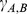 is the correlation coefficient between *A* and *B*.

### *In vitro* analysis of TF-TF interactions

Protein interactions were measured in a modification of the previously described LUMIER or LuMPIS methods [38], [39] except that each protein was expressed *in vitro* rather than in cell culture. Open reading frame (ORF) clones for transcription factors were part of the Berkeley *Drosophila* Genome Project the collection of universal donor clones [67]. ORFs were transferred into two vectors, pHPT7-FlRluc-BD and pHPT7-MBP-BD (HNP and MHB, unpublished), using Cre Recombinase (New England Biolabs, M0298L). For one TF, Mad, the ORF was PCR amplified ligated into AscI and PmeI restriction sites in each vector. These vectors contain a T7 promoter for *in vitro* transcription, a loxP site for cloning and either maltose binding protein (MBP) or *Renilla* luciferase (luc) coding regions. Clone names and primer sequences are provided in the supplementary information (Table S9).

Proteins were made by coupled *in vitro* transcription/translation using the PURExpress *In Vitro* Protein Synthesis Kit (NEB, E6800S). All samples were analyzed by Western Blot to confirm that some full-length product was obtained. Luciferase input was measured using the *Renilla* Luciferase Assay System (Promega, E2820). The proteins were diluted with IP Buffer (150 mM NaCl, 50 mM Tris pH 7.4) such that roughly 10^6^ luciferase counts were added to each sample and an equivalent amount of MBP protein were mixed. Proteins were incubated with gentle rocking for 4°C for 2 hours. Amylose Resin (NEB, E8021S) blocked with 5 percent BSA was added to proteins and incubated with rocking at 4°C for 2 hours. The samples were washed twice with IP buffer and transferred to 96-well plates (Corning, 07-200-589) for luciferase measurements. The luminescence intensity ratio was measured using as follows:
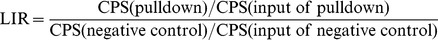



Each experiment was performed in duplicate, the experiments were averaged, and the standard deviation was calculated. Source cDNAs, amplification primers and luciferase data are compiled in Supplementary Table S9.

### *In vitro* analysis of TF binding

*In vitro* synthesis of tagged TFs and luciferase assays were performed as described above. Target sequences were identified from the top ChIP peak regions that contained strong matches to the primary and secondary motifs with a spacing and orientation that was most frequently observed. Other criteria used in selecting target sequences included whether the ChIP peak lies within a known enhancer, and whether its predicted occupancy under STAP's cooperativity model is higher (in rank) than that under the baseline model without cooperativity. Double stranded DNA oligonucleotides were synthesized that contained wild type or altered sequences. One oligonucleotide containing the wild type sequence is biotinylated on the first base. The genomic coordinates for the wild type sequences and all mutant sequences are shown in Supplementary Table S16. Protein-DNA interactions were measured in a modification of a previously described microwell-based assay [46]. Tagged TFs were expressed *in vitro* rather than in cell culture and diluted with low-stringency binding buffer (140 mM KCl, 5 mM NaCl, 1 mM K_2_HPO_4_, 2 mM MgSO_4_, 20 mM HEPES (pH 7.05), 100 µM EDTA, 1 µM ZnSO4) +1% BSA. Oligonucleotides were annealed and diluted using annealing buffer (50 mM Tris-HCl, 0.2 mM MgSO_4_, pH 7.0). Annealed oligo mixes were prepared with 5 ul of 1.2 uM biotinylated oligos, 5 ul of 24 µM competitor oligo, 2 ul of 500 ng/ul Poly(dI-dC)*Poly(dI-dC), and 8 ul of annealing buffer (final volume 20 ul) and incubated for 1 hour. 10^6^ luciferase counts of the luc-tagged primary TF and (if appropriate) an equivalent amount of MBP-tagged secondary TF were mixed (30 ul volume). The diluted proteins were added to the DNAs and incubated with gentle rocking at 4°C for 2 hours. Streptavidin coated 96 well plates (ThermoScientific # 15502) were blocked with 5% BSA and low stringency binding buffer. The protein/oligo mixture was added to the plates and incubated for 2 hours at 4°C. The samples were washed twice with low stringency binding buffer. Recovered luciferase activity was measured directly in the plates. All values were normalized by dividing by the luciferase counts recovered in the sample containing an excess of wild type competitor DNA.

## Supporting Information

Figure S1 Detailed examination of ChIP scores and STAP scores for all 2000 segments and Receiver Operating Characteristic (ROC) curve for “BIN_Fchip_s14”, which is the data set with the second best CC overall. In the scatter plot (left), blue and red points represent the 1000 top ChIP peaks and 1000 randomly selected non-coding segments respectively. The ROC (right panel) represents a classifier that uses a threshold on the STAP score to discriminate TF-bound segments from non-bound segments, defined by the top 50% and bottom 50% ChIP scores. The Area Under the ROC curve (AUC) is 0.895.
(TIFF)

Figure S2 Receiver Operating Characteristic (ROC) curve for the data set “HKB_Bchip_s5”. The AUC is 0.679. The CC on this data set is 0.305, which is approximately the average CC over all 45 data sets shown in Table 1.
(TIFF)

Figure S3 Spacing bias analysis of two data sets, with VFL as the secondary motif, where cooperative influence was detected even after partialing out accessibility.
(TIFF)

Figure S4 ROC plots of the baseline model and best TF-TF interaction models for two data sets, BCD_Bseq_s5 and HKB_Mseq_s14. BCD and VFL are modeled to exhibit a cooperative TF-TF interaction, and HKB and JIGR1 are modeled to exhibit an antagonistic interaction.
(TIFF)

Figure S5 Contributions of peaks and non-peaks to the CC values reported for the single motif STAP model in Table 1. (Also see Table S10.) A. Comparison of model performance on peaks to the overall performance on the 45 data sets. Each point represents a data set. CC(M1) is the correlation coefficient of the baseline model driven by the primary motif M1. The horizontal and vertical dotted blue lines denote CC = 0.15, while the diagonal dotted blue line represents x = y in this chart. B. Comparison of model performance on peaks to the performance on non-peaks on the 45 data sets. C. Model performance may vary with the strength of the in vivo TF-DNA occupancy. The x-axis shows the 1000 peaks in the data set “BCD_Bseq_s5” divided into eight bins of 125 segments each based on ChIP scores. The top 125 most highly occupied genomic windows (rightmost bin) show the highest CC of 0.486 between in vivo occupancy and STAP prediction. The overall CC is 0.560, CC on all 1000 peaks taken together is 0.466, and CC on non-peaks is 0.050. D. Scatterplot of ChIP scores and STAP scores for all 2000 genomic windows in the data set “MAD_Bchip_s5”, where overall CC is 0.635, CC on peaks is −0.206, and CC on the 125 most highly occupied windows is −0.279. Green, blue and red points represent the 125 top ChIP peaks, 875 next highest ChIP peaks and 1000 randomly selected non-overlapping non-coding genomic windows respectively.
(TIFF)

Figure S6 Effect of long-range cooperative interactions between pairs of TFs on the accuracy of modeling ChIP scores within peaks and non-peaks. We calculated the correlation coefficient (CC) between the in vivo occupancy and our STAP prediction on peaks and non-peaks for each of 18 TF-ChIP data sets from Table 2. (Also see Table S11.) A. Comparison of model performance on peaks to the overall performance on the 18 data sets. Each point represents a data set. CC(M1, M2) is the correlation coefficient of the cooperativity model driven by the primary motif M1 and the secondary motif M2. B. Comparison of model performance on peaks to the performance on non-peaks on these data sets. C. Comparison of the performance improvement on peaks to that on the entire data set. ΔCC (M1, M2) = CC(M1, M2) – CC(M1). D. Comparison of the performance improvement on peaks to that on non-peaks.
(TIFF)

Figure S7 Effect of short-range cooperative interactions between pairs of TFs on the accuracy of modeling ChIP scores within peaks and non-peaks. We calculated the correlation coefficient (CC) between in vivo occupancy and our STAP prediction on peaks and non-peaks for seven TF-ChIP data sets where significant short-range TF-TF cooperativity has been identified (refer to Table 3 and Table S12). Shown is a comparison of performance improvement on peaks to that on the entire data set. Each point represents a data set. CC(M1, M2) is the correlation coefficient of the cooperativity model with primary motif M1 and secondary motif M2. ΔCC(M1, M2) = CC(M1, M2) – CC(M1).
(TIFF)

Figure S8 Effect of antagonistic interactions between pairs of TFs on the accuracy of modeling ChIP data, for peaks and non-peaks separately. We calculated the correlation coefficient (CC) between in vivo occupancy and our STAP prediction on peaks and non-peaks for 35 cases of antagonism where significant TF-TF antagonism was identified in Table 5. (Also see Table S13.) A. Comparison of the performance improvement on peaks with that on the entire data set. Each point represents a data set. CC(M1, M2) is the correlation coefficient of the antagonism model with primary motif M1 and secondary motif M2. ΔCC (M1, M2) = CC(M1, M2) – CC(M1). B. Similar to (A), except that the x axis represents ΔCC(M1,M2) on non-peaks rather than peaks. C. Comparison of the performance improvement on peaks to that on non-peaks. Red symbols in both panels represent cases where the improvement on either peaks or non-peaks is larger than the improvement on the entire data set. Blue symbols represent all other data sets.
(TIFF)

Figure S9 Performance of the baseline (single motif) model using two different definitions of the “negative set.” By default each TF-ChIP data set comprised the top 1000 peaks and 1000 random non-coding sequence windows (non-peaks). All results in the main text are based on this definition of non-peaks. Here, we replaced the 1000 randomly chosen non-peaks with 1000 randomly chosen non-peaks that happen to be ChIP peaks of a different TF. The plot shows CC values of the STAP model on each data set, using these two definitions of non-peaks (x-axis corresponds to the default definition). Red symbols represent data sets where the CC with the default definition of non-peaks is better than the CC with the new definition of non-peaks (by 0.04 or more). Green symbols represent data sets where the CC with the new definition is better than the CC with the default definition of non-peaks (by 0.04 or more). (Also refer to Table S15.)
(TIFF)

Figure S10 Detailed examination of ChIP scores and STAP scores on data set UBX_Mchip_s5_14, which shows a pronounced increase in CC when random non-peaks in the data set are replaced by non-peaks randomly chosen from peaks of other data sets. A. In-vivo TF-DNA occupancy versus STAP baseline model prediction on 2000 genomic windows in the default data set that includes 1000 non-overlapping non-exonic non-peaks extracted randomly from the whole genome. B. In-vivo TF-DNA occupancy versus STAP baseline model prediction on 2000 genomic windows that includes 1000 non-peaks of the TF chosen randomly from peaks of other data sets (corresponding to other TFs).
(TIFF)

Figure S11 Influence of the competing DNA binding of HB and EXD to the TF-DNA occupancy prediction for the data set HB_Bchip_S9. We trained the STAP baseline model (HB only) without 4-fold cross validation and obtained HB's binding weight parameter value γ_HB_. Then we fit the STAP competition model by fixing the binding weight parameter value of HB as γ_HB_ and setting the binding weight parameter of EXD as the only free parameter. The scatter plot shows the predicted TF-DNA occupancy score of each sequence (peak and non-peak) as per the baseline model with primary TF only and the advanced model that includes competitive binding by EXD. From these plots, we note that many peaks (red) as well as non-peaks (blue) fall below the diagonal, which represents a lower STAP score from the advanced model (with competition) than from the baseline model (without competition). This suggests that the advanced model, to its advantage, in both peaks and non-peaks, is exploiting overlapping sites.
(TIFF)

Figure S12 Experimental validation of predicted direct TF-TF interactions. This chart is an extended version of Figure 4C (right panel) but with additional negative controls with either CLK or empty vector (MT). GT-GT, MAD-MED and CLK-CYC positive controls are shown. The same chart also appears in Supplementary Table S9 (Excel file, worksheet named “heterodimers + more controls”).
(TIFF)

Table S1 Ten data sets excluded from detailed analysis. These include seven data sets for which no model was able to achieve CC above the chosen threshold (≥0.15) (top seven rows). FTZ_Bchip_s5 and RUN_Mchip_s5_14 are excluded due to the negative association between ChIP profile and the estimated TF-DNA occupancy in the single-motif baseline model. GATAE_Mchip_s5_11 is also disregarded since 1) the parameter values learned from different folds of the cross-validation experiment were widely different, and 2) the learned parameter values were sensitive to the site threshold used in STAP. We categorized these “failed” data sets into two classes: C1 = only one data set was examined for this TF, so both the model and data set quality are suspect; C2 = multiple data sets were examined for this TF (from different sources and/or developmental stages) and at least one data set shows a CC ≥0.15, suggesting that this failed data set is suspect rather than the model.
(TIFF)

Table S2 Effect of cooperative interactions between pairs of TFs on the accuracy of modeling ChIP data. Shown here are all cases where P-value is < = 0.05 and Z-score is > = 3 at distance threshold of 150 bps. Column semantics are as in Table 2 of the main text.
(TIFF)

Table S3 Cases of significant influence of VFL, in cooperativity mode with distance threshold = 150 bp. Column semantics are as in Table 2 of the main text. A ‘-’ indicates that the effect was insignificant.
(TIFF)

Table S4 Cases of significant influence of TRL, in cooperativity model with distance threshold equal to 150 bp. Column semantics are as in Table 2 of the main text. A ‘-’ indicates that the effect was insignificant.
(TIFF)

Table S5 Spacing bias analysis for antagonistic influences where the bias is significant in the non-peaks and not in peaks. Shown are cases where the STAP model, using the primary motif and the secondary motif listed in column “Motif 2”, with antagonistic interaction at distance less than “Distance threshold”, led to significant improvement in CC on a data set. In each case, we tested for a bias for a spacing range [1–2, 2–3, 3–4, …, 29–30], in peaks and non-peaks separately, and report the lowest p-value observed across all spacing ranges.
(TIFF)

Table S6 Correlation coefficient between ChIP scores and each of two different computational scores: STAP predictions using the primary motif (CC(M1)) and STAP predictions using the primary motif but set to zero in inaccessible regions (CC(AccFilter(M1))).
(TIFF)

Table S7 Effect of VFL before (ΔCC) and after (ΔSPCC) partialing out accessibility. A substantial effect (≥0.04) remains even after partialing out accessibility for the data sets CAD_Bseq_s5 and HB_Bseq_s5, while a borderline significant effect remains for the data set KR_Bseq_s5.
(TIFF)

Table S8 The CC and AUC scores of the baseline model and best TF-TF interaction model of 45 amenable data set. CCImprOverM1 is the difference of CC(M1+M2) and CC(M1), where M2 is a best secondary motif candidate under the most beneficial TF-TF interaction model. AUCImprOverM1 is the corresponding AUC difference under these two models. Bold font in the two columns refers to cases where the improvement arising from the best model is > = 0.04.
(TIFF)

Table S9 Data tables for the in vitro pull down experiments characterizing the interaction between MBP and luciferase tagged transcription factors. For detailed explanation see worksheet “notes” in this file.
(XLSX)

Table S10 Evaluation of single motif STAP model on peaks and non-peaks of 45 TF-ChIP data sets (refer to Table 1). The “Overall” column is identical to the column labeled “CC(M1)” in Table 1.
(TIFF)

Table S11 Effect of long-range cooperative interactions between pairs of TFs on the accuracy of modeling ChIP scores within peaks and non-peaks (refer to Table 2). Rows where the data set name is in underlined font represent cases where the ΔCC on peaks or non-peaks is ≥0.04, and the corresponding CC(M1+M2) is ≥0.15.
(TIFF)

Table S12 Effect of short-range cooperative interactions between pairs of TFs on the accuracy of modeling ChIP scores within peaks and non-peaks (refer to Table 3). These cases correspond to the rows of Table 3 that are significant at distance threshold 30 bp and marked with asterisks. In none of these seven cases does ΔCC within peaks or within non-peaks rise above the nominal threshold of 0.04, suggesting that the observed improvements due to short-range cooperativity modeling (Table 3) arise mainly from a better discrimination of peaks from non-peaks.
(TIFF)

Table S13 Effect of antagonistic interactions between pairs of TFs on the accuracy of modeling ChIP **data**, for peaks and non-peaks separately (refer to Table 5).
(TIFF)

Table S14 Effects of cooperative DNA binding by the primary TF and a secondary TF (“M2”) chosen from the top 25–50% most highly expressed TF genes, that were significant at distance threshold either 30 bp or 150 bp, with P-value≤0.05 and Z-score ≥3. Cases where the improvement at distance threshold 30 bp was better than that at 150 bp are highlighted in bold. Dashes indicate that the secondary motif was not observed to show significant improvement at the specific distance threshold. This table presents results from testing all secondary motifs whose TFs were in the top 25–50% most highly expressed TF genes (out of the ∼300 TF genes with motifs) at the appropriate developmental stage.
(XLSX)

Table S15 Performance of the baseline (single motif) model using two different definitions of the “negative set.” CC(M1) is the Pearson correlation coefficient between STAP predictions and ChIP scores for each data set, which comprises the top 1000 ChIP peaks and 1000 randomly chosen non-peaks. CC(M1)^α^ is the correlation coefficient in a data set where the non-peaks are randomly chosen from the top 1000 ChIP peaks of other TFs. There are 20 data sets with CC(M1)^α^ > CC(M1), and for 11 of these (shown in green) the difference is 0.04 or more. We took UBX_Mchip_s5_14 as an example of such cases and examined the in vivo occupancy and STAP baseline model predictions for this data set in detail (see Figure S10). For the remaining 25 data sets we found CC(M1)^α^ < CC(M1), and for 11 of these, shown in red, the difference is 0.04 or more.
(XLSX)

Table S16 Data and oligo sequence information for cooperative DNA binding assays. Constructs are the same used in the in vitro pull down experiments. For detailed explanation see worksheet “notes” in this file.
(XLSX)

Table S17 All cases where VFL has a significant cooperativity effect at distance threshold = 30 bp or 150 bp, showing the effect at both distance thresholds. Column semantics are as in Table 3 of the main text. A ‘-’ indicates that the effect was insignificant.
(TIFF)

Table S18 All cases where TRL has a significant effect at distance threshold = either 30 bp or 150 bp, showing the effect at both distance thresholds. Column semantics are as in Table 3 of the main text. A ‘-’ indicates that the effect was insignificant.
(TIFF)

Table S19 Spacing bias analysis for antagonistic influences where the bias is significant in the peaks and not in non-peaks. Columns have semantics as in Table S5.
(TIFF)
