# Supplementary figures and images for: Computational Identification of Diverse Mechanisms Underlying Transcription Factor-DNA Occupancy
Source: PLoS Genet. 2013 Aug 1;9(8):e1003571. doi: 10.1371/journal.pgen.1003571 (PMC3731213; doi:10.1371/journal.pgen.1003571)

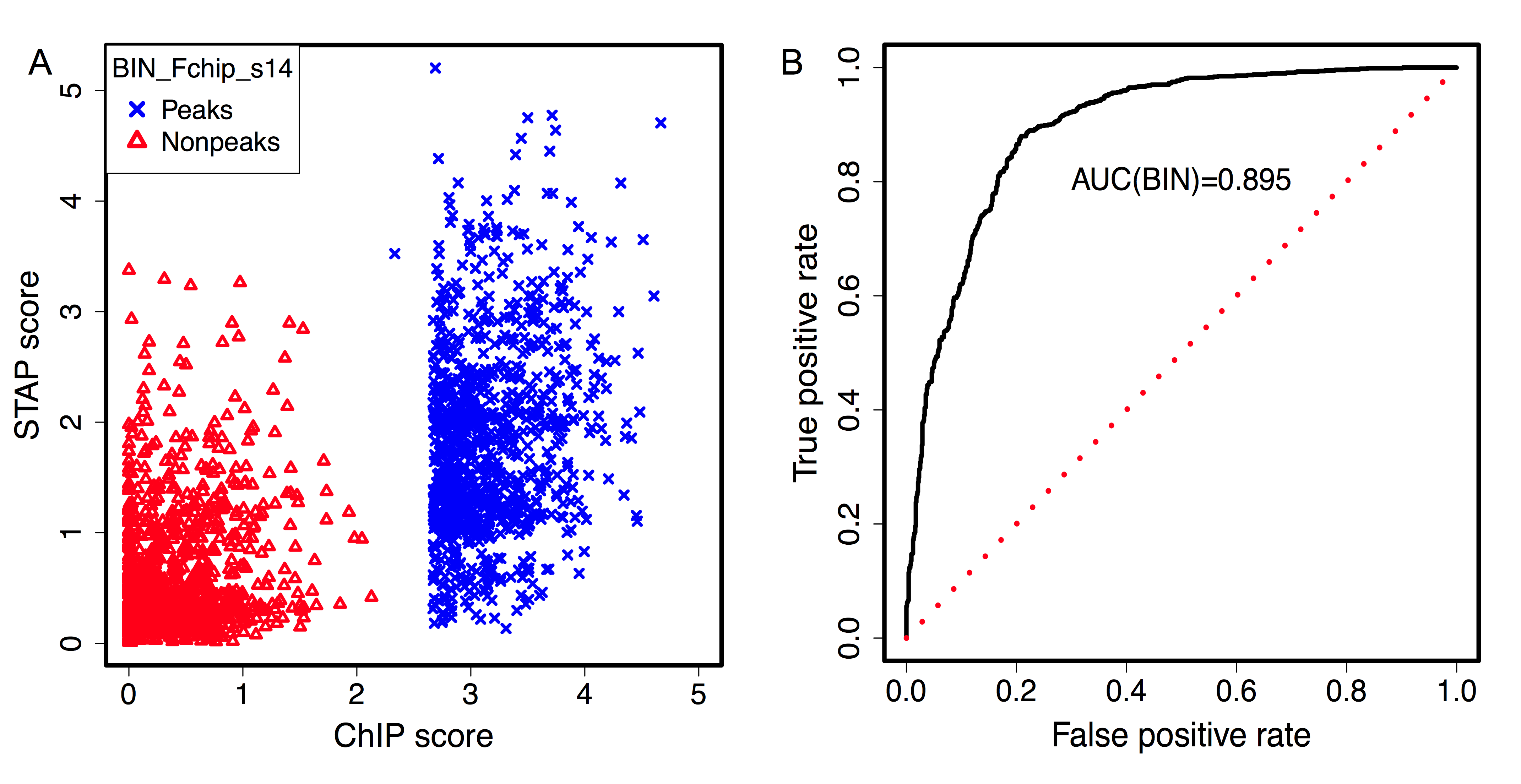

Supplement: Figure S1 — Detailed examination of ChIP scores and STAP scores for all 2000 segments and Receiver Operating Characteristic (ROC) curve for “BIN_Fchip_s14”, which is the data set with the second best CC overall. In the scatter plot (left), blue and red points represent the 1000 top ChIP peaks and 1000 randomly selected non-coding segments respectively. The ROC (right panel) represents a classifier that uses a threshold on the STAP score to discriminate TF-bound segments from non-bound segments, defined by the top 50% and bottom 50% ChIP scores. The Area Under the ROC curve (AUC) is 0.895. (TIFF) [file pgen.1003571.s001.tiff]

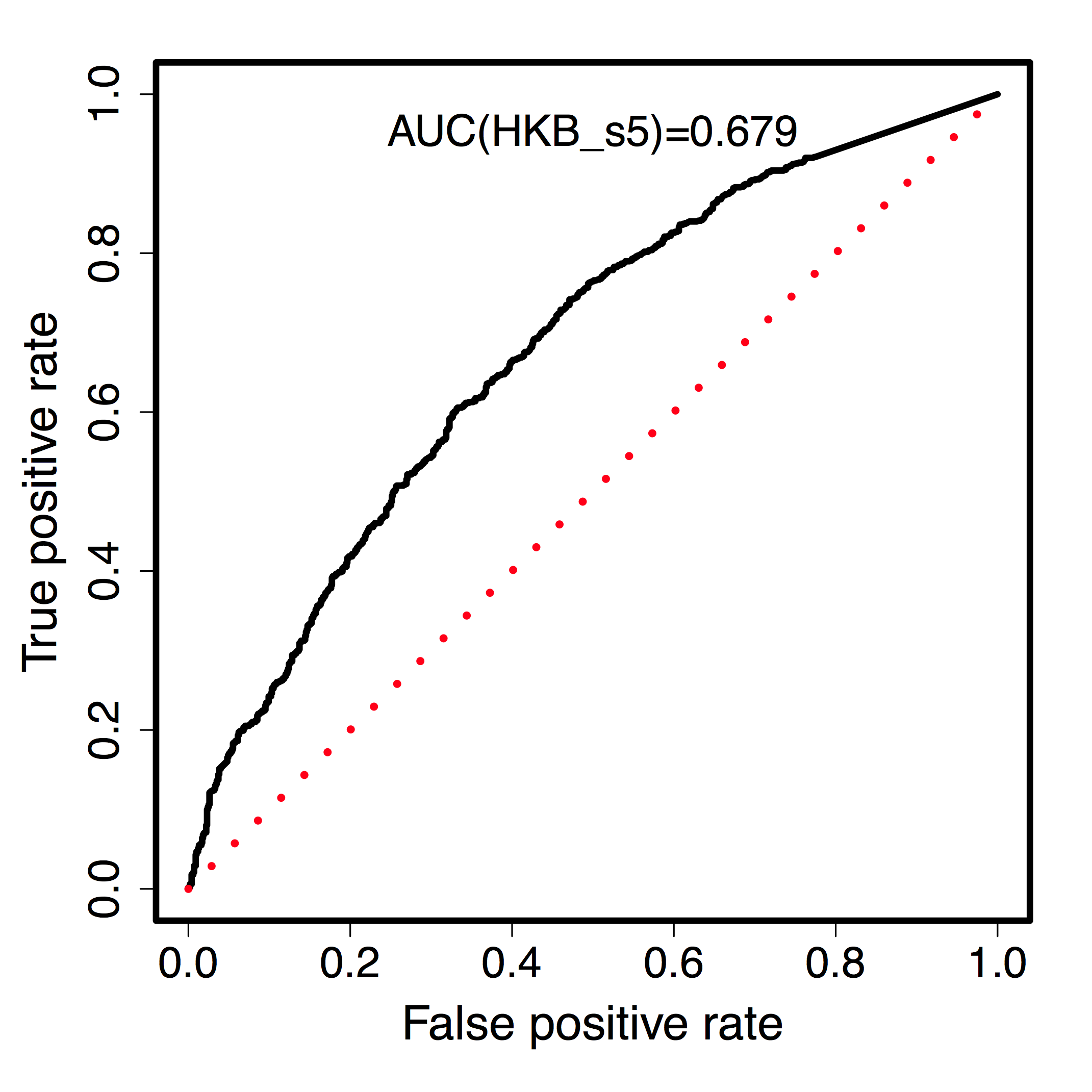

Supplement: Figure S2 — Receiver Operating Characteristic (ROC) curve for the data set “HKB_Bchip_s5”. The AUC is 0.679. The CC on this data set is 0.305, which is approximately the average CC over all 45 data sets shown in Table 1. (TIFF) [file pgen.1003571.s002.tiff]

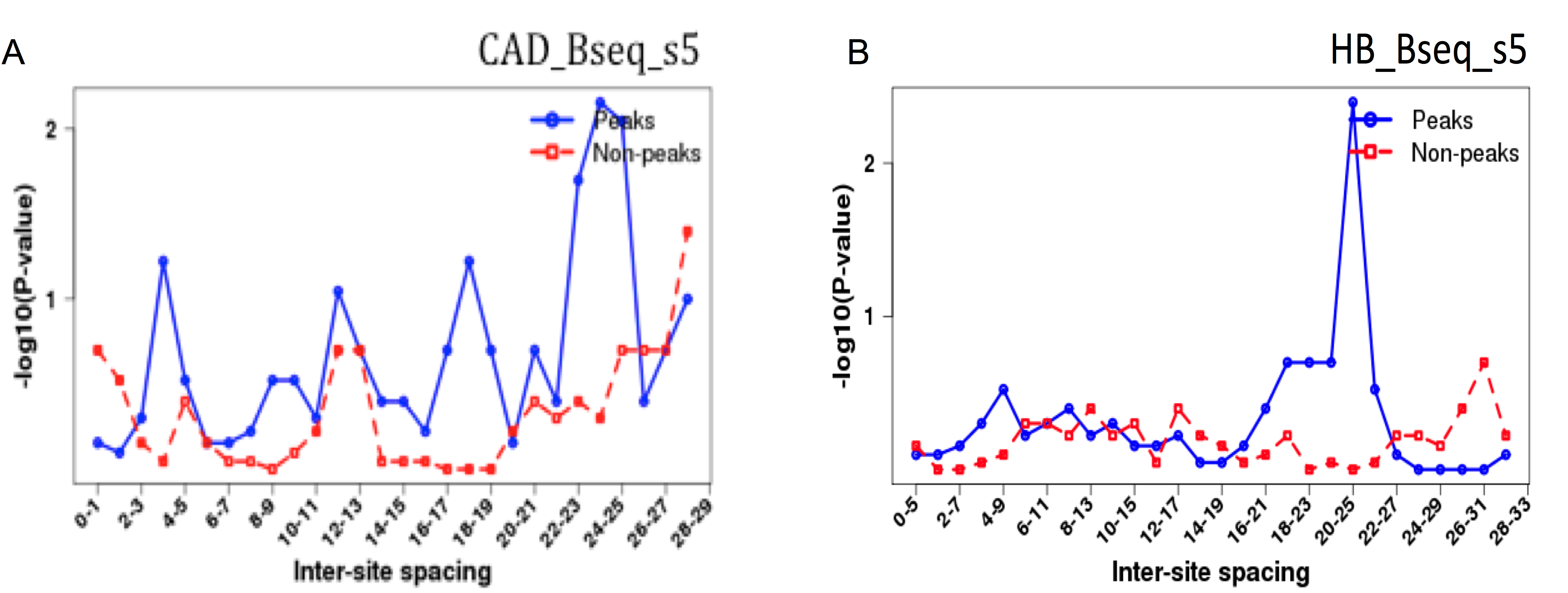

Supplement: Figure S3 — Spacing bias analysis of two data sets, with VFL as the secondary motif, where cooperative influence was detected even after partialing out accessibility. (TIFF) [file pgen.1003571.s003.tiff]

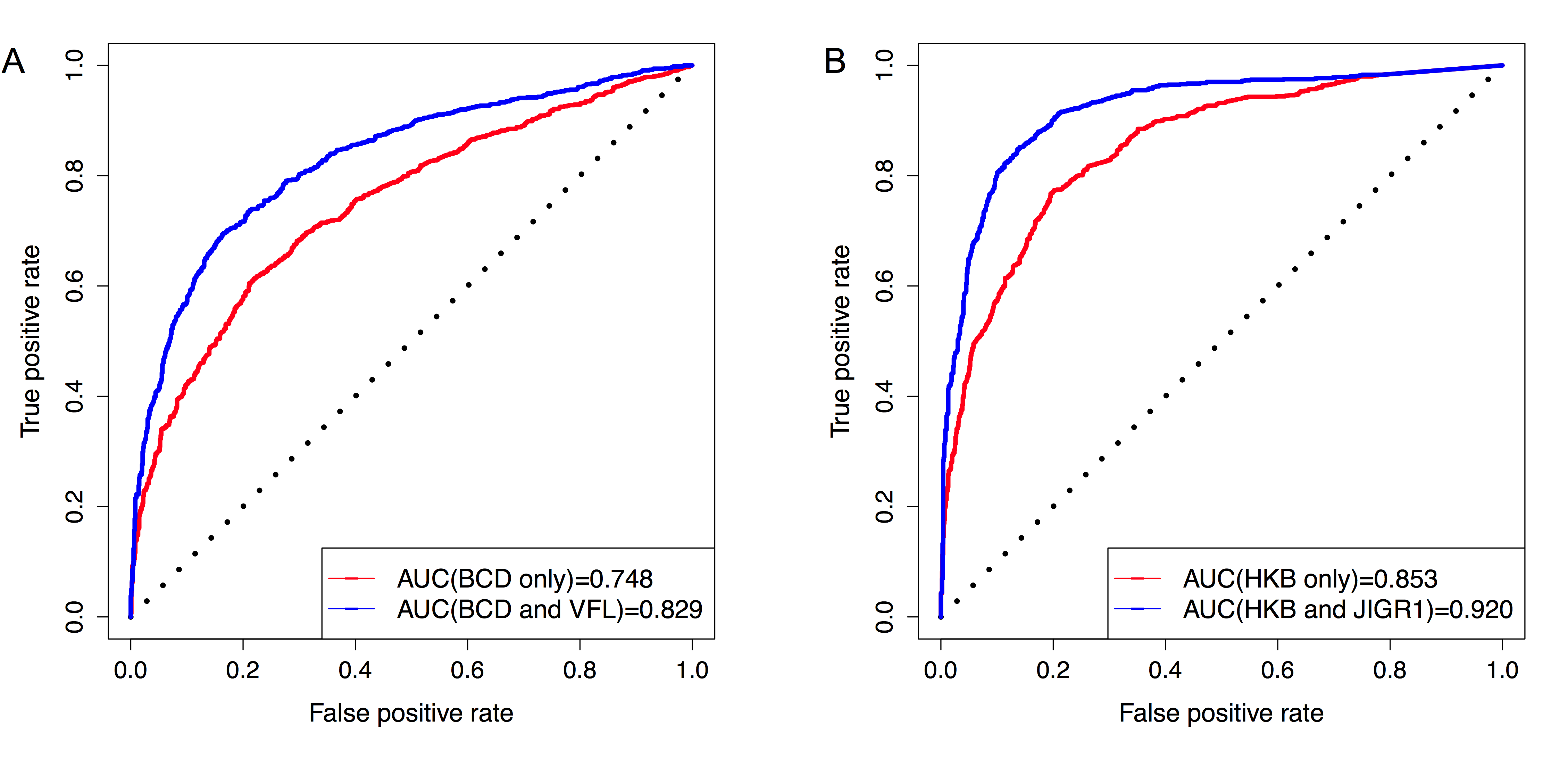

Supplement: Figure S4 — ROC plots of the baseline model and best TF-TF interaction models for two data sets, BCD_Bseq_s5 and HKB_Mseq_s14. BCD and VFL are modeled to exhibit a cooperative TF-TF interaction, and HKB and JIGR1 are modeled to exhibit an antagonistic interaction. (TIFF) [file pgen.1003571.s004.tiff]

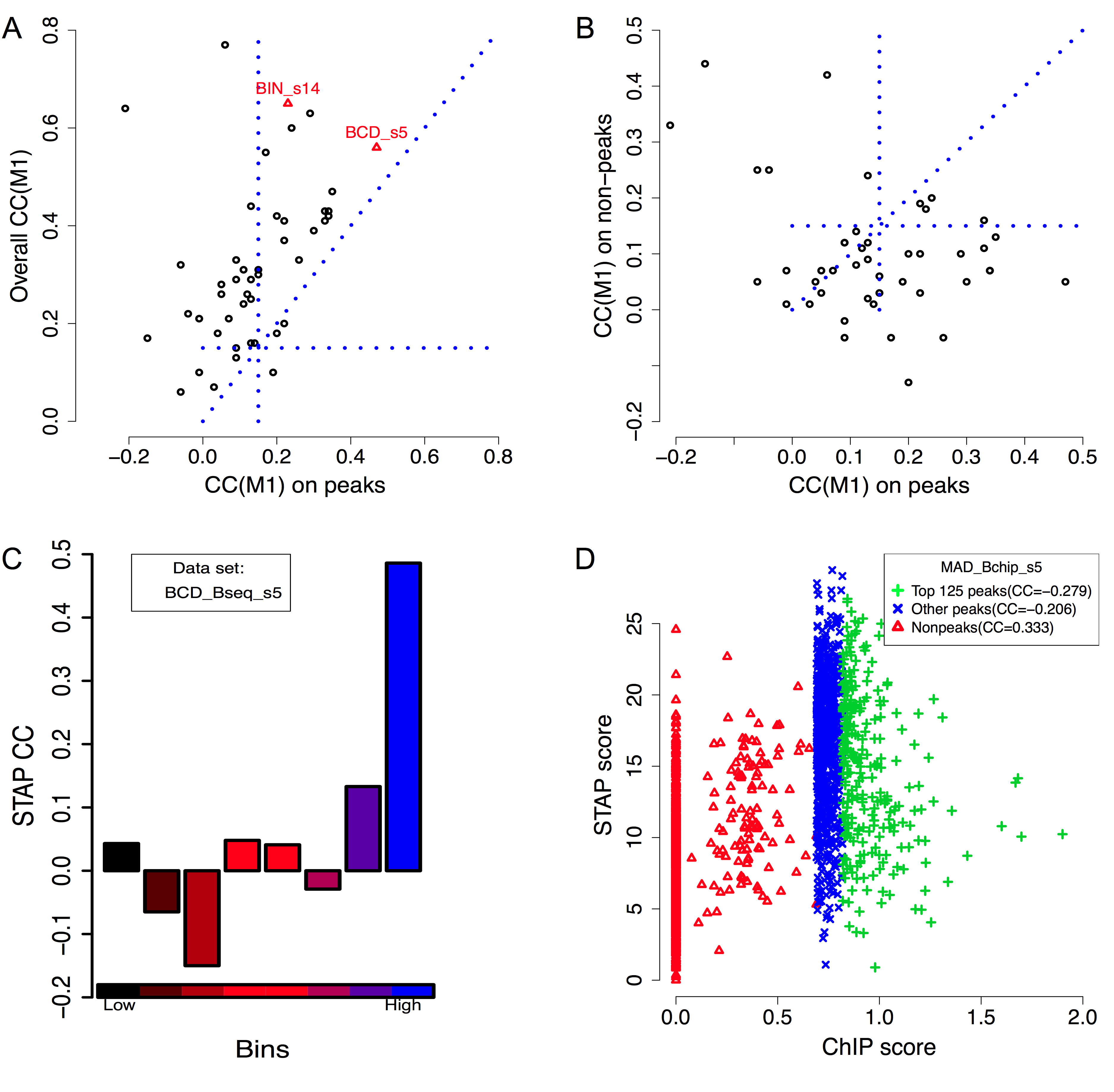

Supplement: Figure S5 — Contributions of peaks and non-peaks to the CC values reported for the single motif STAP model in Table 1. (Also see Table S10.) A. Comparison of model performance on peaks to the overall performance on the 45 data sets. Each point represents a data set. CC(M1) is the correlation coefficient of the baseline model driven by the primary motif M1. The horizontal and vertical dotted blue lines denote CC = 0.15, while the diagonal dotted blue line represents x = y in this chart. B. Comparison of model performance on peaks to the performance on non-peaks on the 45 data sets. C. Model performance may vary with the strength of the in vivo TF-DNA occupancy. The x-axis shows the 1000 peaks in the data set “BCD_Bseq_s5” divided into eight bins of 125 segments each based on ChIP scores. The top 125 most highly occupied genomic windows (rightmost bin) show the highest CC of 0.486 between in vivo occupancy and STAP prediction. The overall CC is 0.560, CC on all 1000 peaks taken together is 0.466, and CC on non-peaks is 0.050. D. Scatterplot of ChIP scores and STAP scores for all 2000 genomic windows in the data set “MAD_Bchip_s5”, where overall CC is 0.635, CC on peaks is −0.206, and CC on the 125 most highly occupied windows is −0.279. Green, blue and red points represent the 125 top ChIP peaks, 875 next highest ChIP peaks and 1000 randomly selected non-overlapping non-coding genomic windows respectively. (TIFF) [file pgen.1003571.s005.tiff]

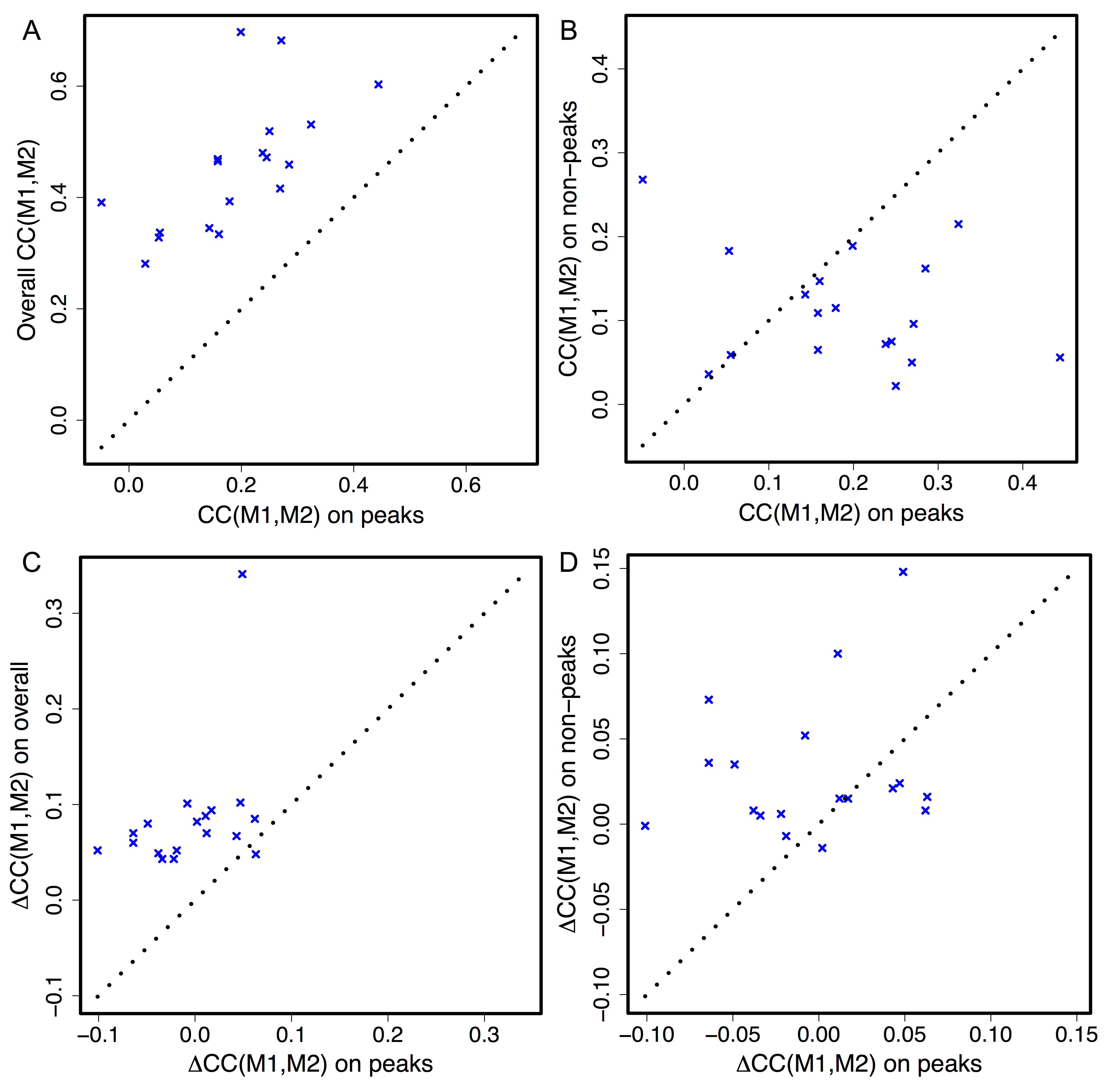

Supplement: Figure S6 — Effect of long-range cooperative interactions between pairs of TFs on the accuracy of modeling ChIP scores within peaks and non-peaks. We calculated the correlation coefficient (CC) between the in vivo occupancy and our STAP prediction on peaks and non-peaks for each of 18 TF-ChIP data sets from Table 2. (Also see Table S11.) A. Comparison of model performance on peaks to the overall performance on the 18 data sets. Each point represents a data set. CC(M1, M2) is the correlation coefficient of the cooperativity model driven by the primary motif M1 and the secondary motif M2. B. Comparison of model performance on peaks to the performance on non-peaks on these data sets. C. Comparison of the performance improvement on peaks to that on the entire data set. ΔCC (M1, M2) = CC(M1, M2) – CC(M1). D. Comparison of the performance improvement on peaks to that on non-peaks. (TIFF) [file pgen.1003571.s006.tiff]

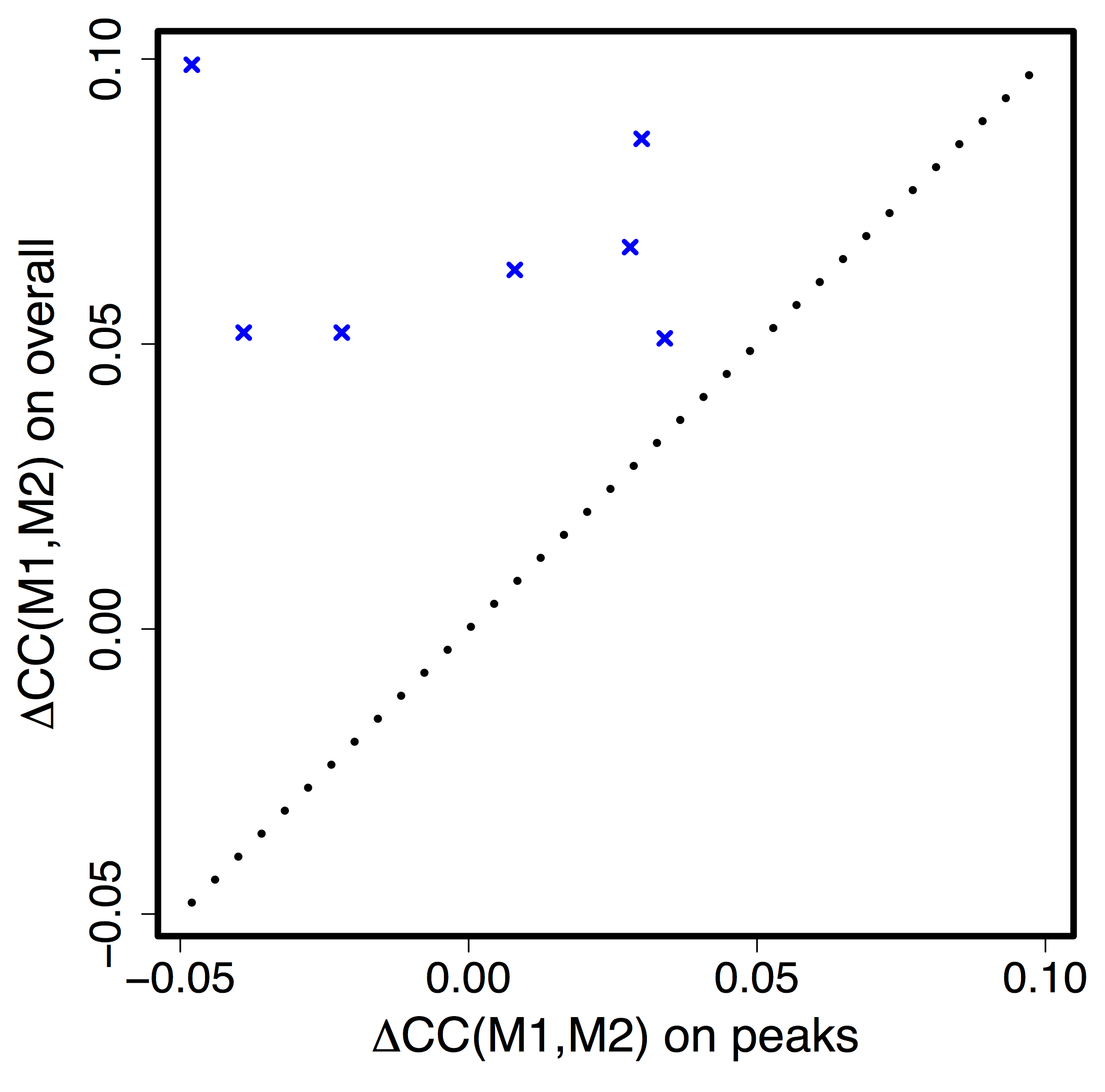

Supplement: Figure S7 — Effect of short-range cooperative interactions between pairs of TFs on the accuracy of modeling ChIP scores within peaks and non-peaks. We calculated the correlation coefficient (CC) between in vivo occupancy and our STAP prediction on peaks and non-peaks for seven TF-ChIP data sets where significant short-range TF-TF cooperativity has been identified (refer to Table 3 and Table S12). Shown is a comparison of performance improvement on peaks to that on the entire data set. Each point represents a data set. CC(M1, M2) is the correlation coefficient of the cooperativity model with primary motif M1 and secondary motif M2. ΔCC(M1, M2) = CC(M1, M2) – CC(M1). (TIFF) [file pgen.1003571.s007.tiff]

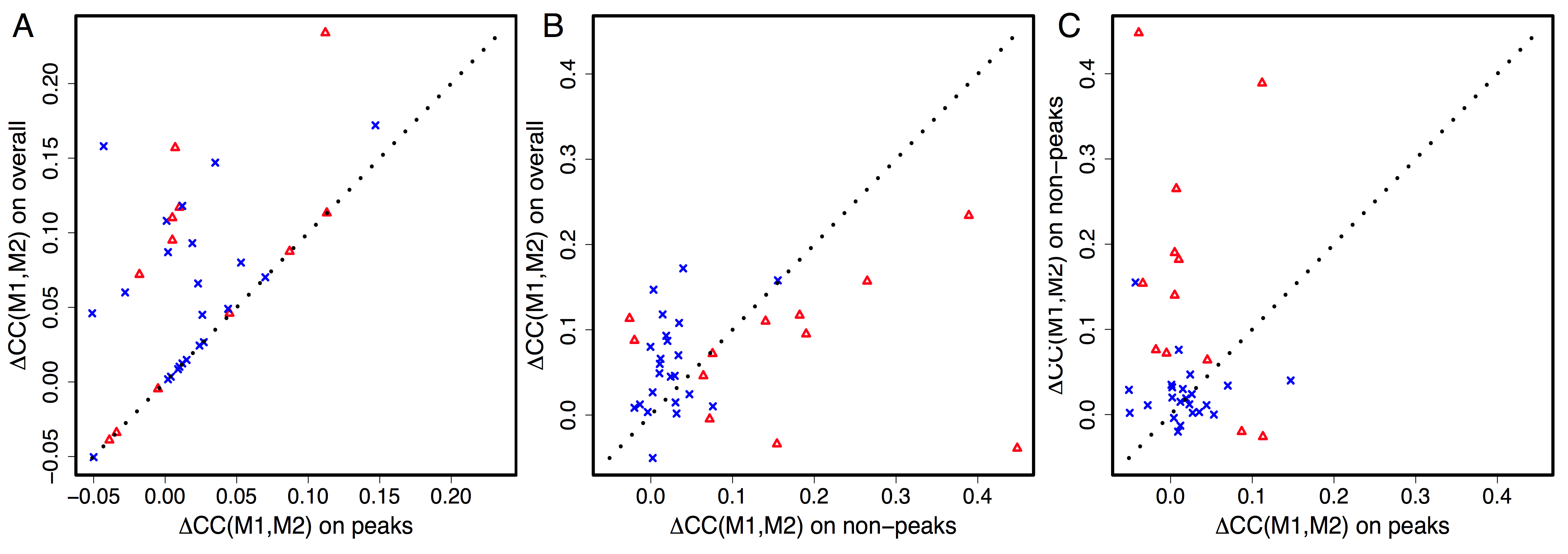

Supplement: Figure S8 — Effect of antagonistic interactions between pairs of TFs on the accuracy of modeling ChIP data, for peaks and non-peaks separately. We calculated the correlation coefficient (CC) between in vivo occupancy and our STAP prediction on peaks and non-peaks for 35 cases of antagonism where significant TF-TF antagonism was identified in Table 5. (Also see Table S13.) A. Comparison of the performance improvement on peaks with that on the entire data set. Each point represents a data set. CC(M1, M2) is the correlation coefficient of the antagonism model with primary motif M1 and secondary motif M2. ΔCC (M1, M2) = CC(M1, M2) – CC(M1). B. Similar to (A), except that the x axis represents ΔCC(M1,M2) on non-peaks rather than peaks. C. Comparison of the performance improvement on peaks to that on non-peaks. Red symbols in both panels represent cases where the improvement on either peaks or non-peaks is larger than the improvement on the entire data set. Blue symbols represent all other data sets. (TIFF) [file pgen.1003571.s008.tiff]

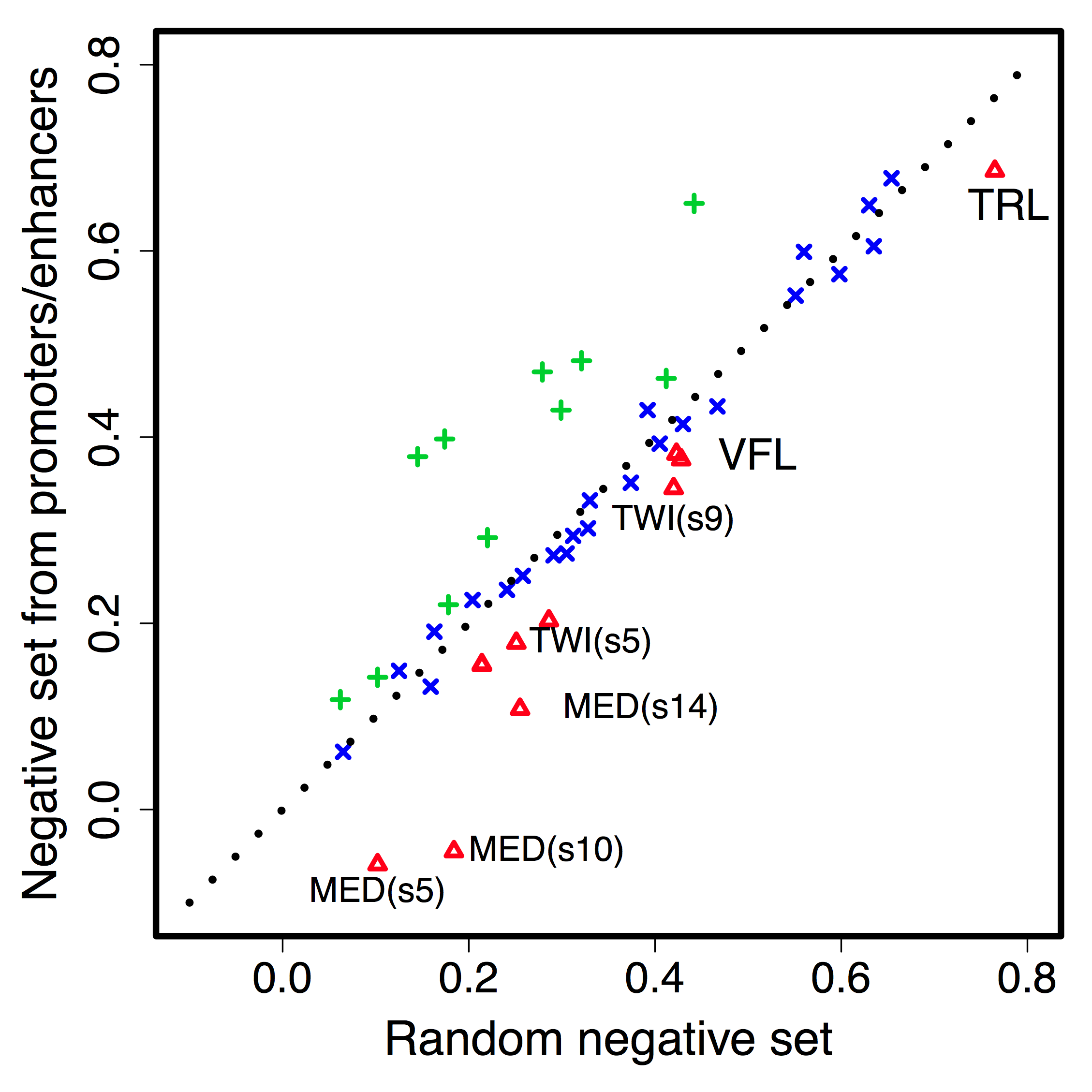

Supplement: Figure S9 — Performance of the baseline (single motif) model using two different definitions of the “negative set.” By default each TF-ChIP data set comprised the top 1000 peaks and 1000 random non-coding sequence windows (non-peaks). All results in the main text are based on this definition of non-peaks. Here, we replaced the 1000 randomly chosen non-peaks with 1000 randomly chosen non-peaks that happen to be ChIP peaks of a different TF. The plot shows CC values of the STAP model on each data set, using these two definitions of non-peaks (x-axis corresponds to the default definition). Red symbols represent data sets where the CC with the default definition of non-peaks is better than the CC with the new definition of non-peaks (by 0.04 or more). Green symbols represent data sets where the CC with the new definition is better than the CC with the default definition of non-peaks (by 0.04 or more). (Also refer to Table S15.) (TIFF) [file pgen.1003571.s009.tiff]

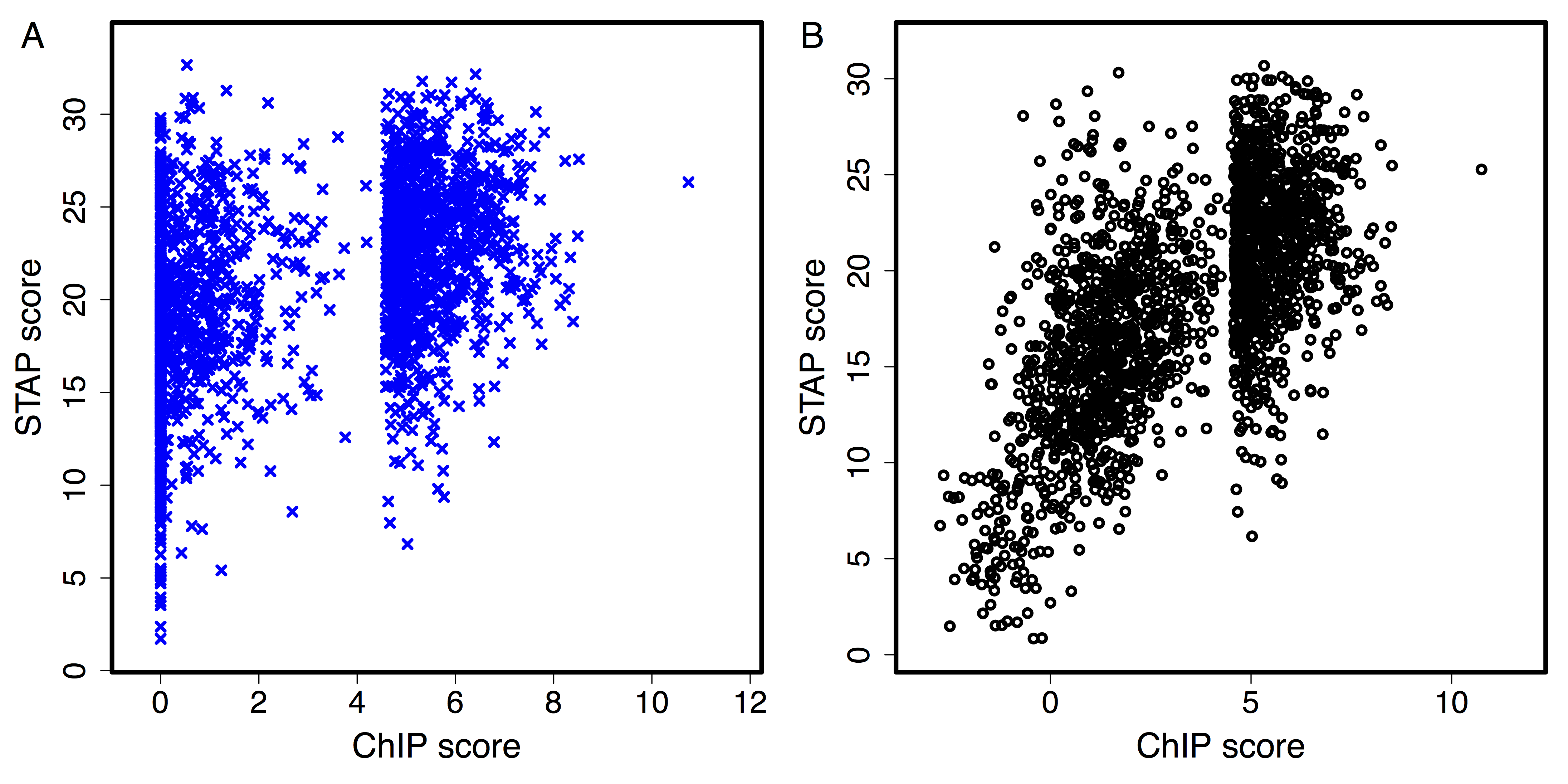

Supplement: Figure S10 — Detailed examination of ChIP scores and STAP scores on data set UBX_Mchip_s5_14, which shows a pronounced increase in CC when random non-peaks in the data set are replaced by non-peaks randomly chosen from peaks of other data sets. A. In-vivo TF-DNA occupancy versus STAP baseline model prediction on 2000 genomic windows in the default data set that includes 1000 non-overlapping non-exonic non-peaks extracted randomly from the whole genome. B. In-vivo TF-DNA occupancy versus STAP baseline model prediction on 2000 genomic windows that includes 1000 non-peaks of the TF chosen randomly from peaks of other data sets (corresponding to other TFs). (TIFF) [file pgen.1003571.s010.tiff]

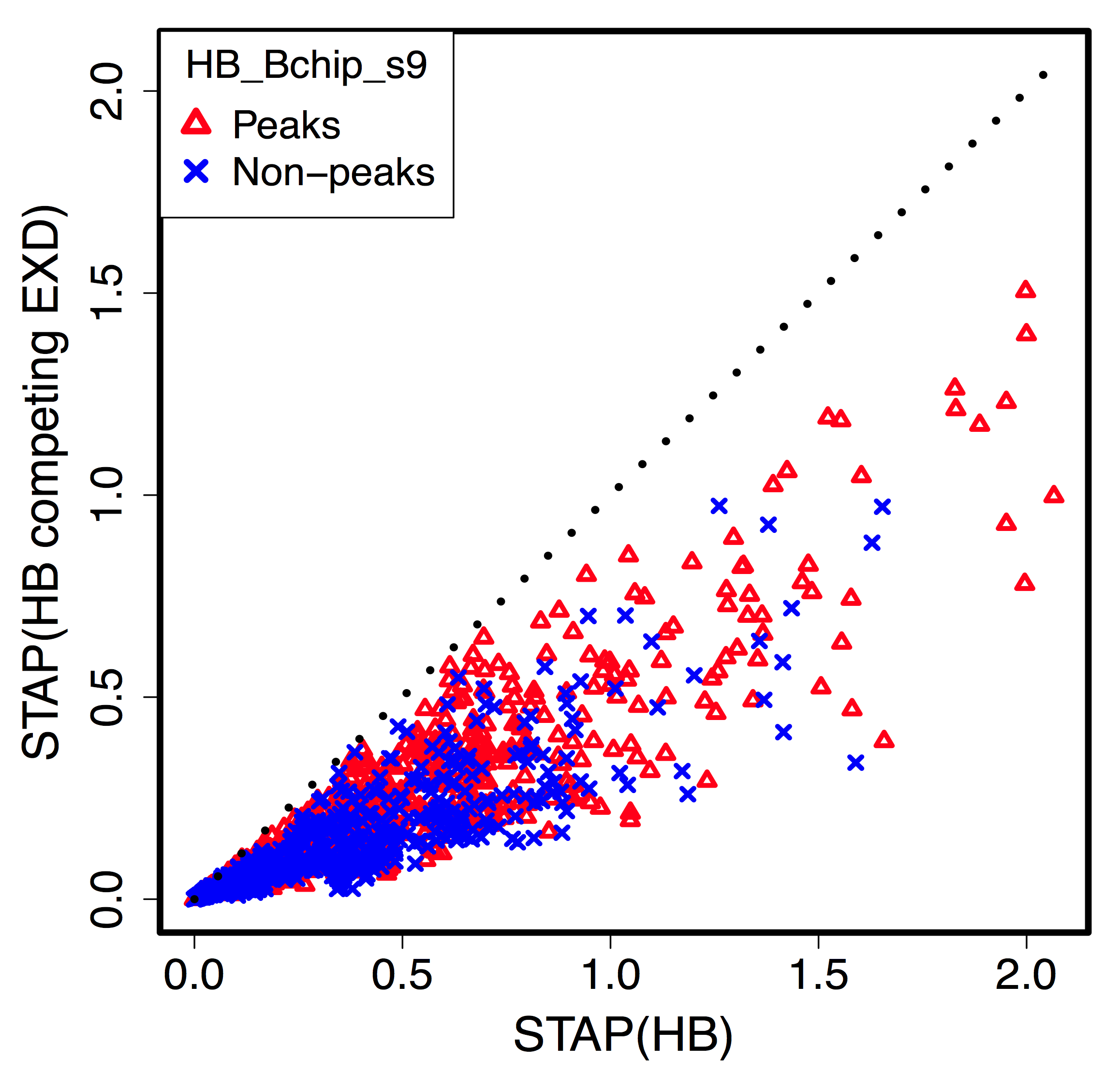

Supplement: Figure S11 — Influence of the competing DNA binding of HB and EXD to the TF-DNA occupancy prediction for the data set HB_Bchip_S9. We trained the STAP baseline model (HB only) without 4-fold cross validation and obtained HB's binding weight parameter value γHB. Then we fit the STAP competition model by fixing the binding weight parameter value of HB as γHB and setting the binding weight parameter of EXD as the only free parameter. The scatter plot shows the predicted TF-DNA occupancy score of each sequence (peak and non-peak) as per the baseline model with primary TF only and the advanced model that includes competitive binding by EXD. From these plots, we note that many peaks (red) as well as non-peaks (blue) fall below the diagonal, which represents a lower STAP score from the advanced model (with competition) than from the baseline model (without competition). This suggests that the advanced model, to its advantage, in both peaks and non-peaks, is exploiting overlapping sites. (TIFF) [file pgen.1003571.s011.tiff]

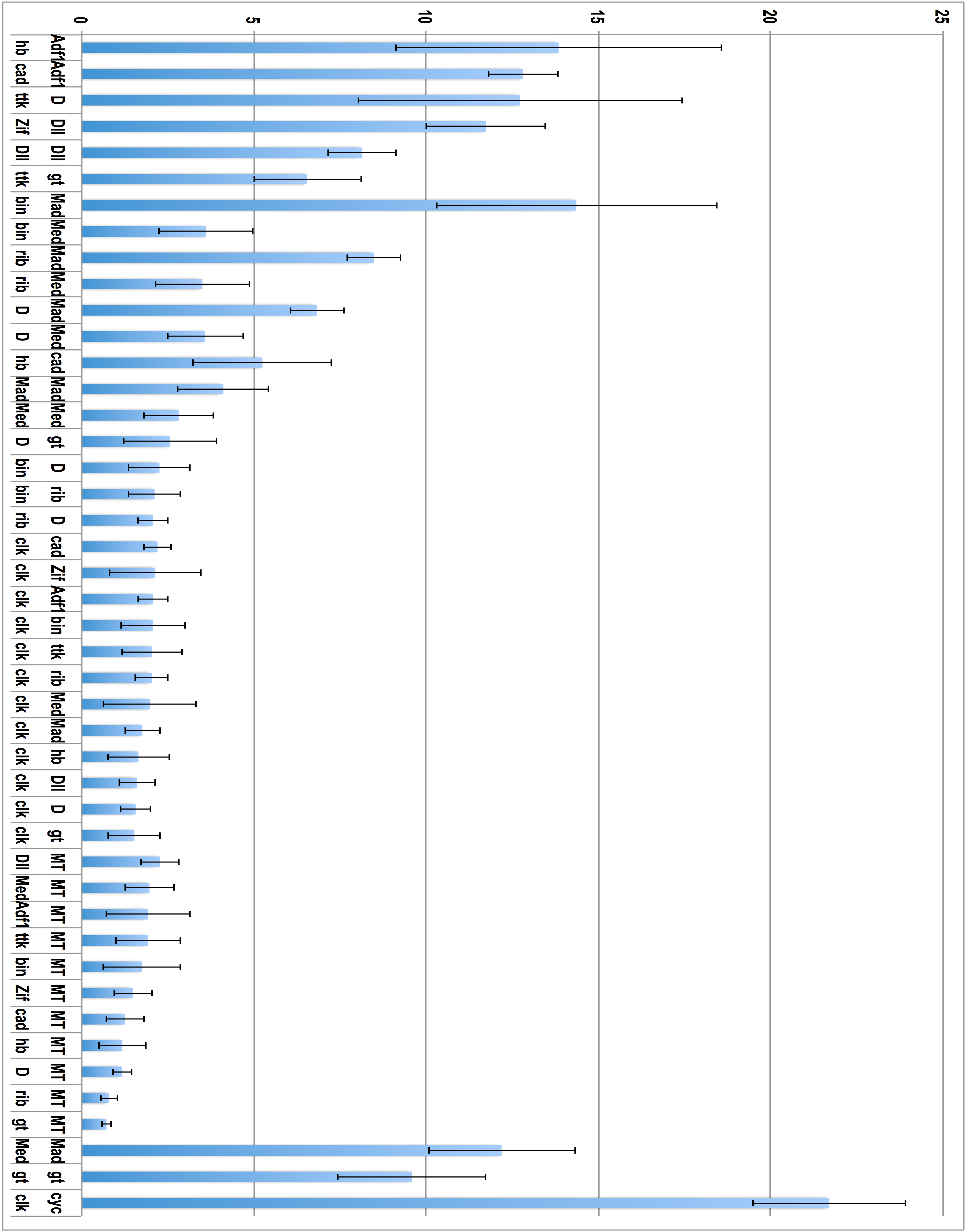

Supplement: Figure S12 — Experimental validation of predicted direct TF-TF interactions. This chart is an extended version of Figure 4C (right panel) but with additional negative controls with either CLK or empty vector (MT). GT-GT, MAD-MED and CLK-CYC positive controls are shown. The same chart also appears in Supplementary Table S9 (Excel file, worksheet named “heterodimers + more controls”). (TIFF) [file pgen.1003571.s012.tiff]

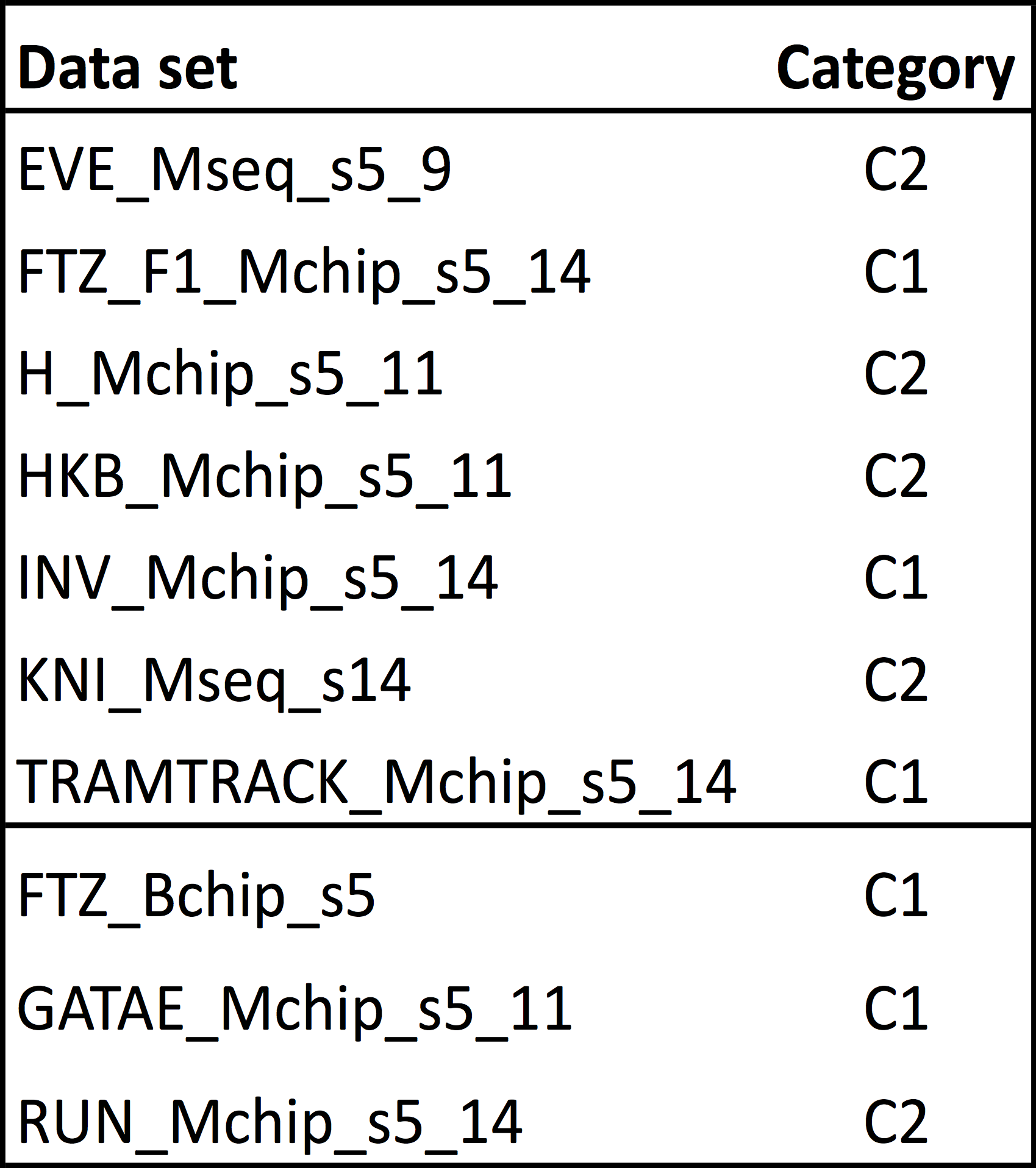

Supplement: Table S1 — Ten data sets excluded from detailed analysis. These include seven data sets for which no model was able to achieve CC above the chosen threshold (≥0.15) (top seven rows). FTZ_Bchip_s5 and RUN_Mchip_s5_14 are excluded due to the negative association between ChIP profile and the estimated TF-DNA occupancy in the single-motif baseline model. GATAE_Mchip_s5_11 is also disregarded since 1) the parameter values learned from different folds of the cross-validation experiment were widely different, and 2) the learned parameter values were sensitive to the site threshold used in STAP. We categorized these “failed” data sets into two classes: C1 = only one data set was examined for this TF, so both the model and data set quality are suspect; C2 = multiple data sets were examined for this TF (from different sources and/or developmental stages) and at least one data set shows a CC ≥0.15, suggesting that this failed data set is suspect rather than the model. (TIFF) [file pgen.1003571.s013.tiff]

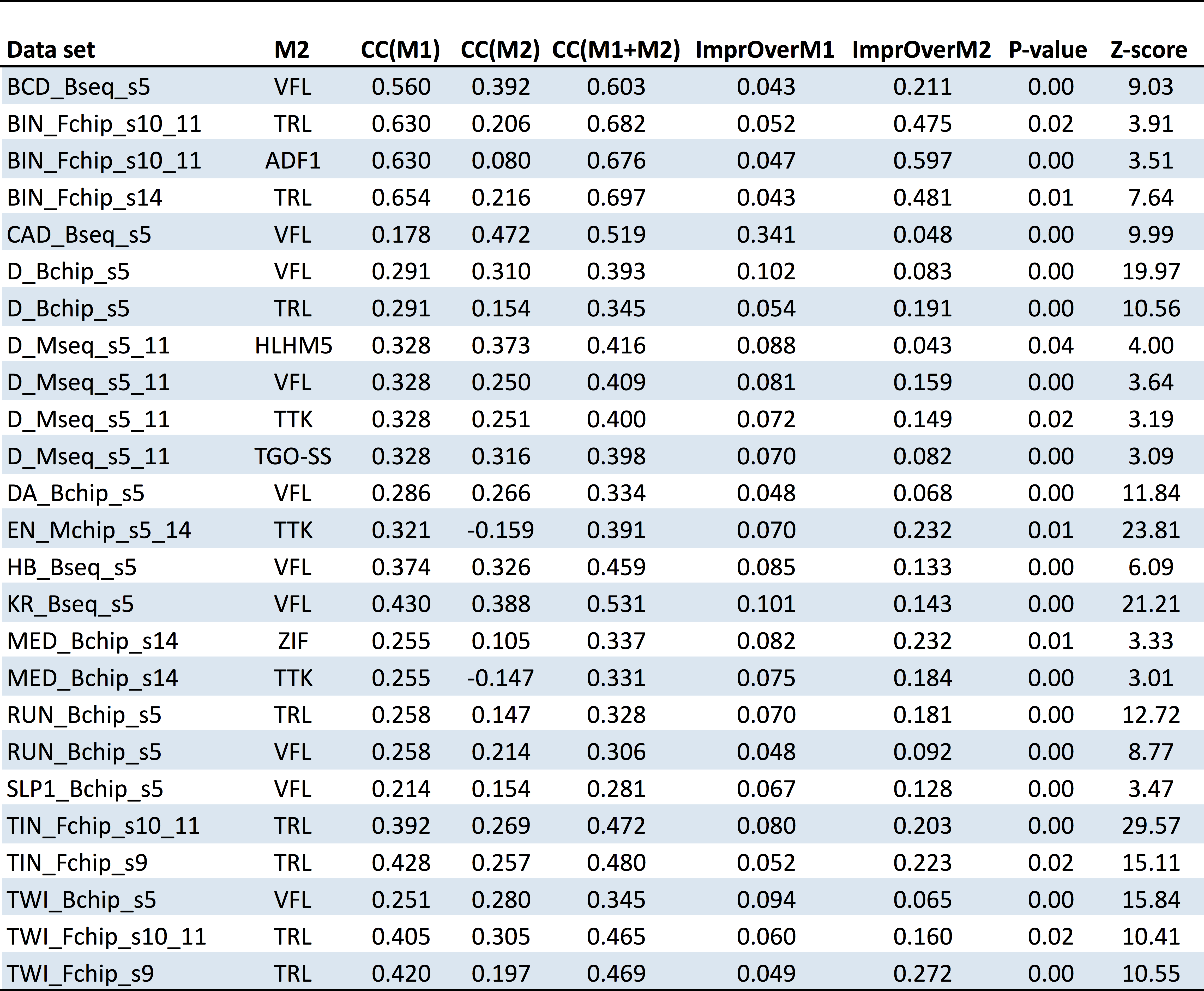

Supplement: Table S2 — Effect of cooperative interactions between pairs of TFs on the accuracy of modeling ChIP data. Shown here are all cases where P-value is < = 0.05 and Z-score is > = 3 at distance threshold of 150 bps. Column semantics are as in Table 2 of the main text. (TIFF) [file pgen.1003571.s014.tiff]

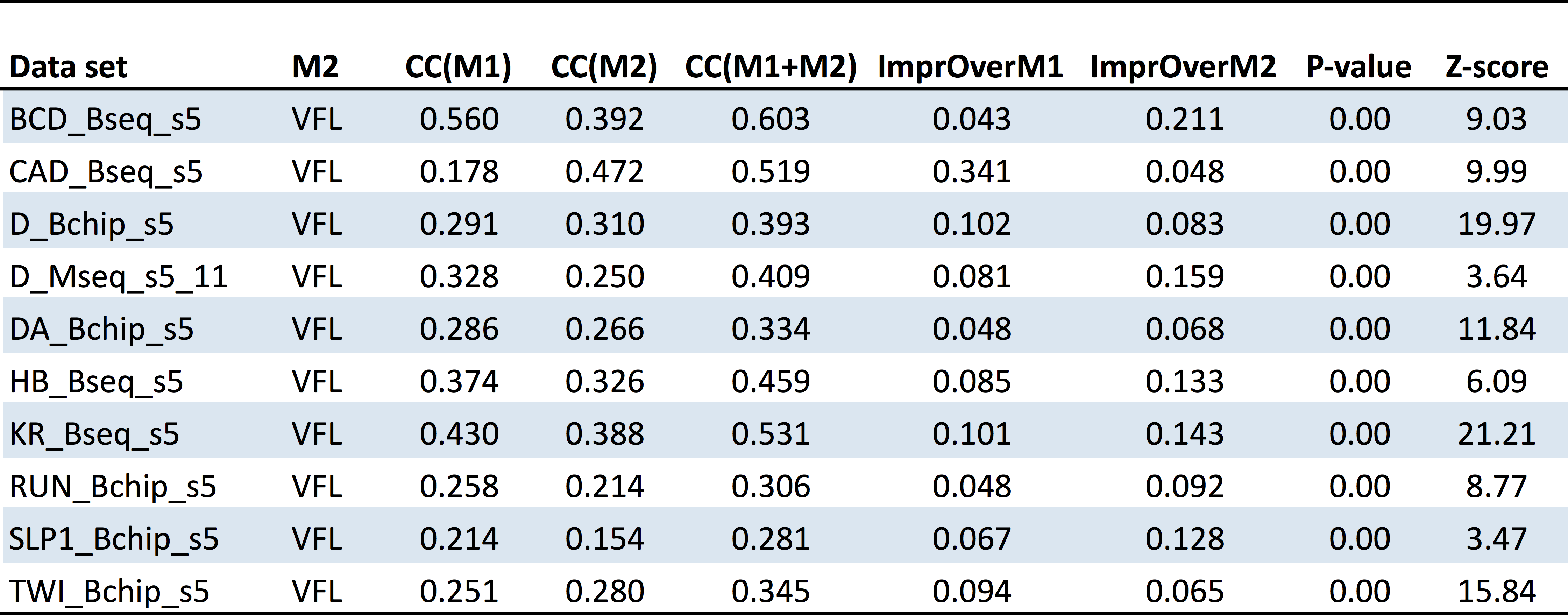

Supplement: Table S3 — Cases of significant influence of VFL, in cooperativity mode with distance threshold = 150 bp. Column semantics are as in Table 2 of the main text. A ‘-’ indicates that the effect was insignificant. (TIFF) [file pgen.1003571.s015.tiff]

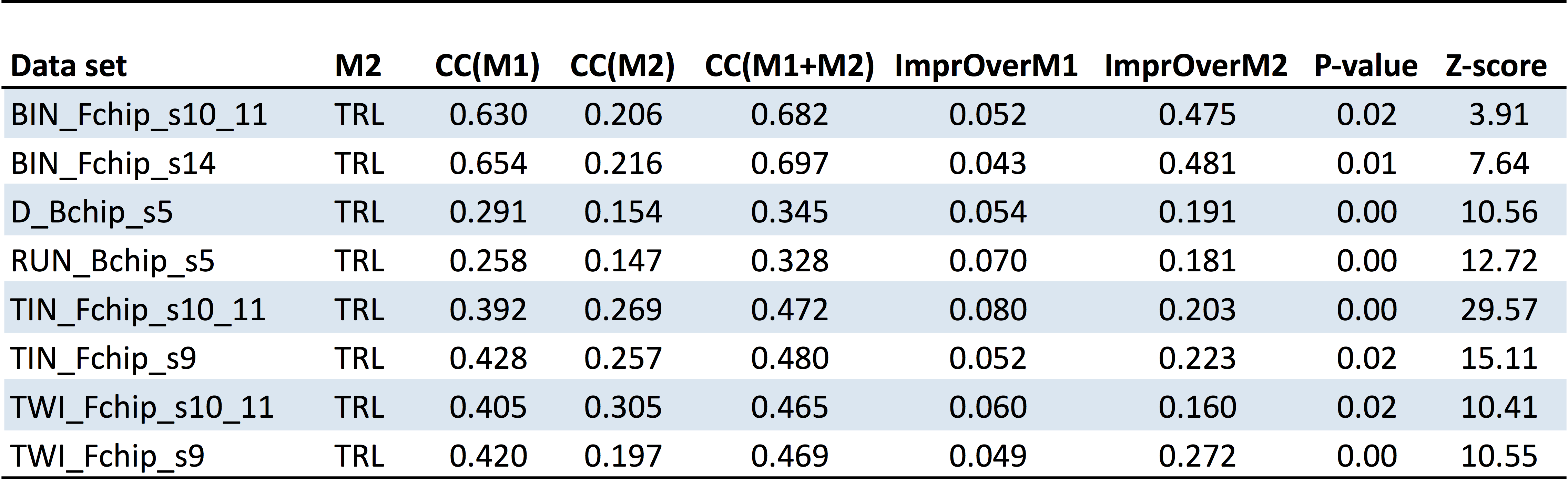

Supplement: Table S4 — Cases of significant influence of TRL, in cooperativity model with distance threshold equal to 150 bp. Column semantics are as in Table 2 of the main text. A ‘-’ indicates that the effect was insignificant. (TIFF) [file pgen.1003571.s016.tiff]

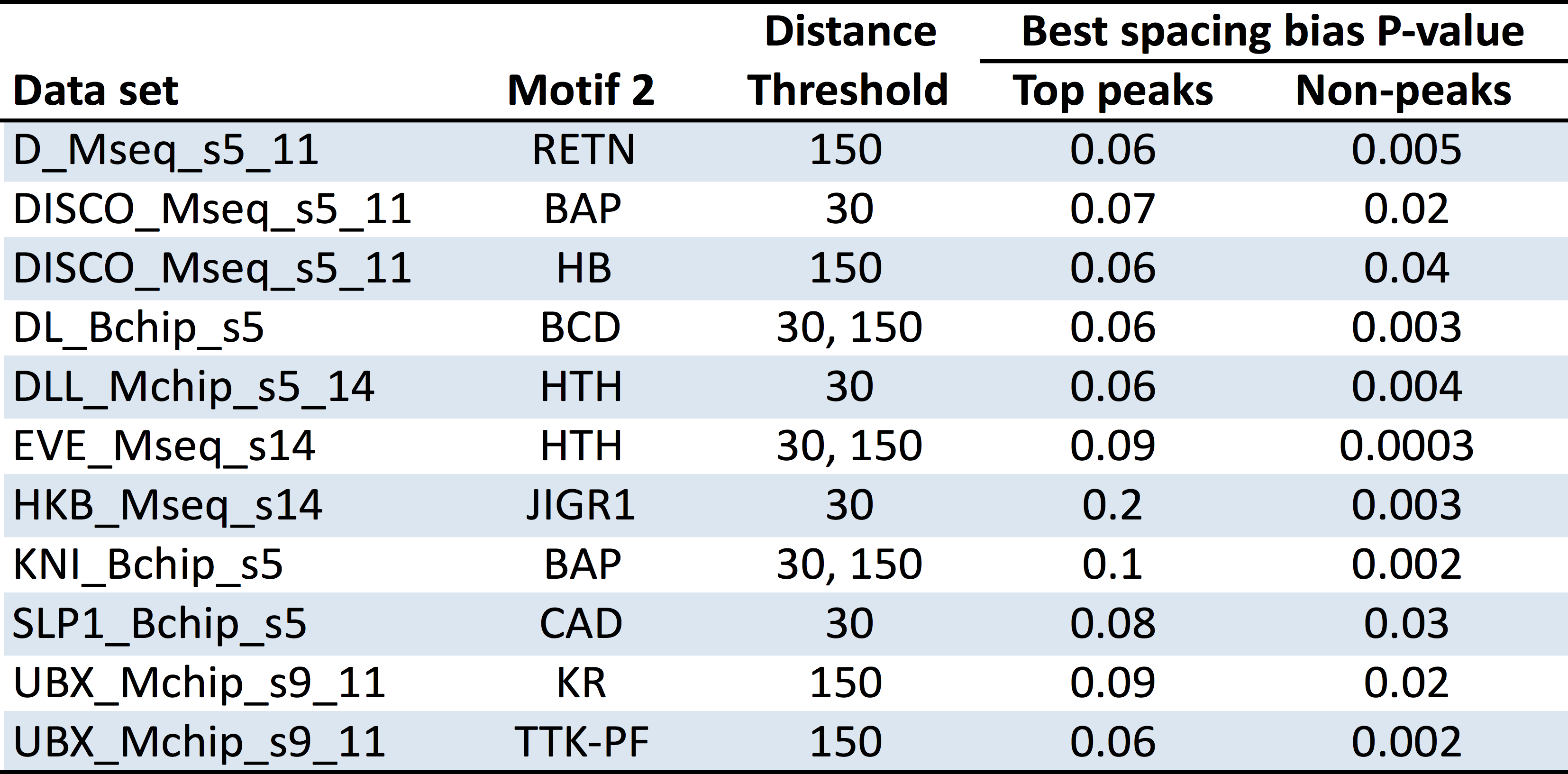

Supplement: Table S5 — Spacing bias analysis for antagonistic influences where the bias is significant in the non-peaks and not in peaks. Shown are cases where the STAP model, using the primary motif and the secondary motif listed in column “Motif 2”, with antagonistic interaction at distance less than “Distance threshold”, led to significant improvement in CC on a data set. In each case, we tested for a bias for a spacing range [1–2, 2–3, 3–4, …, 29–30], in peaks and non-peaks separately, and report the lowest p-value observed across all spacing ranges. (TIFF) [file pgen.1003571.s017.tiff]

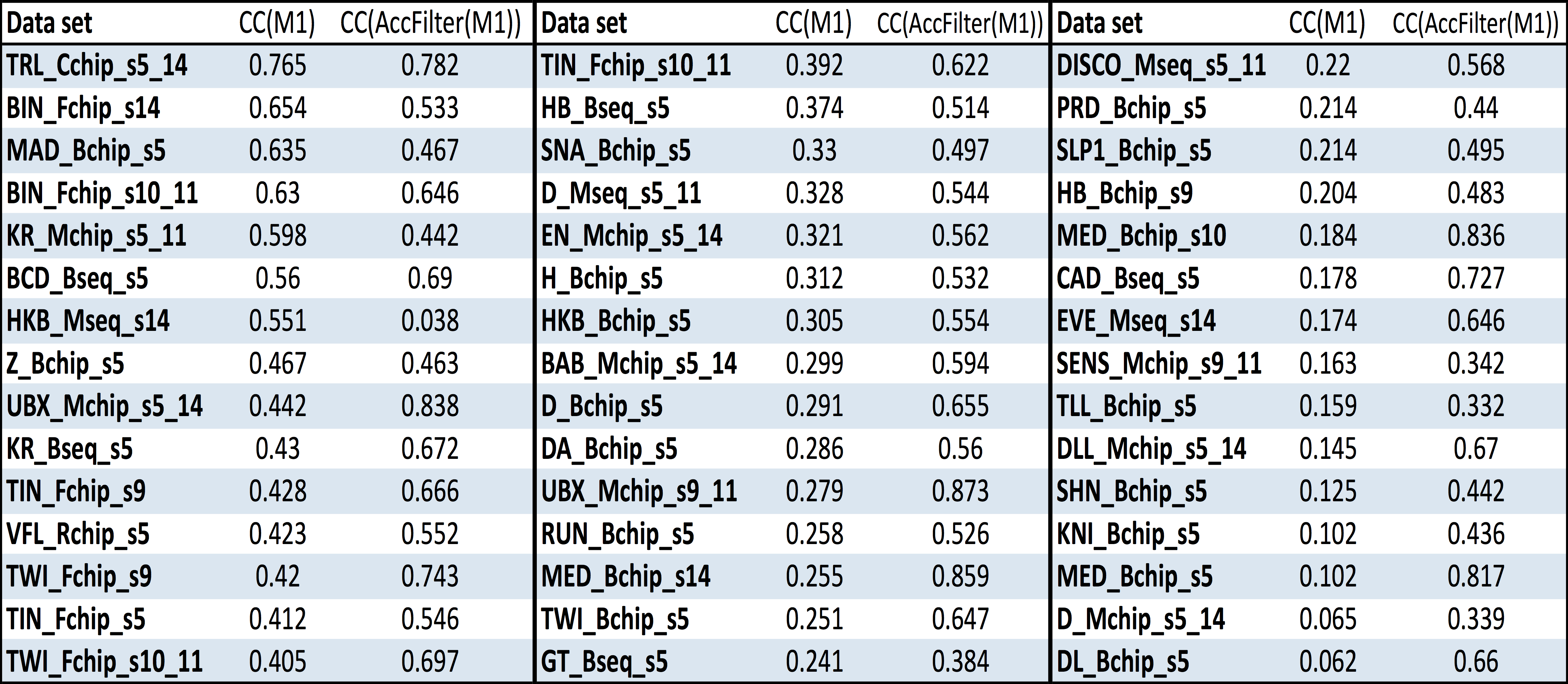

Supplement: Table S6 — Correlation coefficient between ChIP scores and each of two different computational scores: STAP predictions using the primary motif (CC(M1)) and STAP predictions using the primary motif but set to zero in inaccessible regions (CC(AccFilter(M1))). (TIFF) [file pgen.1003571.s018.tiff]

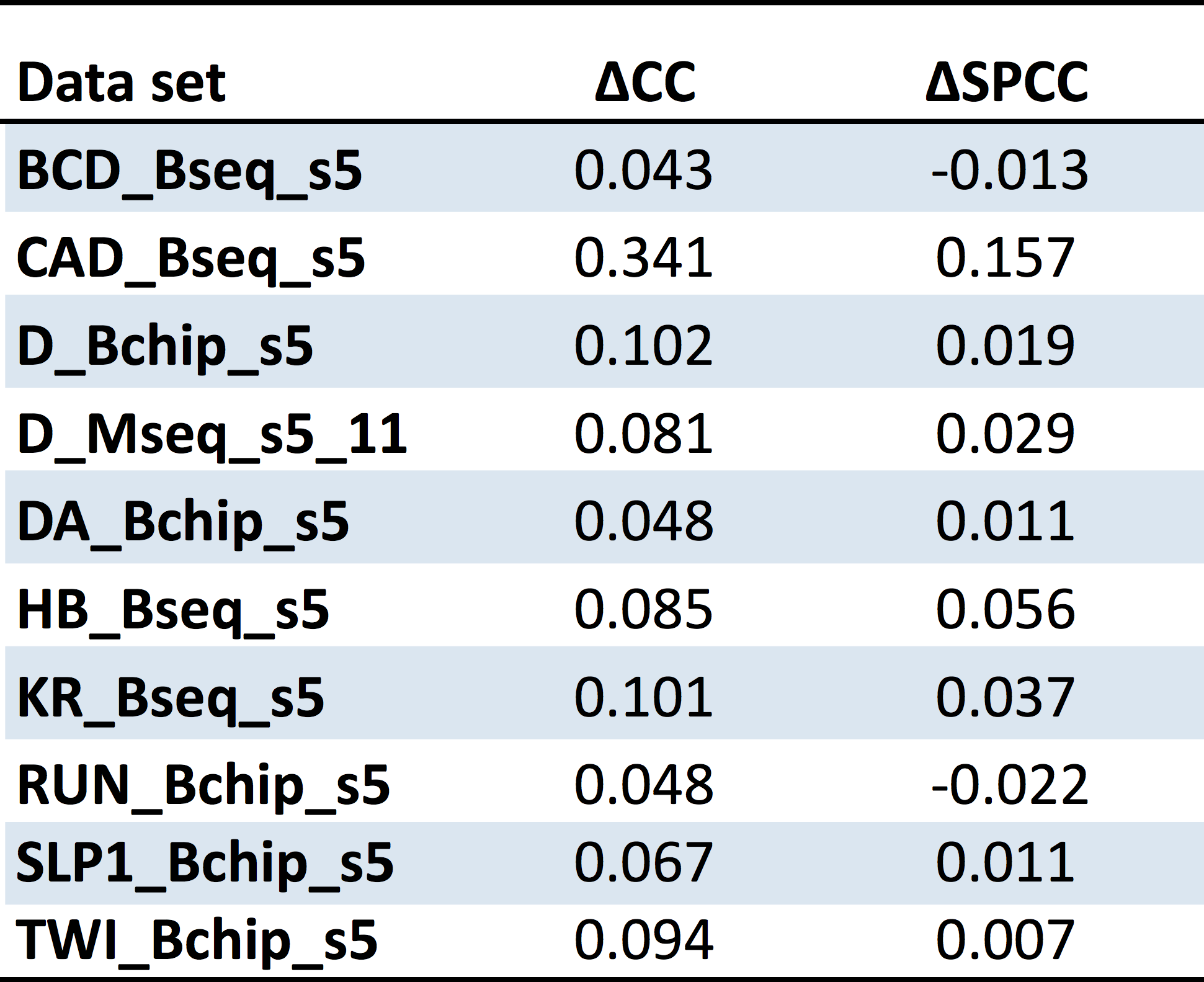

Supplement: Table S7 — Effect of VFL before (ΔCC) and after (ΔSPCC) partialing out accessibility. A substantial effect (≥0.04) remains even after partialing out accessibility for the data sets CAD_Bseq_s5 and HB_Bseq_s5, while a borderline significant effect remains for the data set KR_Bseq_s5. (TIFF) [file pgen.1003571.s019.tiff]

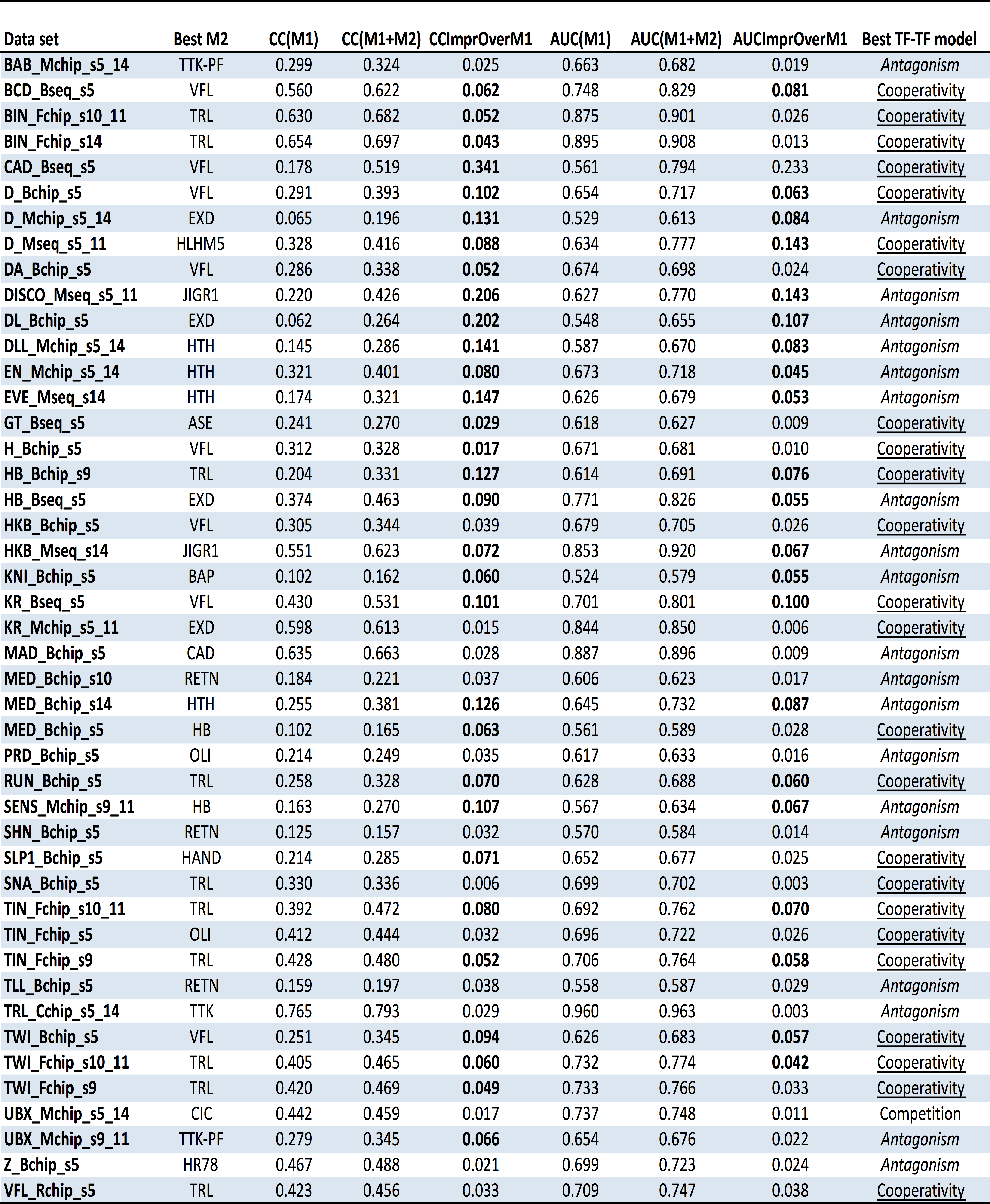

Supplement: Table S8 — The CC and AUC scores of the baseline model and best TF-TF interaction model of 45 amenable data set. CCImprOverM1 is the difference of CC(M1+M2) and CC(M1), where M2 is a best secondary motif candidate under the most beneficial TF-TF interaction model. AUCImprOverM1 is the corresponding AUC difference under these two models. Bold font in the two columns refers to cases where the improvement arising from the best model is > = 0.04. (TIFF) [file pgen.1003571.s020.tiff]

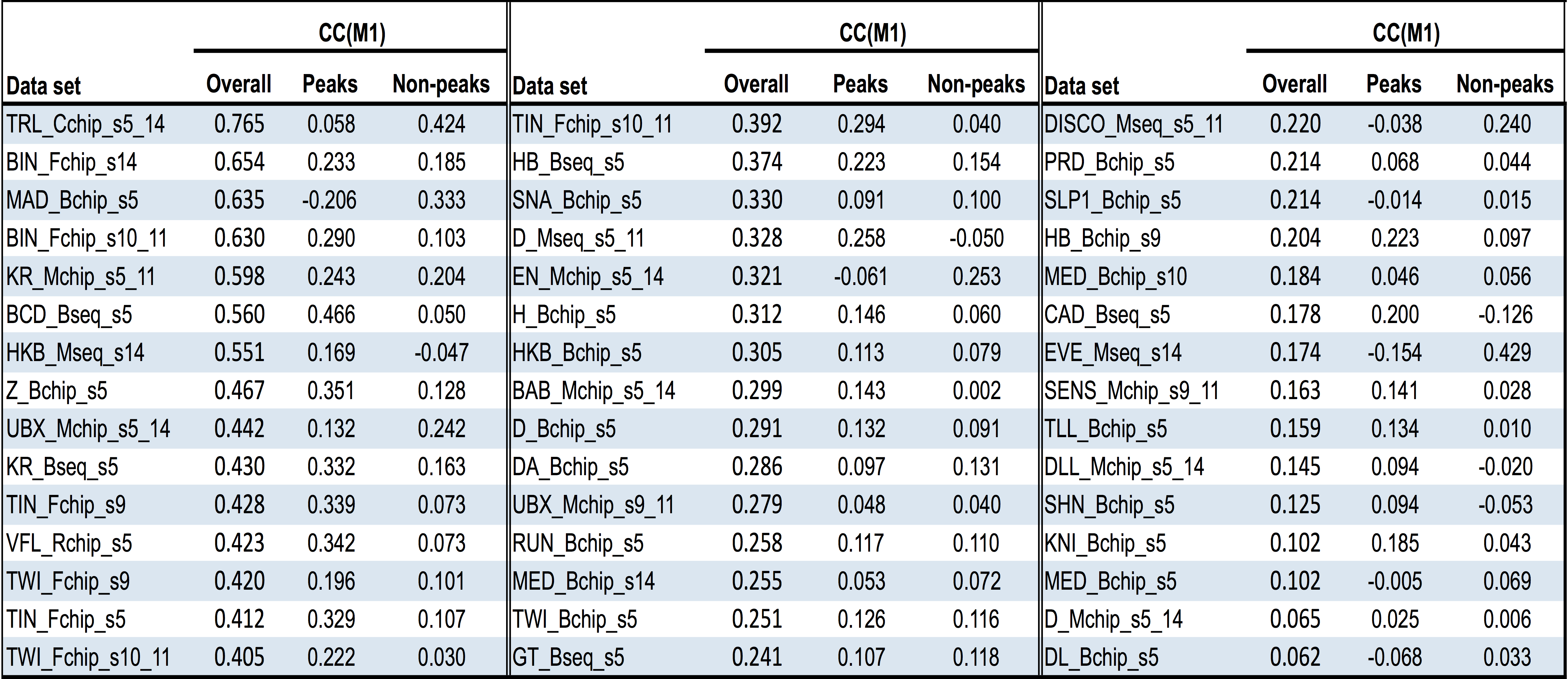

Supplement: Table S10 — Evaluation of single motif STAP model on peaks and non-peaks of 45 TF-ChIP data sets (refer to Table 1). The “Overall” column is identical to the column labeled “CC(M1)” in Table 1. (TIFF) [file pgen.1003571.s022.tiff]

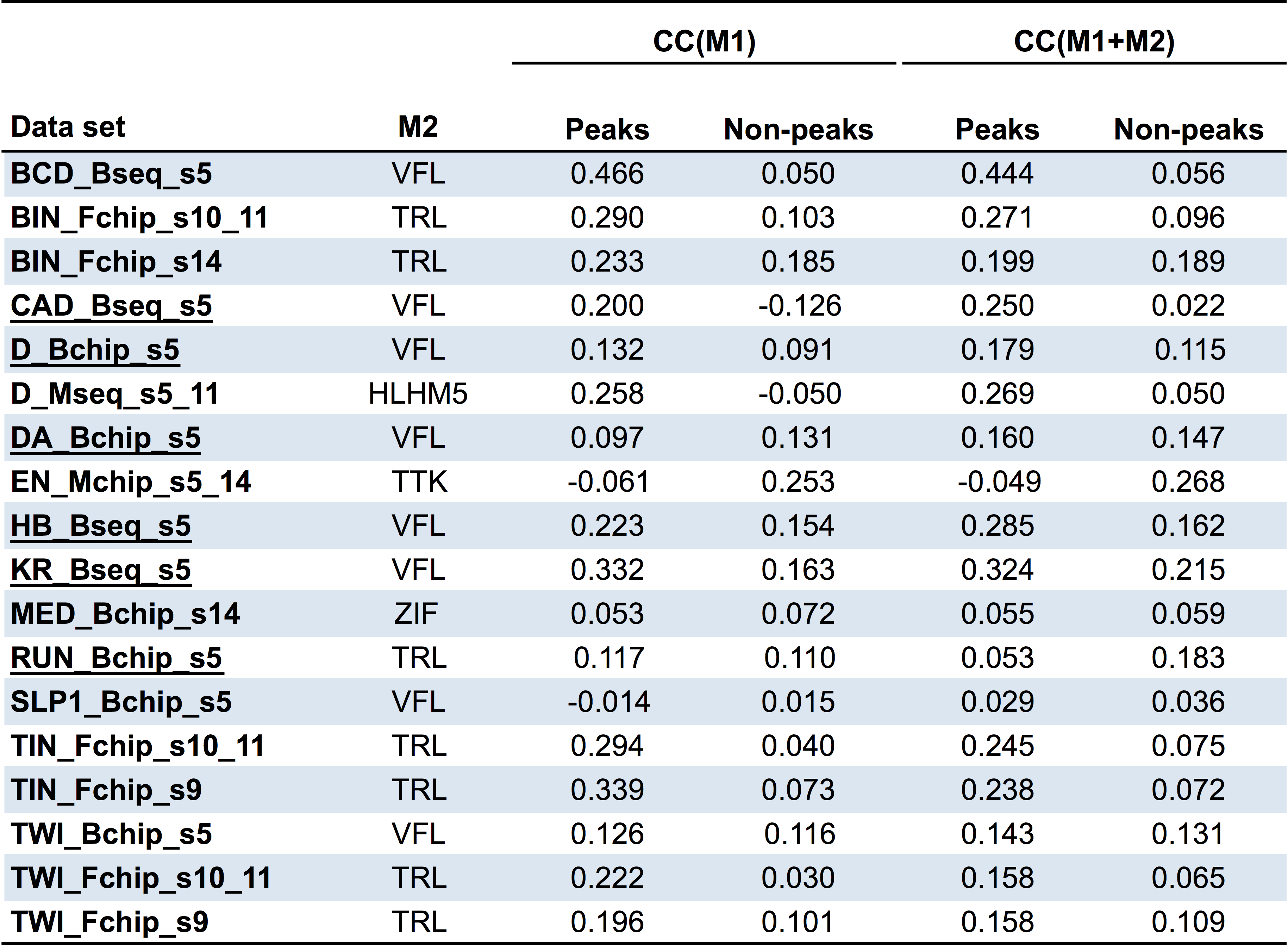

Supplement: Table S11 — Effect of long-range cooperative interactions between pairs of TFs on the accuracy of modeling ChIP scores within peaks and non-peaks (refer to Table 2). Rows where the data set name is in underlined font represent cases where the ΔCC on peaks or non-peaks is ≥0.04, and the corresponding CC(M1+M2) is ≥0.15. (TIFF) [file pgen.1003571.s023.tiff]

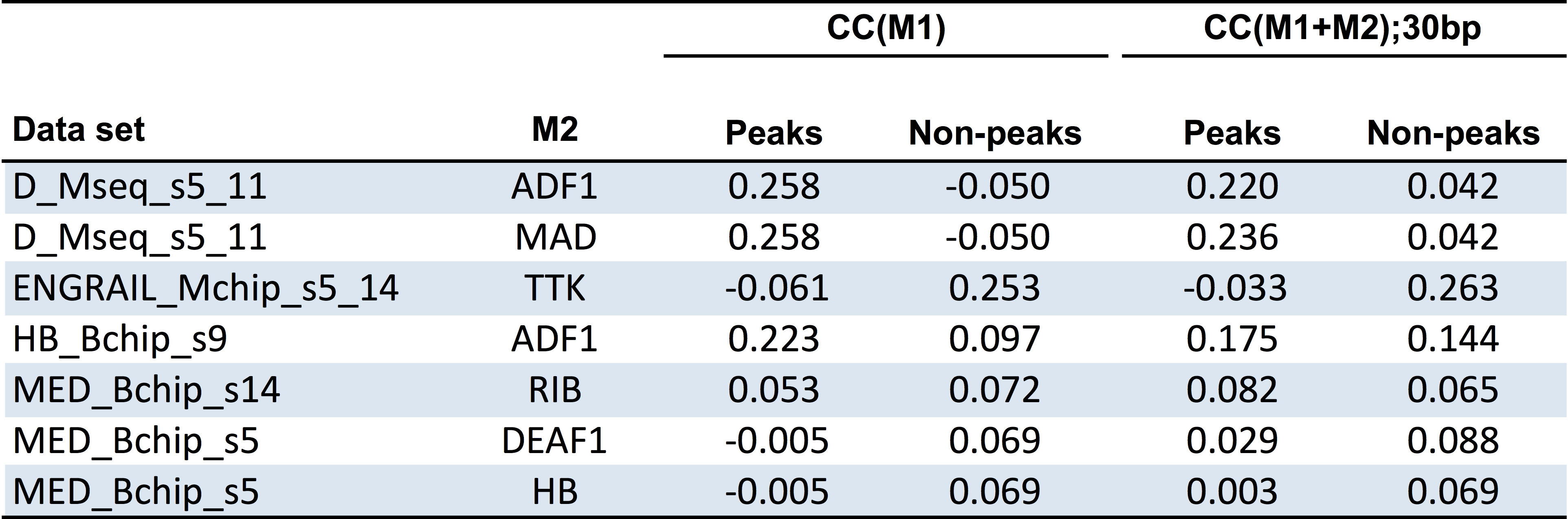

Supplement: Table S12 — Effect of short-range cooperative interactions between pairs of TFs on the accuracy of modeling ChIP scores within peaks and non-peaks (refer to Table 3). These cases correspond to the rows of Table 3 that are significant at distance threshold 30 bp and marked with asterisks. In none of these seven cases does ΔCC within peaks or within non-peaks rise above the nominal threshold of 0.04, suggesting that the observed improvements due to short-range cooperativity modeling (Table 3) arise mainly from a better discrimination of peaks from non-peaks. (TIFF) [file pgen.1003571.s024.tiff]

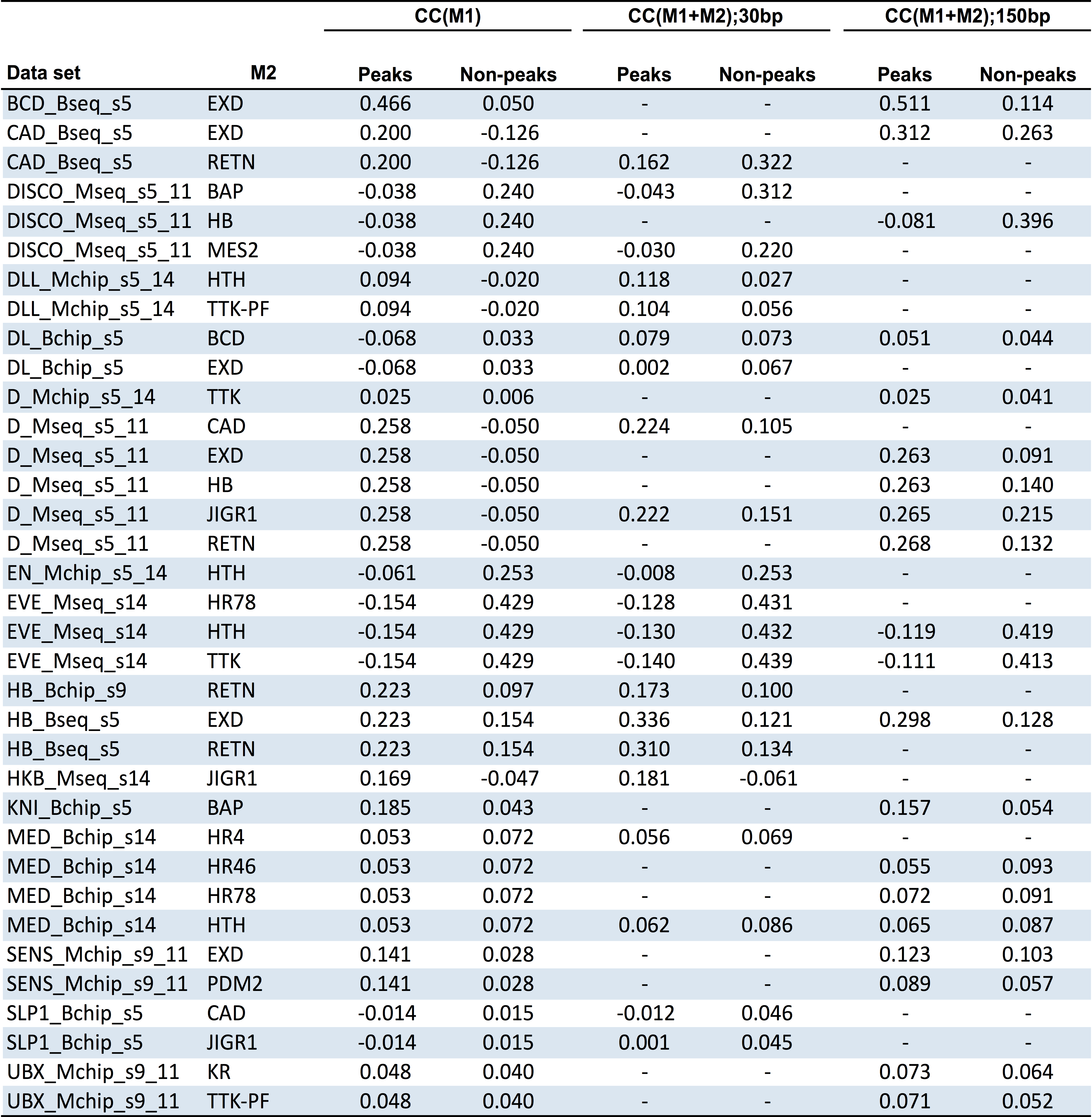

Supplement: Table S13 — Effect of antagonistic interactions between pairs of TFs on the accuracy of modeling ChIP data, for peaks and non-peaks separately (refer to Table 5). (TIFF) [file pgen.1003571.s025.tiff]

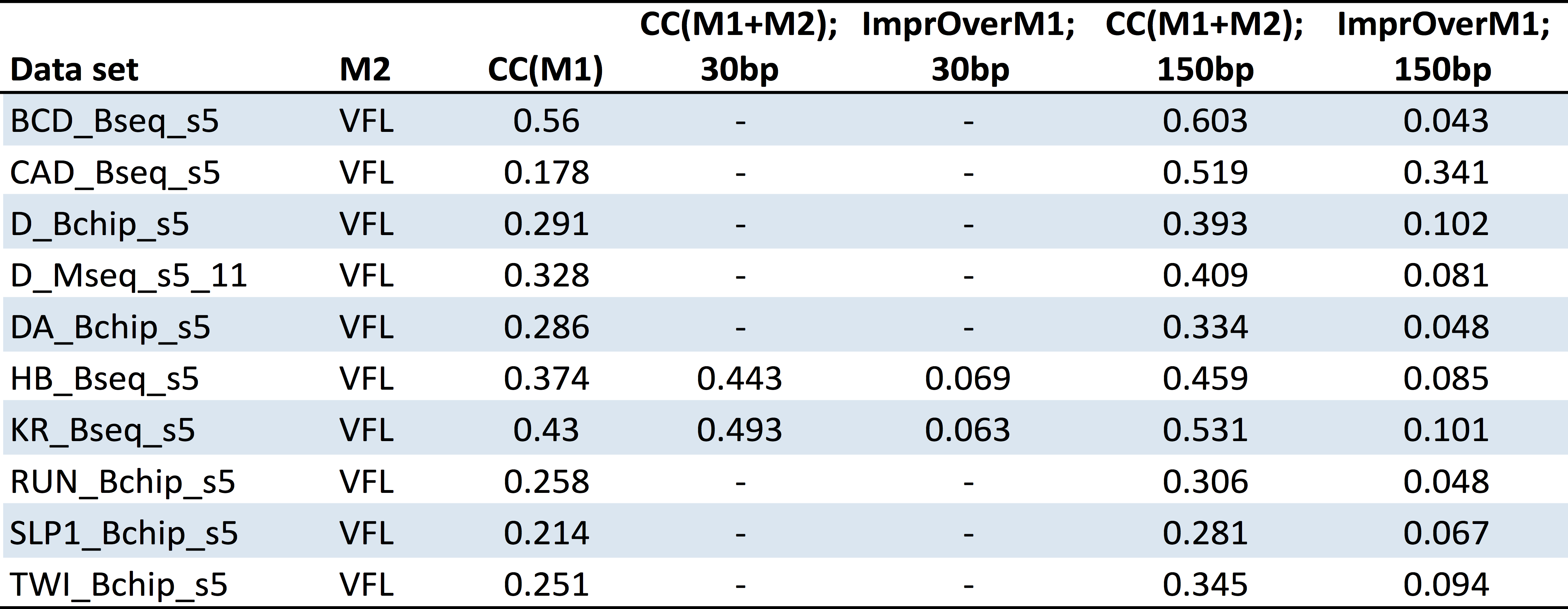

Supplement: Table S17 — All cases where VFL has a significant cooperativity effect at distance threshold = 30 bp or 150 bp, showing the effect at both distance thresholds. Column semantics are as in Table 3 of the main text. A ‘-’ indicates that the effect was insignificant. (TIFF) [file pgen.1003571.s029.tiff]

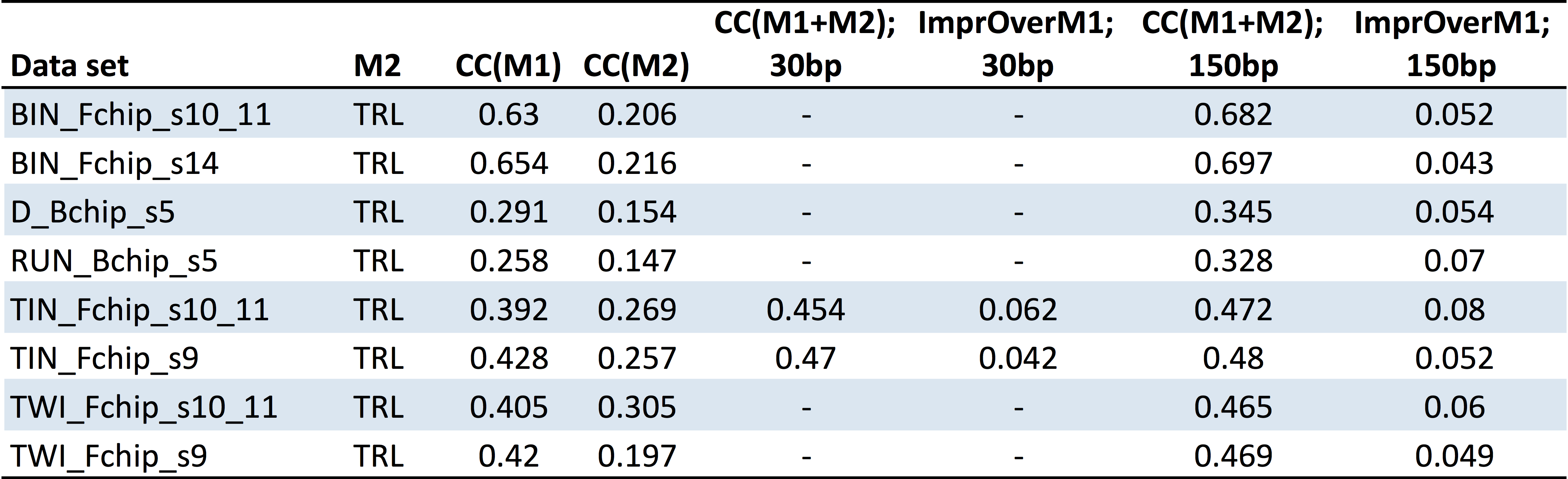

Supplement: Table S18 — All cases where TRL has a significant effect at distance threshold = either 30 bp or 150 bp, showing the effect at both distance thresholds. Column semantics are as in Table 3 of the main text. A ‘-’ indicates that the effect was insignificant. (TIFF) [file pgen.1003571.s030.tiff]

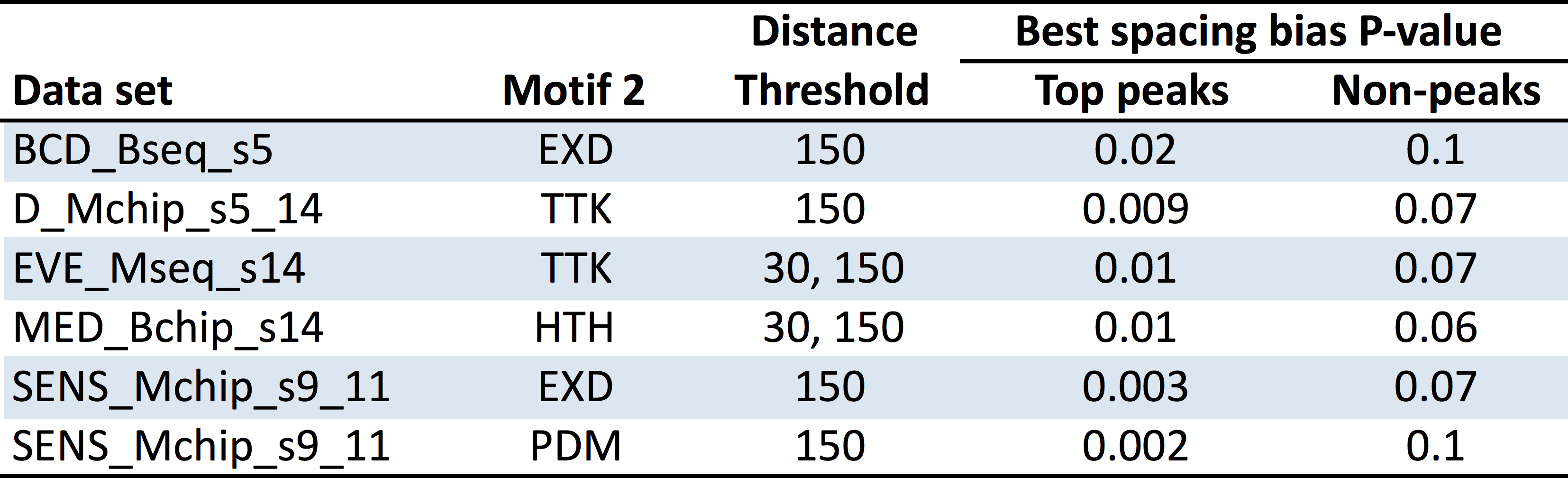

Supplement: Table S19 — Spacing bias analysis for antagonistic influences where the bias is significant in the peaks and not in non-peaks. Columns have semantics as in Table S5. (TIFF) [file pgen.1003571.s031.tiff]
